# Soluble galectin‐3 as a microenvironment‐relevant immunoregulator with prognostic and predictive value in lung adenocarcinoma

**DOI:** 10.1002/1878-0261.13505

**Published:** 2023-11-01

**Authors:** Susana Torres‐Martínez, Silvia Calabuig‐Fariñas, Andrea Moreno‐Manuel, Giulia Bertolini, Alejandro Herreros‐Pomares, Eva Escorihuela, Elena Duréndez‐Saéz, Ricardo Guijarro, Ana Blasco, Luca Roz, Carlos Camps, Eloisa Jantus‐Lewintre

**Affiliations:** ^1^ Molecular Oncology Laboratory Fundación Investigación Hospital General Universitario de Valencia Spain; ^2^ TRIAL Mixed Unit Centro Investigación Príncipe Felipe—Fundación Investigación Hospital General Universitario de Valencia Spain; ^3^ Centro de Investigación Biomédica en Red Cáncer CIBERONC Madrid Spain; ^4^ Department of Pathology Universitat de València Spain; ^5^ Tumor Genomics Unit Fondazione IRCCS Istituto Nazionale dei Tumori Milan Italy; ^6^ Department of Biotechnology Universitat Politècnica de València Spain; ^7^ Department of Surgery Universitat de València Spain; ^8^ Department of Thoracic Surgery Hospital General Universitario de Valencia Spain; ^9^ Department of Medical Oncology Hospital General Universitario de Valencia Spain; ^10^ Department of Medicine Universitat de València Spain; ^11^ Joint Unit: Nanomedicine Centro Investigación Príncipe Felipe—Universitat Politècnica de Valencia Spain

**Keywords:** biomarker, lung adenocarcinoma, sGAL‐3, T_REG_, tumorspheres

## Abstract

Despite the success of therapies in lung cancer, more studies of new biomarkers for patient selection are urgently needed. The present study aims to analyze the role of galectin‐3 (GAL‐3) in the lung tumor microenvironment (TME) using tumorspheres as a model and explore its potential role as a predictive and prognostic biomarker in non‐small cell lung cancer patients. For *in vitro* studies, lung adenocarcinoma (LUAD) and lung squamous carcinoma (LUSC) primary cultures from early‐stage patients and commercial cell lines were cultured, using tumorsphere‐forming assays and adherent conditions for the control counterparts. We analyzed the pattern of secretion and expression of GAL‐3 using reverse transcription–quantitative real‐time PCR (RTqPCR), immunoblot, immunofluorescence, flow cytometry, and immunoassay analysis. Our results using three‐dimensional (3D) models of lung tumor cells revealed that soluble GAL‐3 (sGAL‐3) is highly expressed and secreted. To more accurately mimic the TME, a co‐culture of tumorspheres and fibroblasts was used, revealing that GAL‐3 could be important as an immunomodulatory molecule expressed and secreted in the TME, modulating immunosuppression through regulatory T cells (T_REGS_). In the translational phase, we confirmed that patients with high expression levels of GAL‐3 had more T_REGS_, which suggests that tumors may be recruiting this population through GAL‐3. Next, we evaluated levels of sGAL‐3 before surgery in LUAD and LUSC patients, hypothesizing that sGAL‐3 could be used as an independent prognostic biomarker for overall survival and relapse‐free survival in early‐stage LUAD patients. Additionally, levels of sGAL‐3 at pretreatment and first response assessment from plasma to predict clinical outcomes in advanced LUAD and LUSC patients treated with first‐line pembrolizumab were evaluated, further supporting that sGAL‐3 has a high efficiency in predicting durable clinical response to pembrolizumab with an area under curve of 0.801 (*P =* 0.011). Moreover, high levels might predict decreased progression‐free survival and OS to anti‐PD‐1 therapy, with sGAL‐3 being a prognosis‐independent biomarker for advanced LUAD.

## Graphical abstract

This study found galectin‐3 (GAL‐3) could be an immunomodulatory molecule expressed and secreted in the lung tumor microenvironment modulating immunosuppression through regulatory T cells. A translational study using liquid biopsy revealed soluble GAL‐3 as a novel independent biomarker to predict clinical outcomes for surgery in early lung adenocarcinoma (LUAD) patients and also to predict the clinical outcomes for pembrolizumab in advanced LUAD patients.

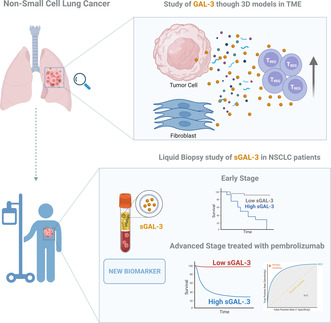

**Abbreviations**
3D: three‐dimensional
ACTB: beta‐actin
ADH: adherent
ATCC: American Type Culture Collection
AUC: area under curve
bFGF: fibroblast growth factor
BSA: bovine serum albumin
BV: Brilliant Violet
cDNA: complementary DNA
CI: confidence interval
CM: conditioned media
CR: complete response
CSCs: cancer stem cells
ctDNA: circulating tumor DNA
DCB: durable clinical benefit
EGF: epidermal growth factor
ELISA: enzyme‐linked immunosorbent assay
EVs: extracellular vesicles
FBM: fibroblast basal medium
FBS: fetal bovine serum
FFPE: formalin‐fixed, paraffin‐embedded
FR: first response assessment
GAL‐3: galectin‐3
HR: hazard ratio
IBs: immunoblots
ICBs: immune checkpoint blockers
IF: immunofluorescence
IQR: interquartile range
LGALS3BP: galectin‐3‐binding protein
LUAD: lung adenocarcinoma
LUSC: lung squamous carcinoma
NSCLC: non‐small cell lung cancer
ORR: overall response rate
OS: overall survival
PBMCs: peripheral blood mononuclear cells
PBS: phosphate‐buffered saline
PCA: principal component analysis
PD: progressive disease
PE: phycoerythrin
PFS: progression‐free survival
PR: partial response
PRE: pretreatment
PS: performance status
QC: quality controls
RECIST 1.1: Response Evaluation Criteria in Solid Tumors version 1.1
RFS: relapse‐free survival
RNA: ribonucleic acid
ROC: receiving operating curve
RT: reverse transcription
RTqPCR: reverse transcription–quantitative real‐time PCR
SD: stable disease
sGAL‐3: soluble galectin‐3
SPH: tumorspheres
SPSS: Statistical Package for the Social Sciences
TCGA: The Cancer Genome Atlas
TIICs: tumor‐infiltrating immune cells
TME: tumor microenvironment
TNM: tumor node metastasis
TPS: tumor proportion scores
TREGS: regulatory T cells

## 1 Introduction

Lung cancer is the second most diagnosed cancer in both men and women and the leading cause of cancer death worldwide [1]. Non‐small cell lung cancer (NSCLC) is the most represented of lung cancer cases (85 %), including lung squamous cell carcinoma (LUSC; ~ 30%), lung adenocarcinoma (LUAD; ~ 50%), and others (~ 20%) [2, 3]. On the other hand, in early stage, the first therapeutic option is the surgery, but the prognosis of NSCLC has gradually improved through advanced therapeutic approaches like neoadjuvant chemotherapy and immunotherapy [4]. However, significant percentage between 10–60% of patients relapse within 5 years after radical resection and frequently cases are diagnosed at advanced stages, when surgery is not possible [5]. Therefore, the identification of useful biomarkers through a non‐invasive approach to predict relapse is a priority. On the other hand, in advanced stages, the blockade of immune checkpoints has opened up a new standard of treatment for cancer patients, producing an effective antitumor response in tumor microenvironment (TME), concretely PD1/PDL1 axis inhibitors have been extensively studied and have drastically changed the therapeutic scenario for NSCLC with a plethora of clinical data demonstrating superior outcomes related to conventional therapies or molecular targeted therapy [6, 7, 8, 9]. However, the efficacy of cancer immunotherapy is limited by multiple immunosuppressive mechanisms present in TME. Therefore, the better comprehension of the interactions in TME between the immune system and tumor cells are necessary to develop new immunotherapeutic strategies more effective in NSCLC. The expression level of PD‐L1 on tumor cells or tumor‐infiltrating immune cells (TIICs) is considered the most available and implemented biomarker to select patients. However, significant percentage of PD‐L1‐positive NSCLCs cases do not respond to immune checkpoint blockers (ICBs), opposite a significant number of PD‐1‐negative tumors are sensitive to this therapy limiting its use in clinical practice [6, 10, 11]. Taking into consideration the abovementioned features, the identification of new reliable biomarkers, preferably tested in a minimal invasive manner, to guide patient selection and provide indications of efficacy and/or prognosis is a priority. In this line, exists intense interest in identifying predictive biomarkers derived from peripheral blood or minimal invasive samples. Some plasmatic biomarkers such as circulating tumor DNA (ctDNA) have been associated with clinical benefit and survival [12, 13]. However, the prognostic and/or predictive value of soluble plasma biomarkers in NSCLC have been sparsely validated in prospective studies and its role is not clearly understood.

Regarding TME, fibroblast, cancer stem cells (CSCs), tumor cells, and immune cells can interact contributing to immunosuppression. One important protein that contribute to TME immunosuppression is the glycoprotein galectin‐3 (GAL‐3). GAL‐3 is a carbohydrate‐binding protein that might have a crucial role promoting tumor growth and helping tumors to escape immune surveillance through immunosuppression [14]. In human genome GAL‐3 is coded by a single gene *LGALS3* which is suited on chromosome 14, locus q21–q2 [15]. Data have been shown that the intracellular Gal‐3 promoted tumor growth, metastasis and survival and the extracellular GAL‐3 may facilitate metastasis by promoting immune scape which has been poorly investigated [16, 17].

To study the TME, multiple three‐dimensional (3D) model systems have been proposed as new approaches to examine it, ranging from the simple co‐culture of cells in hydrogels, to complex multicomponent microfluidics, each with their own advantages and limitations [18]. Specifically, tumorspheres model provide an environment more similar to the tumor, with self‐imposed nutrient, with better immuno‐modulatory abilities and hypoxic gradients adding dimensions that not happened with monolayer 2D cell cultures [19].

Galectin‐3 could be an immunosuppressive molecule involved in tumor scape from immune surveillance with the TME implicated so we proposed to study the expression and secretion of GAL‐3 on 3D models of lung tumor cells analyzing its influence on T_REGS._ Moreover, as the clinical importance on recurrence of GAL‐3 after surgery in NSCLC patients has not been elucidated fully, we aimed to evaluate the prognostic and recurrence predictive value of soluble GAL‐3 (sGAL‐3) on these patients. Finally, taking into account that there is a necessity of looking for new reliable biomarkers for ICBs, the objective of this study it is not only analyzed the role of GAL‐3 in early patients but also in advanced patients to improve immune therapeutic strategies.

## 2 Materials and methods

### 2.1 Patients and plasma samples collection

This study included 137 individuals from the General University Hospital of Valencia divided in two different cohorts. Early cohort comprised 48 patients with early‐stage LUAD and 42 patients with early‐stage LUSC collected from July 2004 to September 2019. Plasma samples were obtained before surgery and selected by following eligibility criteria: candidate for surgical resection, non‐pretreated, over 18 years, non‐pregnant, stage I–IIIA (according to the American Joint Committee on Cancer staging manual), and with a histological diagnosis of NSCLC. Cryopreserved tumor tissue samples from 19 patients were used in this study. Data of expression of FOXP3, CD4, and CD8 in both tumor and stromal areas (via immunohistochemistry and RT‐qPCR) from these patients were collected from Usó M et al. [20]. Advanced cohort included 47 patients treated with first‐line pembrolizumab in monotherapy (200 mg every 21 days) (34 patients with advanced LUAD and 13 with advanced LUSC) (collected from February 2018 to July 2021) and fitted the following eligibility criteria: candidate for pembrolizumab treatment, non‐pretreated, over 18 years, non‐pregnant, irresectable stage IIIA‐IV (according to the American Joint Committee on Cancer staging manual), and with a histological diagnosis of NSCLC. According to guidelines, PD‐L1 expression ≥ 50% (assed by tumor proportion scores (TPS) and defined as the number of positive tumor cells divided by the total number of viable tumor cells multiplied by 100%) was present in tumor samples from all patients treated with pembrolizumab in monotherapy [21]. 34 plasma samples at pretreatment (PRE) were collected prior to the first administration of pembrolizumab and 25 plasma samples at first response assessment (FR) for LUAD advanced cohort and 13 samples at PRE were collected prior to the first administration of pembrolizumab and at FR for LUSC advanced cohort. All patients were followed up until December 2022. All peripheral blood samples were collected in 10 mL‐EDTA tubes plasma, were isolated by centrifugation at 4 °C and then stored at −80 °C until the analysis.

This study was conducted in accordance with the Declaration of Helsinki, and along with the protocol, were approved by the ethical review board of the General University Hospital of Valencia (No. 5/2015). All patients and healthy volunteers signed an informed consent for sample acquisition for research purposes before the beginning of this study.

### 2.2 Establishment of primary cell cultures

Following the tumor dissociation protocol previously described by our group surgical tumor specimens from patients were established as monolayers and tumorspheres [22]. For this study, three primary patient‐derived lung cancer long‐term cultures (PC301, PC435, and PC471) were employed. Tumor profiling of each patient‐derived culture was determined by next‐generation sequencing using Oncomine Focus Assay (Thermofisher Scientific, Waltham, MA, USA) and Ion GeneStudio S5 System (Thermofisher Scientific, Waltham, MA, USA) to get complete tumor profiling of each patient.

### 2.3 Commercial NSCLC and fibroblast cell lines

Fifteen human NSCLC cell lines, LUAD cell lines [A549 (RRID:CVCL_0023), NCI‐H1395 (RRID:CVCL_1467), NCI‐H1650 (RRID:CVCL_1483), NCI‐H1975 (RRID:CVCL_1511) NCI‐H1993 (RRID:CVCL_1512), NCI‐H2228 (RRID:CVCL_1543), NCI‐H23 (RRID:CVCL_1547), NCI‐H358 (RRID:CVCL_1559), HCC827 (RRID:CVCL_2063), PC9 (RRID:CVCL_B260)] and LUSC cell lines [SW900 (RRID:CVCL_1731), LUDLU‐1 (RRID:CVCL_2582), NCI‐H520 (RRID:CVCL_1566), NCI‐H1703 (RRID:CVCL_1490) and SK‐MES‐1 (RRID:CVCL_0630)] were used for *in vitro* experiments. LUAD cell lines were obtained from the American Type Culture Collection (ATCC, Manassas, VA, USA) and LUSC cell lines were kindly provided by J. Carretero (University of Valencia, Spain) unless SW900 that was purchased from ATCC. Immortalize primary fibroblast, CAF154‐hTERT cells are originally from cancer‐associated primary fibroblasts and were kindly provided by Luca Roz (Istituto Nazionale dei Tumori, Italy). The generation and the characteristics of them have been described previously [23]. All cell cultures (primary and commercial) were tested for mycoplasma before all the experiments. All human cell cultures were authenticated by short tandem repeat analysis with AmpFlSTR™ Identifiler™ Plus PCR Amplification Kit (Thermofisher Scientific, Waltham, MA, USA).

### 2.4 Cell culture conditions for tumor cells and fibroblast

Tumor cells were grown in RPMI‐1640 (commercial cell lines) or DMEM‐F12 (primary cultures) containing 10% fetal bovine serum (FBS), 100 μg·mL^−1^ penicillin/streptomycin, 0.001% non‐essential amino acids (for RPMI‐1640) and 2 mm l‐glutamine (for DMEM‐F12) (Gibco™, Grand Island, NY, USA). In order to obtain tumorspheres, when cells reached 80% confluence, they were trypsinized using 0.1% trypsin–EDTA (Corning, NY, USA). After that, cells were cultured at low density in ultra‐low attachment flasks (Corning, NY, USA) with serum‐free (RPMI‐1640/DMEM‐F12) medium supplemented with 0.4% bovine serum albumin (BSA), 50 μg·mL^−1^ epidermal growth factor, 20 μg·mL^−1^ basic fibroblast growth factor, insulin–transferrin–selenium PREMIX, 100 μg·mL^−1^ penicillin/streptomycin (P/S), and 2% B27 (Gibco™, Grand Island, NY, USA). The following experiments took place after 5 days when the cells started to grow and form floating aggregated. CAF154‐hTERT cells were grown in fibroblast basal medium (FBM) supplemented with the Kit‐Low serum (ATCC, Manassas, VA, USA). All cells were maintained at 37 °C in humidified atmosphere of 5% CO_2_ and 95% air.

### 2.5 Co‐cultures conditions

For co‐cultures, 3 × 10^5^ CAF154‐hTERT were cultured for 2 h with the proper medium in 6‐well plates. After 2 h, 1 × 10^5^ adherent or tumorspheres PC435 were cultured together with CAF154‐hTERT in 50% of FBM and 50% SPH DMEM F12 for 48 h. Conditioned media (CM) were collected from different conditions (tumorspheres PC435 or co‐culture tumorspheres PC435 + CAF154‐hTERT). CM will be used in the following experiment to test the effect on regulatory T cells (also called T_REG_).

### 2.6 PBMCs cultures and CM treatment

Human peripheral blood mononuclear cells (PBMCs) from nine healthy volunteers were plated at 1 × 10^6^ cells/well in 6‐well plates and incubated at 37 °C for 4 h. After the incubation, non‐adherent cells (T cells) were collected and used for the experiments. 1 × 10^7^ cells/well were treated with different CM collected from PC435 cultures and PC435 + CAF154‐hTERT co‐cultures. At the same time, the GAL‐3 monoclonal antibody (clone B2C10) (100 ng·mL^−1^) (Thermofisher Scientific, Waltham, MA, USA) were added to the culture in order to blocked sGAL‐3 in culture media to test its effect on the T_REG_ population.

### 2.7 Cellular pellets and supernatants collection

Both adherent cells and tumorspheres were seed at different densities for the following experiments (10 000 cell·mL^−1^ and 100 000 cell·mL^−1^) in 24‐well plates. Supernatant were collected at two time periods post‐seeded (12 h and 24 h) and stored at −80 °C until further analysis. Cell pellets were collected at the same points with TRIZol reagent (Invitrogen, Waltham, MA, USA) and frozen at −80 °C until the experiments for gene expression analysis.

### 2.8 Isolation of extracellular vesicles from cell cultures

To isolate tumor‐derived extracellular vesicles (EVs) from cultures, cells were grown in T175 cm^2^ flasks until 70–80% confluence for 72 h in 30 mL of FBS‐depleted media (in the case of tumorspheres cultures). After 72 h, detritus was eliminated by differential centrifugation at 500 ***g*** for 5 min, and then at 3000 ***g*** for 15 min. Subsequently, the supernatant was filtered through a 0.2‐μm filter (Corning, NY, USA) and ultracentrifuged at 110 000 ***g*** for 90 min (CP‐NX, P50AT2 Rotor; Hitachi, Japan). To wash the first pellet, second ultracentrifugation was performed; EVs were then resuspended in 30 mL of phosphate‐buffered saline (PBS). All centrifugations were performed at 4 °C. At last, EVs were resuspended in a tiny volume (30–60 μL) of filtered PBS and stored at −80 °C until the corresponding analysis.

### 2.9 Gene expression analysis

The extraction of total cellular ribonucleic acid (RNA) from cell pellets and frozen tissue samples was performed using standard TRIZol method according to manufactures' instructions. Exosomal total RNA derived from cell cultures was isolated using the Total RNA Purification Kit (Norgen Biotek, Thorold, ON, Canada). RNA concentrations were evaluated by Nanodrop (Thermofisher Scientific, Waltham, MA, USA). Reverse transcription–quantitative real time PCR (RTqPCR) was carried out to analyze the relative expression of *LGALS3* gene and reference genes on a Roche LightCycler®480 II system (Roche Ltd., Basel, Switzerland) (Table S1). Reverse transcription reactions were performed from 1.0 μg of total RNA [frozen tissue and formalin‐fixed, paraffin‐embedded (FFPE) samples] 0.5 μg of total RNA (cells samples) and 0.150 μg (EVs samples) using random hexanucleotides and a High‐Capacity complementary DNA (cDNA) Reverse Transcription Kit (Applied Biosystems, Waltham, MA, USA) according to the manufacturer's instructions. The resulting cDNA was used for RTqPCR reaction and was carried out with assays based on hydrolysis probes using 1 μL of cDNA, TaqMan Gene Expression Master Mix, and a TaqMan Gene Expression Assay (Applied Biosystems, Waltham, MA, USA) in final reaction volume of 5 μL. We used random‐primed qPCR Human Reference cDNA (Clontech, Mountain View, CA, USA) for efficiency calculations. Using genorm software (https://genorm.cmgg.be/ accessed on July 9, 2015) [24], *ACTB*, *GUSB*, and *CDKN1B* were selected as endogenous controls for cells and frozen tissue, whereas *ACTB* and *GAPDH* were selected as endogenous controls for EVs samples selected as endogenous controls using genorm software. Relative gene expression levels of *LGALS3* and *LGALS3BP* were calculated as the ratio of target gene expression to the geometric mean of the endogenous gene expressions according to Pfaffl formula [25]. All samples were tested in triplicate.

### 2.10 Immunoblot analysis

Tumorspheres were washed with cold PBS, whereas adherent cells were also scraped out of the dishes before lysis. Protein pellets were lysed using a lysis buffer composed of 100 mm Tris pH8, 2% NP40, 1% Na deoxicholate, 0.2% SDS and 300 mm NaCl, 1 mm sodium orthovanadate, 25 mm NaF and protease inhibitor cocktail (Roche, Basel, Switzerland). BCA Protein Assay (Thermofisher Scientific, Waltham, MA, USA) was employed to quantify the total protein concentration; 30 μg of total protein were separated on 12% SDS‐polyacrylamide gel and electro‐transferred to a 0.45 μm polyvinylidine difluoride membrane (MilliporeSigma, Burlington, MA, USA). The membrane was then blocked with 5% skim milk for 1 h and immunoblotted overnight at 4 °C with the Anti‐Galectin 3 antibody (Clone A3A12) (ab2785, Abcam, Cambridge, UK). Afterwards, membranes were incubated with anti‐IgG (whole molecule)‐Peroxidase secondary antibody (Thermo Fisher Scientific, Waltham, MA, USA) for 1 h at room temperature. Chemiluminescent detection with the high‐sensitivity Amersham ECL Select™ detection reagent (GE Healthcare, Chicago, IL, USA) was employed (Table S2). All results were normalized over β‐actin (Sigma‐Aldrich, St. Louis, MO, USA).

### 2.11 Flow cytometry analysis

To analyze tumor cell surface markers, single cell solution was washed in staining buffer (PBS1 × + 0.5% BSA+ 2 mm EDTA) and incubated for 30 min at 4 °C with phycoerythrin (PE) anti‐GAL‐3 (clone M3/38) (Biolegend, San Diego, CA, USA) (Table S2). For these analyses, dead cells were excluded using 7‐amino‐actinomycin D Viability Staining (Thermofisher Scientific, Waltham, MA, USA) (Table S2).

For analysis of Treg phenotype, T cells treated before with CM (tumorspheres or co‐culture) with and without GAL‐3 monoclonal antibody, were first incubated with surface antibodies in staining buffer for 30 min at 4 °C: Brilliant Violet V510 (BV510) Mouse Anti‐Human CD3 (Clone HIT3a), Brilliant Violet V421 (BV42) Anti‐Human CD4 (Clone SK3), Allofhycocyanin (APC) Anti‐Human CD25 (clone M‐A251); then fixed and permeabilized with Transcription Factor Buffer Set (Thermo Fisher Scientific, Waltham, MA, USA), according to the datasheet instructions, and finally incubated with PE anti‐Human FoxP3 (Clone 259D/C7) (all from BD Biosciences, Cambridge, UK) for 30 min at 4 °C (Table S2). T_REGS_ were identified within live cell gate as CD3 + CD4 + Foxp3 + CD25high. For these analyses, dead cells were excluded using Fixable Viability Stain 780 (BD Horizon, Franklin Lakes, NY, USA) (Table S2). Signal were acquired using a FC500 MPL Flow Cytometer and cytexpert v2.3 software (Beckman‐Coulter, Inc., Brea, CA, USA).

### 2.12 Immunofluorescence analysis

Cells were fixed in 4% paraformaldehyde in PBS at room temperature for 15 min, washed and permeabilized with 0.4% Triton X‐100 in PBS for 10 min, and washed again with PBS. Permeabilized cells were blocked with PBS containing 1% BSA for 1 h, and subsequently incubated with GAL‐3 anti‐mouse [1 : 200] (ab2785, Abcam, Cambridge, UK) antibody in blocking buffer overnight at 4 °C (Table S2). Thereafter, cells were washed with PBS and incubated with Alexa‐labeled IgG secondary antibodies containing blocking buffer for 1 h. Slides were incubated with 4′,6‐diamidino‐2‐phenylindole for 3 min, mounted with Fluoromount Aqueous Mounting Medium (Sigma‐Aldrich, St. Louis, MO, USA), and analyzed using a Leica confocal microscope (Leica Microsystems, Buffalo Grove, IL, USA).

### 2.13 *In silico* analysis

First, an *in‐silico* analysis was carried out using two lung cancer data sets from The Cancer Genome Atlas (TCGA) consortium to study the expression of GAL‐3 in early NSCLC patients [26, 27]. RNA‐sequencing (Ilumina Hi Seq platform) and clinical information was downloaded from the ICGC Data Portal, https://dcc.icgc.org/releaes/current/projects/LUAD‐US and https://dcc.icgc.org/releases/current/projects/LUSC‐US [28]. The limma package from Bioconductor was used to obtain normalized RNA‐seq data. Linear fit model for LGALS3 was obtained before constructing the different contrast matrixes. Given the linear models, empirical Bayes statistics were computed for differential expression analysis.

### 2.14 Immunoassay based on Luminex xMAP

Supernatants of cell cultures or plasma samples were assayed through multiplex magnetic bead‐based immunoassay technology based on flow cytometry using Human Circulating Cancer Biomarker Magnetic Bead Panel 3, 96 Well Plate Assay, Cat. # HCCBP3MAG‐58K and Human Immuno‐Oncology Checkpoint Protein Panel 2 – Immuno‐Oncology Multiplex Assay, Cat. #HCKP2‐11K (Merck Millipore, Billerica, MA) to quantify levels of GAL‐3 produced by tumor cells in the culture medium and in plasma, respectively. Quality controls (QC1 and QC2), as well as a calibration curve based on 1 : 4 dilutions of the highest standard were used for quantification and as internal controls for intra‐ and inter‐assay reproducibility. Briefly, 25 μL of culture medium or plasma samples (diluted 1 : 2) were used for each sample and mixed with proper regents and monoclonal antibody to human GAL‐3, which are covalently bound to the surface of magnetic microspheres dyed with accurate amounts of red and infrared fluorophores in order to produce a single spectral signature which can be detected in the Luminex platform (Luminex Corp, Austin, TX). sGAL‐3 quantification is determined by the fluorescently labeled secondary antibody whose signal intensity is proportional to the detected analyte concentration. Fluorescent signal of all samples was read on a Luminex 100/200™ instrument (Luminex Corp). Based on the measurements of seven diluted standard concentrations provided by the manufacturer, a five‐parameter standard curve was used to convert optical density values into concentrations (pg·mL^−1^). Data for minimum of 50 beads per cytokine were collected for each standard and sample. The final concentrations (expressed in pg·mL^−1^) were calculated using belysa™ software (Merck Millipore, Billerica, MA). All inter‐assay and intra‐assay coefficients of variation were below 15%. The lower limit of quantification of GAL‐3 for HCCBP3MAG‐58K was 4 pg·mL^−1^ and for HCKP2‐11 K was 48.8 pg·mL^−1^.

### 2.15 Exploratory endpoints patients evaluation

Patients' clinical and follow‐up data were abstracted from medical records. Exploratory endpoints for early cohort were relapse‐free survival (RFS) and overall survival (OS) according to plasma concentrations of GAL‐3. RFS and OS were described as the interval before diagnostic to the endpoint (objective disease relapse and death, respectively) or last follow‐up. Exploratory endpoints for advanced cohort were overall response rate (ORR) evaluated using the Response Evaluation Criteria in Solid Tumors version 1.1 (RECIST 1.1) and defined as the proportion of patients achieving complete (CR) or partial response (PR), stable disease (SD), and progressive disease (PD); durable clinical benefit (DCB; CR, PR, or SD lasting 6 months or more after initiation of pembrolizumab treatment) and non‐DCB (PD within 6 months after treatment start), progression‐free survival (PFS) and OS, according to plasma concentrations of GAL‐3. PFS and OS were described as the interval from the beginning of pembrolizumab treatment to the endpoint (objective disease progression and death, respectively) or last follow‐up.

### 2.16 Data acquisition and analysis of tumor infiltration immune cells by CIBERSORTx

We acquired a LUAD data set from the TCGA consortium. Clinical and RNA‐sequencing (Illumina HiSeq platform) information was directly downloaded from the ICGC Data Portal [28] (https://dcc.icgc.org/projects/LUAD‐US), and only patients who fit the eligibility criteria (pathology‐confirmed LUAD and stage I‐IIIA) were included for further analysis.

We prepared and uploaded the mixture dataset according to the instructions of CIBERSORTx online analysis platform (https://cibersortx.stanford.edu/). To deconvolve immune cell subsets, we used the LM22 signature matrix, which is a validated leukocyte gene signature matrix that contains 547 genes distinguishing 22 human hematopoietic cell phenotypes, including seven T‐cell types, naïve and memory B cells, plasma cells, natural killer cells, and myeloid subsets [29]. We selected “B‐mode” for batch correction and we set permutations to 500. Other parameters retained the default.

After running CIBERSORTx, we obtained the absolute proportions of subsets of TIICs in each sample with *P*‐values measuring the confidence of the results for the deconvolution. All samples were considered eligible for having *P* < 0.05. Dataset from CIBERSORTx of all samples is shown in Fig. S1. Heatmap of different cellular subtypes is presented in Fig. S2. Based on our previous analysis, only the proportions of T_REGS_, T cells CD4 memory activated, T cells CD8, macrophages M1, and macrophages M2 were considered in the subsequent exploratory analyses. Exploratory analyses were performed in r (version 4.3.0) using *k*‐means clustering and principal component analysis (PCA). In addition, we analyzed the RNA‐seq data of counts for 356 LUAD patients obtained from TCGA. Patients were grouped into high and low groups by median of *LGALS3*.

### 2.17 Statistical analysis

For cell culture experiments, triplicate tests were carried out for each sample. Results are expressed as median ± interquartile range (IQR). Expression and secretion of paired adherent cells and tumorspheres were analyzed using non‐parametric Wilcoxon's signed‐rank test. The comparison of median GAL‐3 levels between groups was performed using non‐parametric Mann–Whitney *U*‐test and Kruskall–Wallis to compare continuous variables. A Spearman rank test was used to test for correlations between continuous variables. The association between discrete variables were evaluated by the *χ* ^2^ tests. Graphs comparing metrics across groups show the median and the IQR, assuming non‐normally distributed data. Receiving operating curve (ROC) method was used to determine a cut‐off level of sGAL‐3 for ORR and DCB. Other predictive parameters were also evaluated, including sensitivity, specificity, cut‐off value, positive predictive value, negative predictive value, and area under the ROC curve (area under curve, AUC) with 95% confidence interval (CI), to assess the discrimination power of sGAL‐3. Survival analyses were performed using univariate Cox regression analysis and Kaplan–Meier (logrank) test method with dichotomized sGAL‐3 levels and clinicopathological variables. To analyze the independent value of the GAL‐3, a Cox proportional hazard model for multivariate analyses was used. All significant variables from the univariate were entered into the multivariate analyses in a forward stepwise Cox regression analysis. Statistical analyses were performed using the Statistical Package for the Social Sciences (spss, Chicago, IL, USA) version 23.0. Statistical significance was set at *P* < 0.05 (*), *P* < 0.01 (**), *P* < 0.001 (***).

## 3 Results

### 3.1 Generation of lung tumorspheres from NSCLC patients and cell lines

In our laboratory, short‐term patient‐derived cultures were successfully established in 40% of the cases as described in Herreros‐Pomares et al. [22]. In this work we employed three long‐term patient‐derived cultures, PC301, PC435, and PC471 which were able to grow tumor cells as monolayer and tumorspheres. Clinicopathological features from PC301, PC435, and PC471 are summarized in Table 1. Long‐term primary patient‐derived lung cancer cell cultures were established for 1 month before they were split for the first passage. No significant association were found between the establishment of primary cultures and clinicopathological variables. The morphology of cells from patient‐derived cultures and cell lines was examined presenting heterogeneity on the adherent‐cultures cells between samples. Regarding tumorspheres, tight spheroids were formed by HCC827, H1395, H23, H1650, H358, H2228 PC435, PC471, and PC301 whereas H1993, A549, PC9, H520, SK‐MES‐1, and H1703 formed loose and irregularly shaped, and SW900, LUDLU‐1, and H1975 showed a mixed behavior (Fig. S3). All these cell lines and primary cultures were included in further gene and protein expression analyses. Analysis will be done separating LUAD from LUSC cell cultures.

**Table 1:** Clinicopathological characteristics of the patients included in the study. DFS, disease‐free survival.

Patient code	Gender	Age	TNM stage	Histology	Smoking status	Progression/Exitus	DFS (months)	Mutational status
435	Male	73	IIB	LUAD	Former	NO	24	KRAS p.G12C, PIK3CA p.H1047R
471	Female	85	IIA	LUAD	Never	NO	27	PIK3CA p.D538N
301	Male	71	IIB	LUSC	Former	NO	75.50	PIK3CA p.G118D
								TP53 p.S261V*fs84

### 3.2 LUAD tumorspheres express high levels of LGALS3 related to immunoregulation

**Fig. 1:**
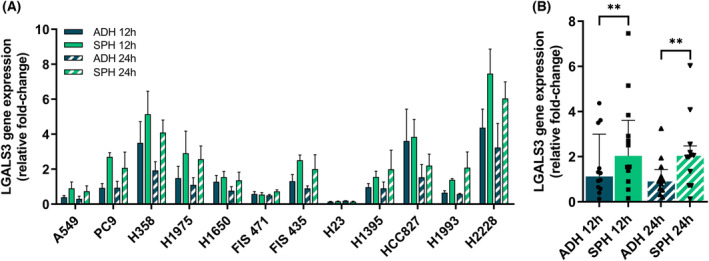
Transcription levels of *LGALS3* in tumorspheres versus adherent LUAD primary cultures and cell lines analyzed by RTqPCR at 12 and 24 h after cell seeding. (A) The results shown the relative fold‐change gene expression of LGALS3 to reference genes *ACTB*, *CDKN1B*, and *GUSB*. Errors bars represent standard deviation (SD) of three different experiments. (B) The results shown are the median of relative fold‐change gene expression of *LGALS3* to reference genes *ACTB*, *CDKN1B*, and *GUSB*. Statistical analysis was carried out with the Wilcoxon test. Errors bars represent IQR of all samples (*n* = 12). Significance values were **P ≤ 0.01. ADH, adherent; *n*, sample size; SPH, tumorspheres.

The expression at mRNA of *LGALS3* described as an immunoregulatory factor was analyzed in tumorspheres and adherent cells from LUAD and LUSC of three patient‐derived cells and 15 cell lines using RTqPCR. No statistical difference between cell lines with *EGFR* and *KRAS* driver mutations and the expression of LGALS3 were found. LUAD tumorspheres showed significantly higher expression of *LGALS3* compared to adherent‐cultures cells in both conditions at 12 h and 24 h post‐seeded according to Wilcoxon's signed‐rank test in all primary cultures and cell lines (*P* = 0.004 and *P* = 0.003, respectively; Fig. 1A,B). However, no significant differences in the expression of *LGALS3* between tumorspheres and adherent cells were shown in LUSC cell cultures (Fig. S4). Next, we analyzed the gene expression levels of GAL‐3‐binding protein (LGALS3BP) and its correlation with gene expression levels of *LGALS3*. LUAD tumorspheres showed significantly higher expression of *LGALS3BP* compared to adherent culture cells in both conditions at 12 h and 24 h post‐seeded according to Wilcoxon's signed‐rank test in all primary cultures and cell lines (Fig. S5A,B). Moreover, the expression of GAL‐3‐binding protein was correlated with the expression of GAL‐3 in LUAD cell cultures in both conditions at 12 h and 24 h post‐seeded both in adherent cells and tumorspheres (*R* = 0.62, *P* = 0.0014 and *R* = 0.64, *P* = 0.00095, respectively) (Fig. S5C,D).

**Fig. 2:**
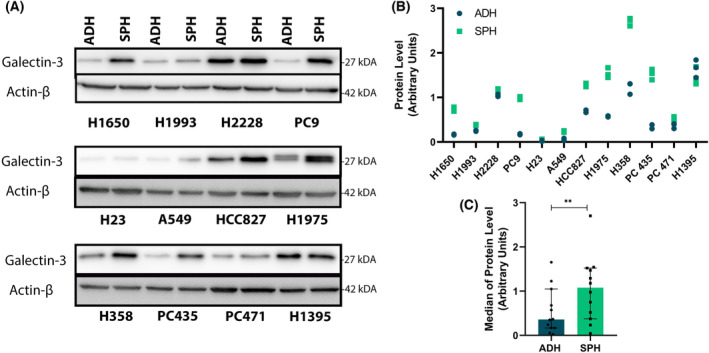
Expression of GAL‐3 as protein level. (A) IBs showing the level of GAL‐3 in adherent cells and tumorspheres. Beta‐Actin (ACTB) was used as loading control. The experiment was repeated three times and representative western blot results from one experiment were shown. (B) imagej analysis of IBs of panel a. Bar chart represents the relative expression of each protein according to IBs. Three gray values relative to the loading controls were measured in every case and averaged. (C) Values relative to the loading controls were measured in every cell line and averaged. Statistical analysis was carried out with the Wilcoxon test. Errors bars represent IQR of all cell lines and primary cultures median (*n* = 12). Significance values were **P < 0.01. ADH, adherent; *n*, sample size; SPH, tumorspheres.

**Fig. 3:**
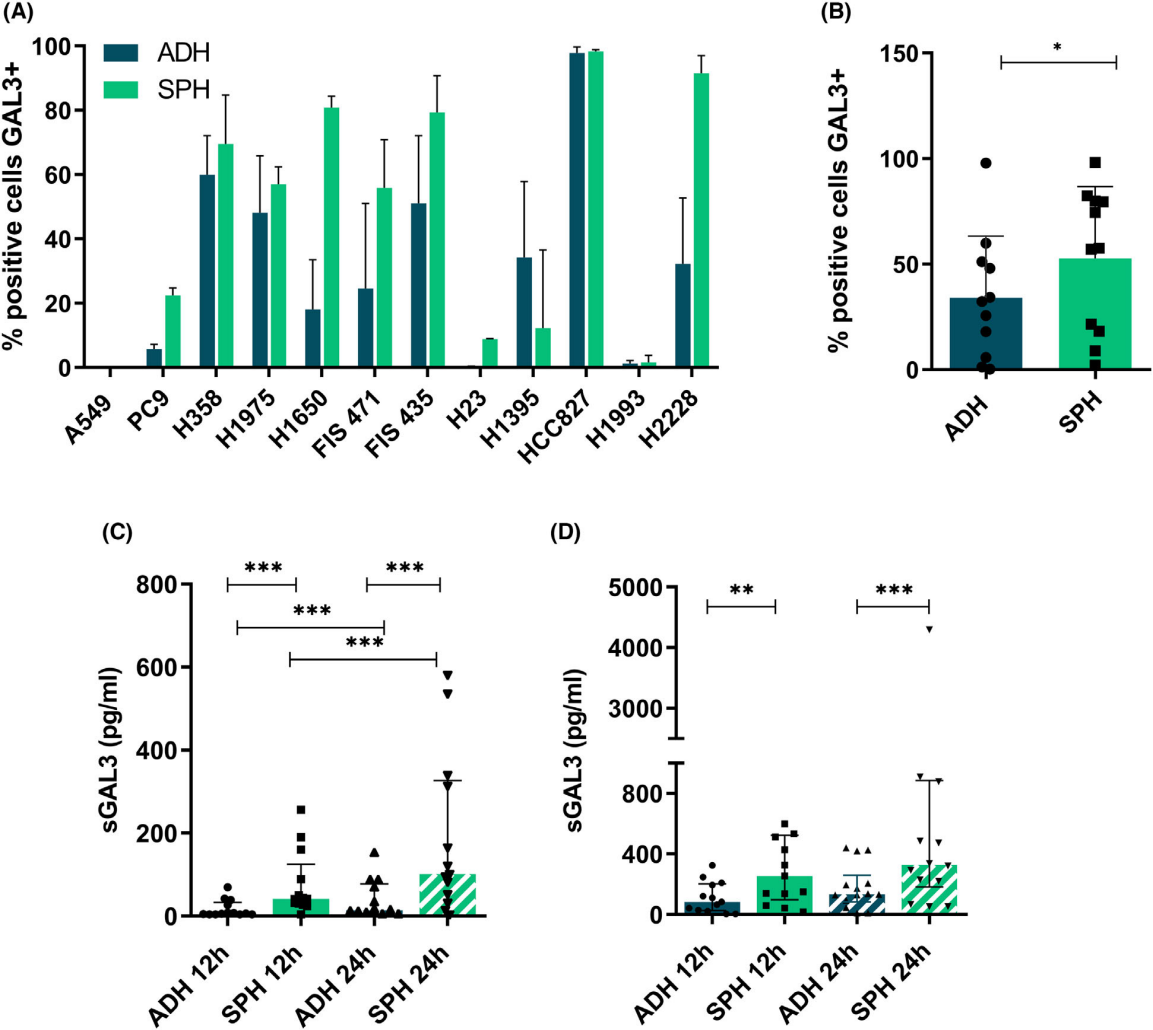
Flow cytometry and Immunoassay analysis of GAL‐3 in LUAD cells. (A, B) Flow cytometry analysis of surface GAL‐3 in LUAD adherent cells and tumorspheres. (A) The results shown are individual results for each cell line and primary culture. Errors bars represent SD of three different experiments. (B) The results shown are the median of all cells lines and primary cultures. Statistical analysis was carried out with the Wilcoxon test. Errors bars represent IQR of the median. (C,D) Immunoassay of sGAL‐3 in LUAD adherent cells and tumorspheres analyzed by Luminex® Technology at 12 and 24 h after cell seeding. (C) Median levels of sGAL‐3 of all cell lines and primary cultures at 12 and 24 h after 10 000 cells·mL^−1^ seeding (low density). (D) Median levels of sGAL‐3 of all cell lines and primary cultures at 12 and 24 h after 100 000 cells·mL^−1^ seeding (high density). Statistical analysis was carried out with the Wilcoxon test. Errors bars represent IQR of the median of all cell lines and primary cultures (*n* = 12). Significance values were *P ≤ 0.05, **P ≤ 0.01, ***P ≤ 0.001. ADH, adherent; *n*, sample size; SPH, tumorspheres.

Gene expression analyses were complemented with protein expression levels analyses by means of different experiments. GAL‐3 was significantly higher in tumorspheres than in adherent cells in most of LUAD cells according to IB with only one cell line (H1395) exception (Fig. 2). Original and complete immunoblots (IBs) are found in Fig. S6. Interestingly, at membrane level, LUAD tumorspheres were highly enriched in GAL‐3+ cells (*P* = 0.021) (Fig. 3A,B). Moreover, LUAD tumorspheres secreted significantly higher levels of sGAL‐3 than adherent cells at 12 h and 24 h post‐seeded at low and high cell density (Fig. 3C,D). According to RTqPCR analysis, in terms of protein levels, H23 and A549 show the lowest expression levels of Gal‐3 as well. We did not find significantly differences in LUSC cells (Fig. S7).

**Fig. 4:**
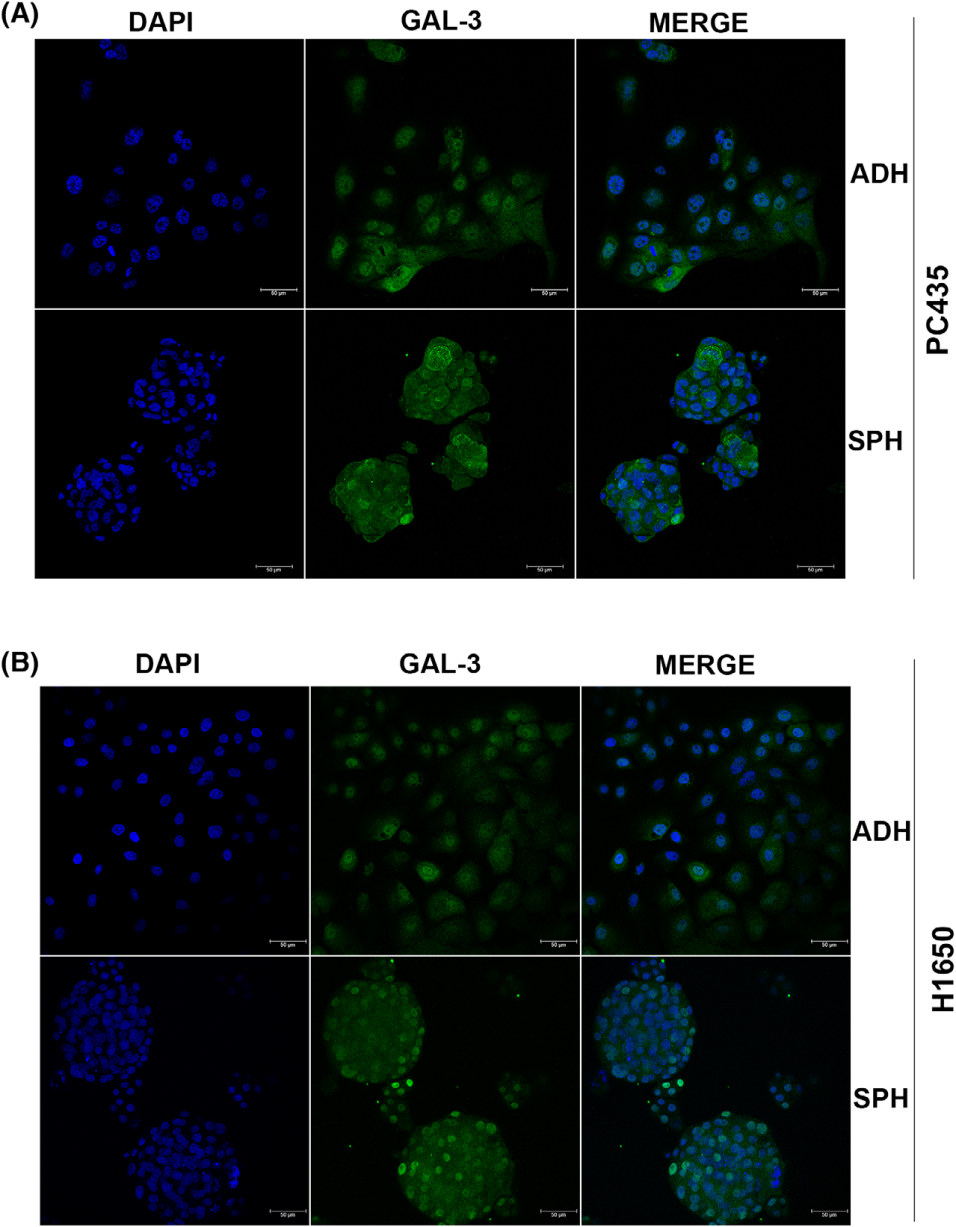
Representative IF images of GAL‐3 in adherent‐cultured cells and tumorspheres from (A) PC435 and (B) H1650. Immunofluorescence green channel shows the indicated GAL‐3 staining, blue channel shows DAPI staining, and merge shows all channels merged. The experiment was repeated three times and representative IF image from one experiment were shown. Scale bar represents 50 μm. ADH, adherent; SPH, tumorspheres.

Interestingly, differential subcellular localization of GAL‐3 (membranous, nuclear, and cytoplasmatic) was observed without significant differences between lung tumorspheres and adherent cells by immunofluorescence (IF) (Fig. 4). No signal was detected in A549 and H23, in accordance with low expression and low secretion levels detected previously (Fig. S8).

### 3.3 LUAD tumorspheres‐derived EVs express high levels of GAL‐3 in correlation with LUAD tumorspheres cell cultures

The *LGALS3* expression was examined in a larger number of EVs samples from NSCLC cell cultures (adherent vs tumorspheres conditions) using quantitative RT‐PCR (RT‐qPCR).

**Fig. 5:**
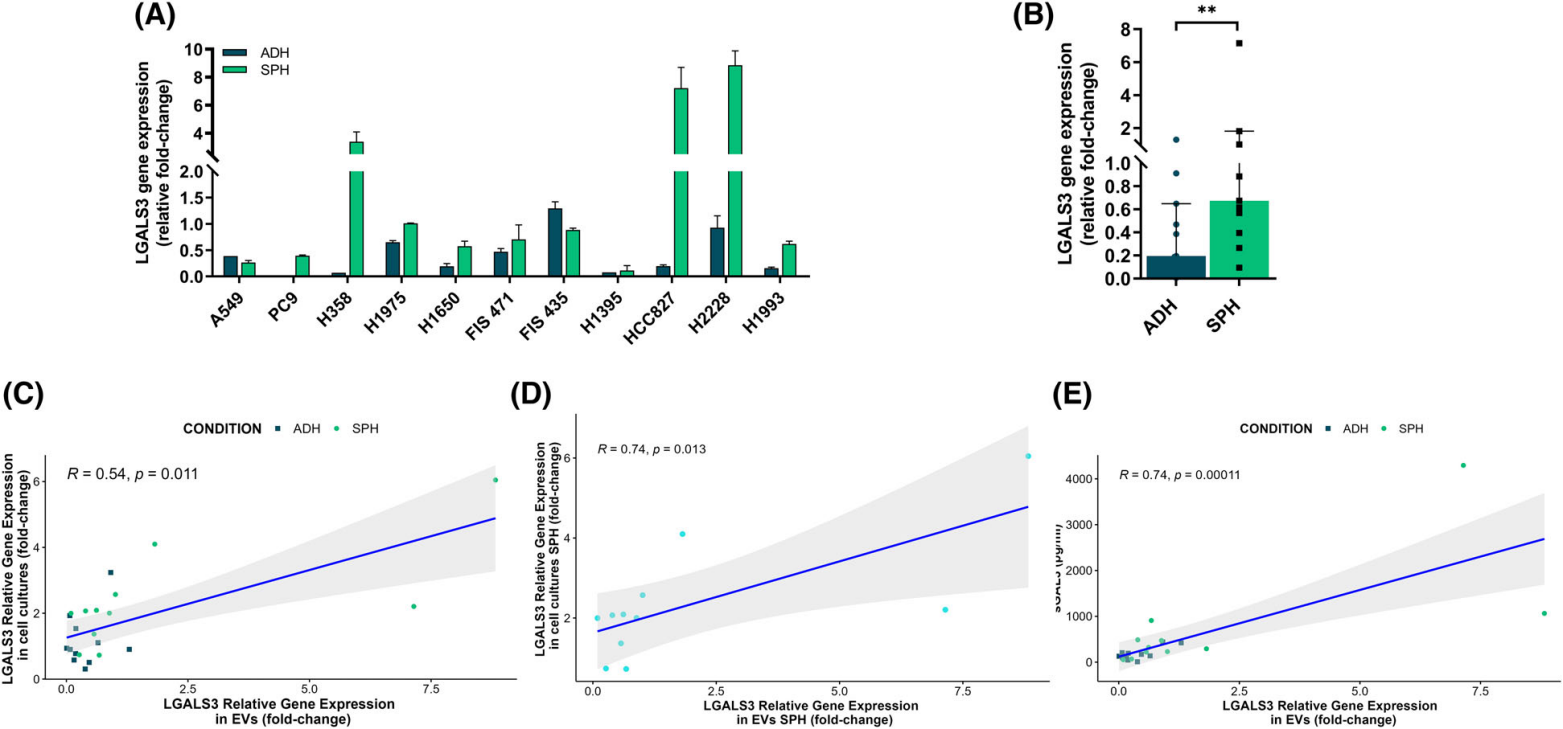
*LGALS3* expression in LUAD tumor‐derived EVs from tumorspheres and adherent cells and correlation with expression of *LGALS3* and secretion of GAL‐3 in culture cells. (A) The results shown the relative fold‐change gene expression of *LGALS3* in LUAD tumor‐derived EVs to reference genes *ACTB*, *CDKN1B*, and *GUSB*. Experiments were performed in duplicate. (B) The results shown are the median of relative fold‐change gene expression of LGALS3 in LUAD tumor derived‐EVs to reference genes *ACTB*, *CDKN1B*, and *GUSB*. Statistical analysis was carried out with the Wilcoxon test. Errors bars represent IQR of all samples (*n* = 11). (C) Correlation between *LGALS3* expression levels in LUAD tumor derived‐EVs and *LGALS3* expression levels in LUAD tumor cell cultures (*n* = 11). (D) Correlation between *LGALS3* expression levels in LUAD tumor derived‐EVs from spheroids and *LGALS3* expression levels in LUAD tumorspheres cell cultures (*n* = 22). (E) Correlation between *LGALS3* expression levels in LUAD tumor derived‐EVs and sGAL3 levels secreted by LUAD tumor cell cultures (*n* = 11). Statistical analysis was carried out with the Spearman Correlation Coefficient. *R* represents the Spearman correlation coefficient. Significance values were **P ≤ 0.01. ADH, adherent; *n*, sample size; SPH, tumorspheres.

Employing this technique, in concordance with our previous study, it was confirmed that *LGALS3* presented significantly higher expression in LUAD secreted‐EVs derived from tumorspheres than LUAD secreted‐EVs derived from adherent cells (*P* = 0.001) (*N* = 11, Fig. 5A,B), while there were no significant differences of *LGALS3* in the LUSC group (*N* = 6). The expression of GAL‐3 in LUAD cell‐derived EVs was correlated with the expression of GAL‐3 in LUAD cell cultures (*R* = 0.54, *P* = 0.011) and even more correlated when we analyze only the subgroup of spheres (*R* = 0.74, *P* = 0.013) (Fig. 5C,D). Moreover, a strongly correlated with the secretion of sGAL‐3 in LUAD cell cultures was observed (*R* = 0.74, *P* = 0.00011) (Fig. 5E). No significant correlations were found for LUSC group.

### 3.4 Galectin‐3 as an immunoregulatory factor responsible to increase regulatory T cells (T_REGS_)

**Fig. 6:**
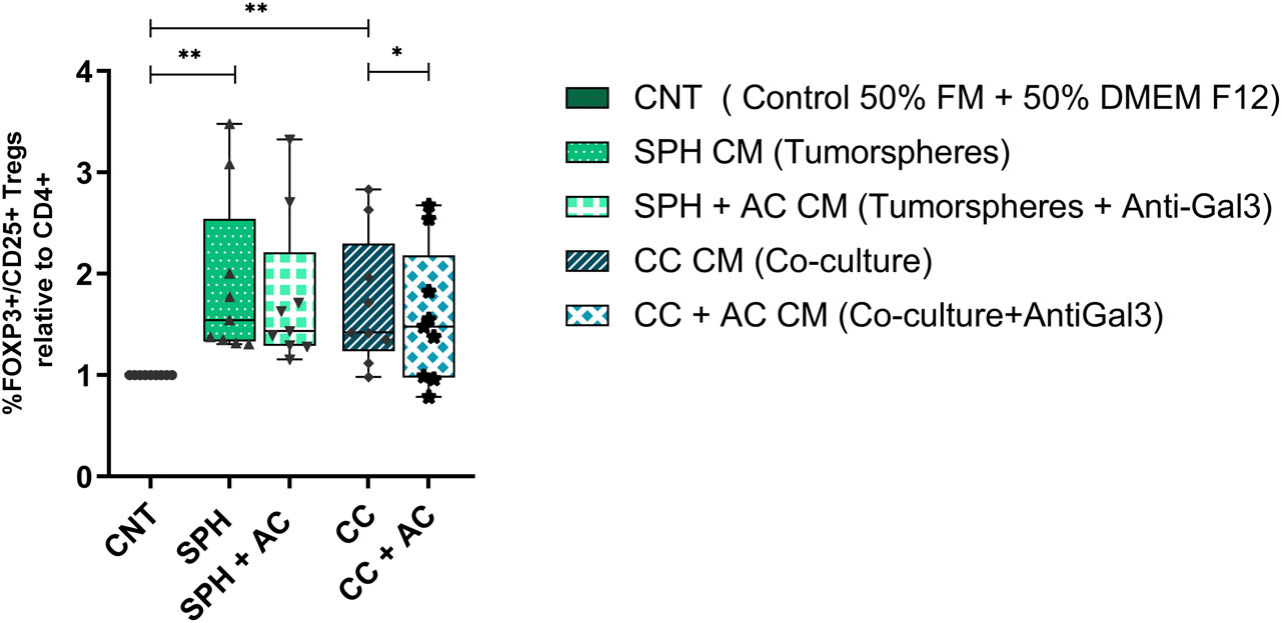
CM from spheroids induces T_REGS_ that can be prevented by Gal‐3 blockade. Flow cytometry analysis for T_REG_ population within T lymphocytes (T_REG_: CD4 + Foxp3 + CD25+), from *n* = 9 healthy volunteers. T lymphocytes were incubated for 72 h with CM from spheroids or co‐culture, untreated or treated with anti‐ Gal‐3 antibody. Data are the median value in % T_REG_ population FOXP3^+^/CD25^+^ relative to CD4+. We used as a control (CNT) (50% of FBM and 50% tumorspheres DMEM F12). Statistical analysis was carried out with the Wilcoxon test. Bars represent minimum and maximum points. Significance values were *P ≤ 0.05, **P ≤ 0.01. CM, conditioned medium; CNT, control medium; *n*, sample size; SPH, tumorspheres.

To functionally test the relevance of effects on T_REGS_ induced by GAL‐3, the ability of CM collected from tumorspheres, and the co‐culture (tumorspheres+fibroblasts) treated or not with the blocking GAL‐3 monoclonal antibody were tested. So, the effects of CM from tumorspheres and co‐cultures in modulating T cells having regulatory function (T_REGS_: CD4^+^Foxp3^+^CD25^+^) were assessed. Tumorspheres CM and co‐culture CM were able to increase the percentage of T_REGS_ compared to control (1.9‐ and 1.7‐fold increase, *P* = 0.008 and *P* = 0.011, respectively). Remarkably, blockade of Gal‐3 in co‐culture CM was sufficient to prevent the increase of T_REGS_ population significantly (*P* = 0.028) (Fig. 6).

### 3.5 Correlation between *LGALS3* expression in tumor with FOXP3, CD4, and CD8

**Fig. 7:**
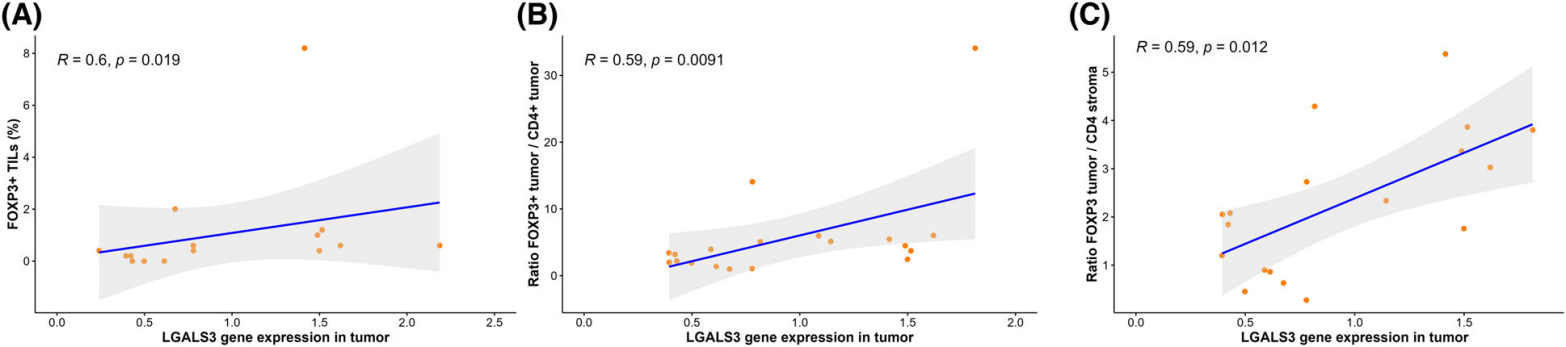
Correlations between T‐cell markers in tumor or stroma compartments from FPEE samples and *LGALS3* expression levels in frozen tumor tissue. (A) Correlation between *LGALS3* expression levels in tumor and FOXP3+ infiltration in tumor (*n* = 15). (B) Correlation between *LGALS3* expression levels in tumor and *FOXP3* tumor/CD4 tumor ratio (*n* = 19). (C) Correlation between LGALS3 expression levels in tumor and FOXP3 tumor/CD4 stroma ratio (*n* = 19). Statistical analysis was carried out with the Spearman Correlation Coefficient. *R* represents the Spearman correlation coefficient. *P*‐value < 0.05 was statistically significant.

Next, we aimed to delve deeper into the relationship between GAL‐3 and various T–cell markers, including FOXP3 (the most specific Treg marker), in a more translational manner. To achieve this, we correlated the expression of GAL‐3 in frozen tumor samples with the infiltration of FOXP3+, CD4+, and CD8+ lymphocytes as well as the expression of these markers in FPEE from tumor and tumor‐near stroma compartment. First of all, the number of positive cells per high‐powered field in the stromal compartment ranged from 0 to 21 for FOXP3, from 0 to 37 for CD4, and from 9 to 55 for CD8. On the other hand, in the tumor compartment, the number ranged from 0 to 8 for FOXP3, from 0 to 12 for CD4, and from 1 to 24 for CD8. We have observed a positive correlation between those patients with high FOXP3+ infiltration in tumor and those with high expression of *LGALS3* in tumor (*R* = 0.6, *P* = 0.019) (Fig. 7A). No other correlations were found with the other T–cell markers.

Then, we evaluated the correlation between expression of *LGALS3* in tumor and gene expression levels of *FOXP3*, *CD4*, and *CD8* in tumor and stroma area samples that were microdissected from FFPE samples. Results of correlations with individual genes were not significant. Next, we try to combine these genes in order to find correlation with *LGALS3* expression. We decided to combine T–cell markers such as *CD4* (a T helper cell marker), and *CD8* (a T cytotoxic cell marker) in combination with *FOXP3*. We calculated new variables based on the ratio of these markers. From the different combinations that were correlated with *LGALS3* expression in tumor, we found that the ratio between *FOXP3* expression assessed in the tumor compartment and the expression of *CD4* in the stroma and tumor compartment correlates positively and significantly with *LGALS3* expression in tumor (*R* = 0.59, *P* = 0.012, and *R* = 0.59, *P* = 0.0097, respectively). In particular, those patients with high FOXP3 expression levels in the tumor compartment and low *CD4* levels in the tumor or in the stroma had higher levels of *LGALS3* in tumor (Fig. 7B,C). No other significant correlations were found in the remaining combinations.

### 3.6 *LGALS3* expression and patient‐clusters based on different immune cell infiltration

**Fig. 8:**
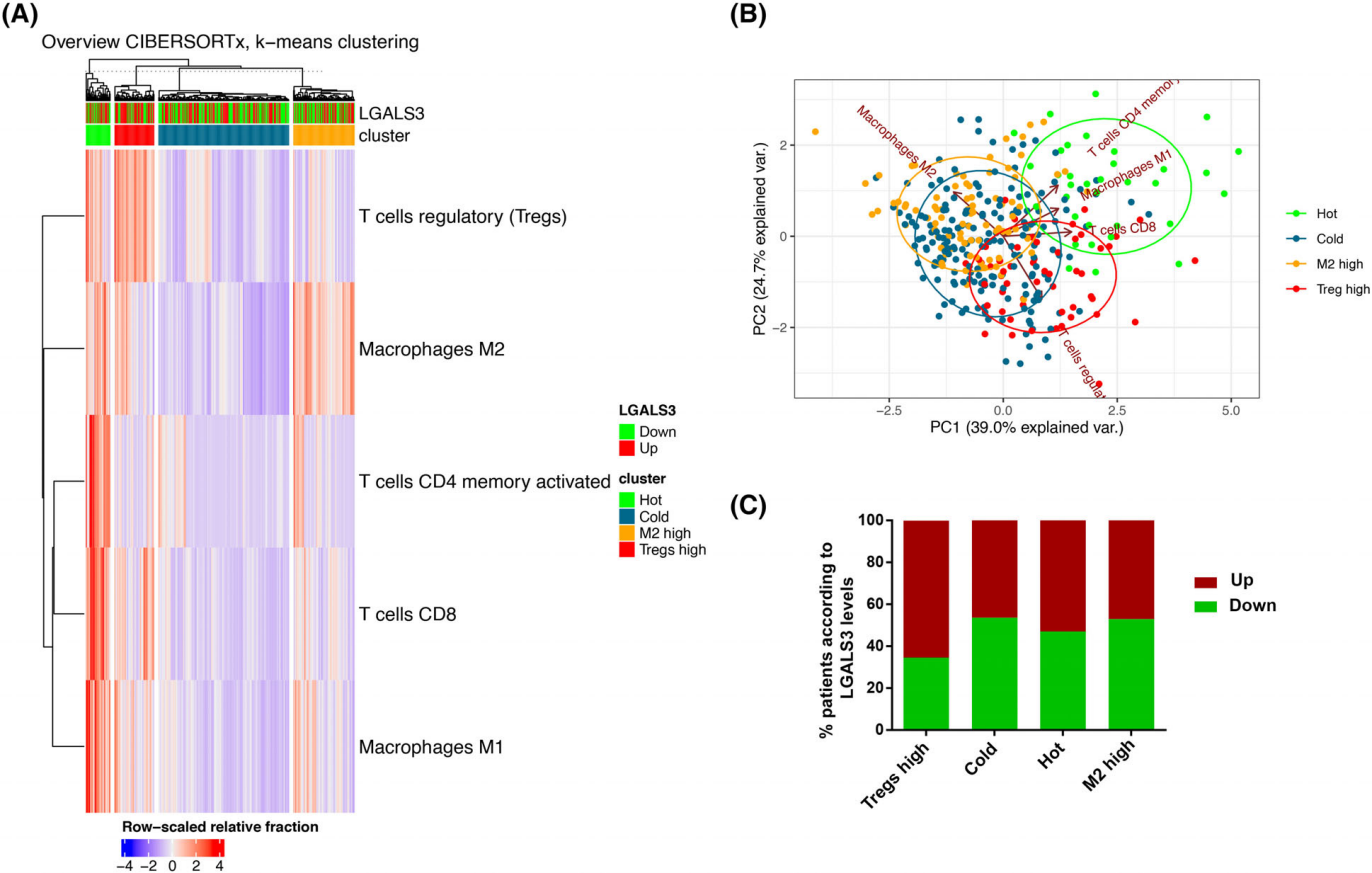
Results of immune cell infiltration clustering and expression of *LGALS3*. (A) K‐means heatmap. Four distinctive clusters of patients (*n* = 356) were identified by using hierarchical clustering algorithm with ComplexHeatmap package based on different immune cell infiltration. Clusters are distinguished by hot tumors (Hot), cold tumors (Cold), M2‐enriched tumors (M2 high), and regulatory T‐cell‐enriched tumors (Treg high). More red color designates higher expression for a given sample while blue designates lower expression. *LGALS3* expression is shown on top. Red color represents overexpression and green represents underexpression. (B) The scatterplot performed by PCA to show the four distinct clusters. (C) Bar charts representing the percentage of patients with upregulated *LGALS3* and downregulated *LGALS3* in the four clusters.

Next, to validate the relationship between *LGALS3* expression and different cellular subtypes, including T_REGS_, which are of interest to us, we used the CIBERSORTx platform in a patient cohort from TCGA. This study was performed considering the proportion of T_REGS_, T cells CD4 memory activated, T cells CD8, macrophages M1, and macrophages M2 in the tumors of 356 resectable LUAD patients. Based on these lymphocytes subset profiles, we identified four distinctive subgroups by using k‐means clustering: Hot tumors, Cold tumors, M2 high tumors, and T_REGS_ high tumors (Fig. 8A). A scatterplot of the four clusters conducted by PCA is displayed in Fig. 8B. We further explore the association of patient‐clusters and *LGALS3* expression. As displayed in the Fig. 8A, there is a trend showing that tumors with a high proportion of T_REGS_ have a higher percentage of patients with an upregulation of *LGALS3*, although not significant. Specifically, 65.45% of the patients in this cluster have upregulated GAL‐3 (Fig. 8C).

### 3.7 Analysis of prognostic value of GAL‐3 in early‐stage NSCLC cohort

**Fig. 9:**
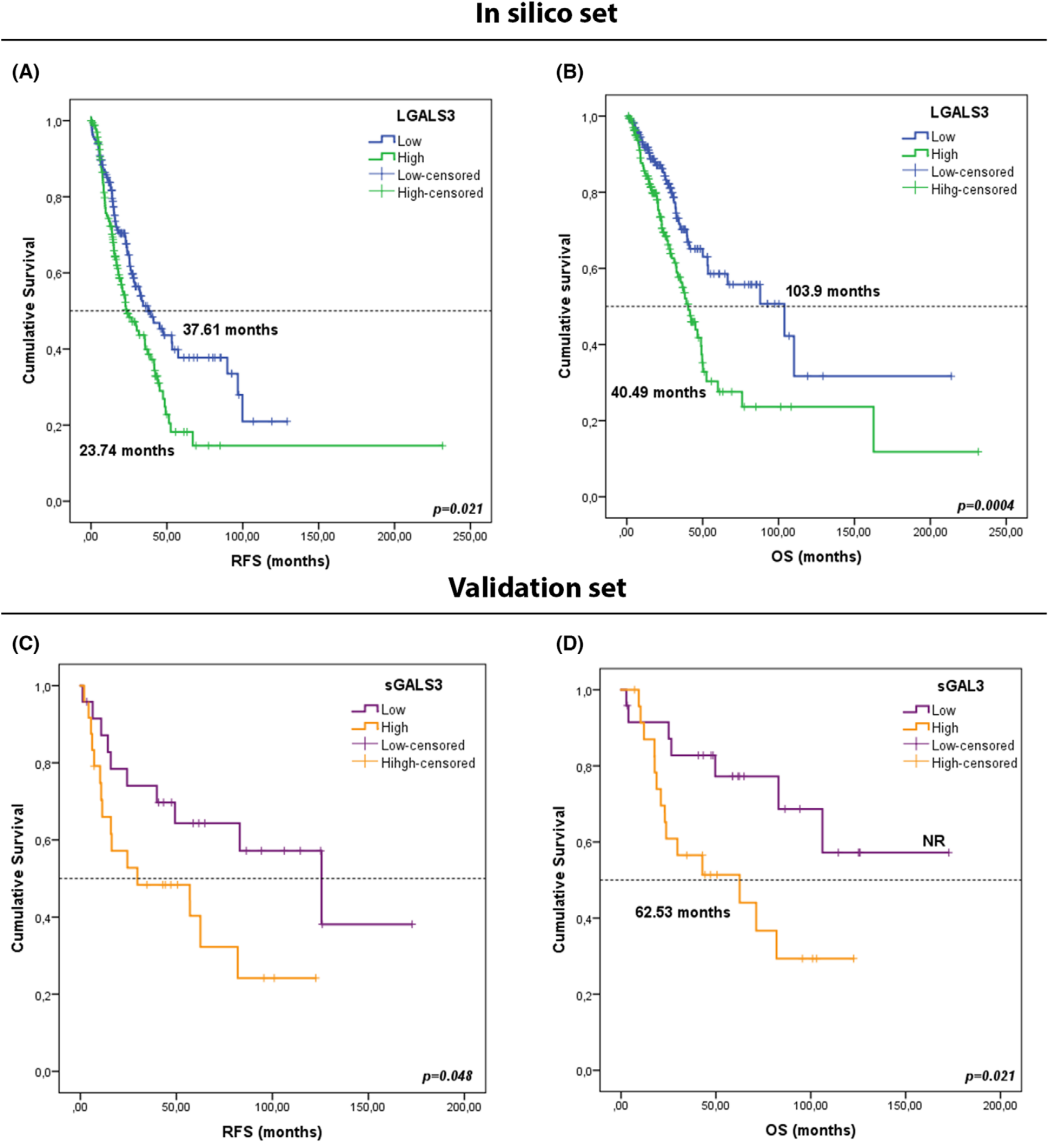
Kaplan–Meier survival curves according to *LGALS3* from TCGA *in silico* set. (A, B) and sGAL‐3 concentrations before surgery in validation set (C, D). (A) RFS stratified in high (*n* = 169) versus low LGALS3 concentrations (*n* = 169). (B) OS stratified in high (*n* = 169) versus low (*n* = 169) LGALS3 concentrations. The groups were divided as low and high according to its median. Green lines represent patients with high levels of expression, whereas blue lines represent patients with low levels of expression. (C) RFS stratified in high (*n* = 24) versus low sGAL‐3 levels (*n* = 24). (D) OS stratified in high (*n* = 24) versus low (*n* = 24) sGAL‐3 levels. The groups were divided as low and high according to its median. Orange lines represent patients with high levels of sGAL‐3 (> 9125.73 pg·mL^−1^), whereas purple lines represent patients with low levels of sGAL‐3 (≤ 9125.73 pg·mL^−1^). *P*‐values were obtained using the log‐rank test.

Data from TCGA for LUAD and LUSC patients were used to associate GAL‐3 with prognosis. Characteristics of 338 patients from TCGA (*in silico* set) from LUAD cohort are presented in Table 2. Patients with post‐surgical complications were excluded from the survival analysis, and only those patients who had more than 1 month of follow‐up were included (*n* = 338). In TCGA cohort, Cox regression and Kaplan–Meier analyses indicated that patients with high levels of LGALS3 presented worse RFS (23.74 months vs 37.61 months, *P* = 0.021) and OS (40.49 months vs 103.9 months, *P* = 0.0004) than those patients with low levels of *LGALS3* (Table 3 and Fig. 9). Other significant association between survival and clinicopathological variables were found (Table 3 and Fig. S9). Characteristics of 313 patients from TCGA (*in silico* set) from LUSC cohort are shown in Table S3. No significance results were found for LUSC cohort.

**Table 2:** Clinicopathological characteristics of the LUAD patients included in the study. *n*, sample size; NS, non‐specified.

	*In silico* cohort		Plasma validation set	
	*n* = 338	%	*n* = 48	%
Age at surgery (median, range)	67 [IQR 38–88]		65.5 [IQR 42–84]	
Gender				
Male	161	47.6	28	58.3
Female	177	52.4	20	41.7
Stage				
I	195	57.7	23	47.9
II	86	25.4	15	31.3
IIIA	57	16.9	10	20.8
PS				
0	NS	NS	39	81.3
1			9	18.8
Smoking status				
Current	81	24	21	43.8
Former	175	51.8	16	33.3
Never	82	24.3	11	22.9
*EGFR*				
Mutated	NS	NS	8	16.3
WildType			39	79.6
NS			2	4.1
*KRAS*				
Mutated	NS	NS	11	22.4
WildType			28	57.1
NS			10	20.4
Relapse				
No	196	58.0	26	54.2
Yes	121	35.8	22	45.8
NS	21	6.2		
Exitus				
No	226	66.9	27	56.3
Yes	112	33.1	21	43.8

**Table 3:** Results from the univariate Cox regression model for OS and RFS on LUAD *in silico* set. LN, lymph node. *P‐value significative.

	*In silico* set (*n* = 338)					
	RFS			OS		
	HR	95% CI	*P*‐value	HR	95% CI	*P*‐value
LGALS3 High vs Low	1.551	1.136–2.117	0.003*	1.968	1.341–2.888	0.0001*
Gender Male vs female	0.879	0.644–1.191	0.397	0.901	0.621–1.306	0.582
Age > 65 vs ≤ 65	1.291	0.933–1.786	0.123	1.308	0.881–1.941	0.183
TNM staging III vs II vs I	1.465	1.213–1.771	<0.0001*	1.560	1.243–1.958	<0.0001*
Tumor size T3/T4 vs T2 vs T1	1.207	1.097–1.328	<0.0001*	1.172	1.041–1.320	0.009*
LN involvement Yes vs no	1.722	1.260–2.354	0.001	2.116	1.455–3.079	<0.0001*
Smoking status Former/current vs never	0.831	0.590–1.172	0.291	0.754	0.501–1.133	0.174

To evaluate the potential use of *LGALS3* as an independent prognostic biomarker, a multivariate Cox regression analysis was performed including all the clinicopathological variables (gender, age, tumor node metastasis (TNM) staging, smoking status, and *LGALS3*). Results obtained from this multivariate analysis indicated that TNM staging and *LGALS3* were independently associated with survival (Table 4).

**Table 4:** Results from the multivariate Cox regression model for OS and RFS on LUAD *in silico* set.

	*In silico* set (*n* = 338)					
	RFS			OS		
	HR	95% CI	*P*‐value	HR	95% CI	*P*‐value
*LGALS3* (High vs Low)	1.908	1.294–2.814	0.001	1.513	1.092–2.096	0.013
Tumor size T3/T4 vs T2 vs T1	1.568	1.249–1.968	<0.0001	1.451	1.193–1.763	<0.0001

An independent cohort of plasma from patients with resected lung cancer from HGUV was used for validation of sGAL‐3 prognosis. Clinicopathological characteristics of LUAD cohort are summarized in Table 2 (validation set). In the same way, clinicopathological characteristics of LUSC cohort are summarized in Table S3. In LUAD patients, with a median duration of follow‐up of 48 months (IQR: 2.80–172.70 months), 21 patients were deceased at the time of cut‐off due to relapse (43.8%). Those with high levels of sGAL‐3 presented worse OS and in the same way, levels of sGAL‐3 tended to be higher in patients with worse PFS with Cox regression and Kaplan–Meier (Fig. 9 and Table 5). Other significant association between survival and clinicopathological variables were found (see Table 5 and Fig. S10). No significance results were found for LUSC cohort.

**Table 5:** Results from the univariate Cox regression model for OS and RFS of LUAD validation Set. LN, lymph node. *P‐value significative.

	Validation set (*N* = 48)					
	RFS			OS		
	HR	95% CI	*P*‐value	HR	95% CI	*P*‐value
sGAL‐3 High vs Low	2.269	0.985–5.230	0.054	2.844	1.127–7.176	0.027*
Gender Male vs female	2.802	1.117–7.031	0.028*	2.870	1.049–7.848	0.040*
Age > 65 vs ≤ 65	0.738	0.327–1.662	0.163	1.071	0.453–2.529	0.879
TNM staging III vs II vs I	1.762	1.086–2.857	0.022*	1.653	0.977–2.797	0.061
Tumor size T3/T4 vs T2 vs T1	1.792	0.976–3.293	0.060	1.506	0.805–2.815	0.200
PS 0 vs 1	3.354	1.352–8.321	0.009*	2.803	1.072–7.331	0.036*
LN involvement Yes vs no	2.023	0.878–4.661	0.098	1.556	0.626–3.866	0.341
Smoking status Former/current vs never	3.311	0.981–11.17	0.054	1.803	0.599–5.427	0.294

Multivariate Cox regression analysis including all clinicopathological variables (gender, age, TNM staging, *KRAS* mutation status, *EGFR* mutation status, smoking status, and *LGALS3*) on RFS and OS confirmed that sGAL‐3 could be a prognosis independent biomarker with a hazard ratio (HR) at 2.862 (95% CI 1.057–7.753; *P* = 0.039) and 3.580 (95% CI 1.185–10.81; *P* = 0.024), respectively. Gender for OS and performance status (PS) for RFS were also confirmed as prognosis independent factors (Table 6).

**Table 6:** Results from the multivariate Cox regression model for RFS and OS on LUAD validation set.

	Validation set (*N* = 48)					
	RFS			OS		
	HR	95% CI	*P*‐value	HR	95% CI	*P*‐value
*LGALS3* High vs Low	2.862	1.057–7.753	0.039	3.580	1.185–10.81	0.024
Gender Male vs female	–	–	–	3.238	1.043–10.05	0.042
PS 0 vs 1	3.139	1.116–8.829	0.030	–	–	–

### 3.8 Analysis of prognostic and predictive value of sGal‐3 in NSCLC advanced‐stage cohort

Following, we analyzed the possible predictive and prognostic value of sGal‐3 in NSCLC advanced‐stage cohort. Characteristic of the 34 LUAD patients are presented in Table 7. Patients were mostly male (79.4%), current or former smokers (94.1%) and with IV stage disease at diagnosis (82.4%). All patients were tested through Next Generation Sequencing panel Oncomine Precision Assay for genomic profiling. None of the patients harbored targetable drivers approved by European Medicines Agency. Pembrolizumab was given as first‐line in 100% of cases with PDL‐1 ≥ 50%, and patients had good PS (0–1) at pembrolizumab initiation in 85.5% of cases. The ORR with pembrolizumab in the global population was 44.1% (*n* = 15), 55.9% (*n* = 19) had DCB (3CR, 10 PR and 6 SD) under pembrolizumab whereas the remaining 44.11% (*n* = 15) had non‐DCB. With a median duration of follow‐up of 20.01 months (IQR: 6.15–31.83 months), 23 patients were deceased at the time of cut‐off due to tumor progression (67.7%). The median pembrolizumab PFS was 6.30 (IQR: 2.59–18.67). At PRE and FR (2 months of treatment), median sGAL‐3 concentrations were 10 150.88 pg·mL^−1^ (IQR: 7985.53–13 082.43) and 10 126.5750 pg·mL^−1^ (IQR: 8150.89–140 89.95), respectively. Characteristics of the 13 LUSC patients are presented in Table S4.

**Table 7:** Patient's characteristics of advanced‐stage LUAD cohort. *n*, sample size.

Patient characteristics	LUAD advanced cohort	
	*n* = 34	%
Age at surgery (median, range)	67 [IQR 52–89]	
Gender		
Male	27	79.4
Female	7	20.6
Stage		
III	6	17.6
IVA	11	32.4
IVB	17	50
PS		
0–1	29	85.3
2	4	11.8
Smoking status		
Current	25	73.5
Former	7	20.6
Never	2	5.9
PD‐L1 TPS^a^		
100%	2	5.9
95%	3	8.8
90%	8	23.5
80%	5	14.7
70%	8	23.5
60%	8	23.5
Progression		
Yes	24	70.6
No	10	29.4
Exitus		
Yes	23	67.6
No	11	32.4

^a^ PD‐L1 expression was assessed by TPS.

#### 3.8.1 ORR, clinical benefit and survival in advanced‐stage LUAD

**Fig. 10:**
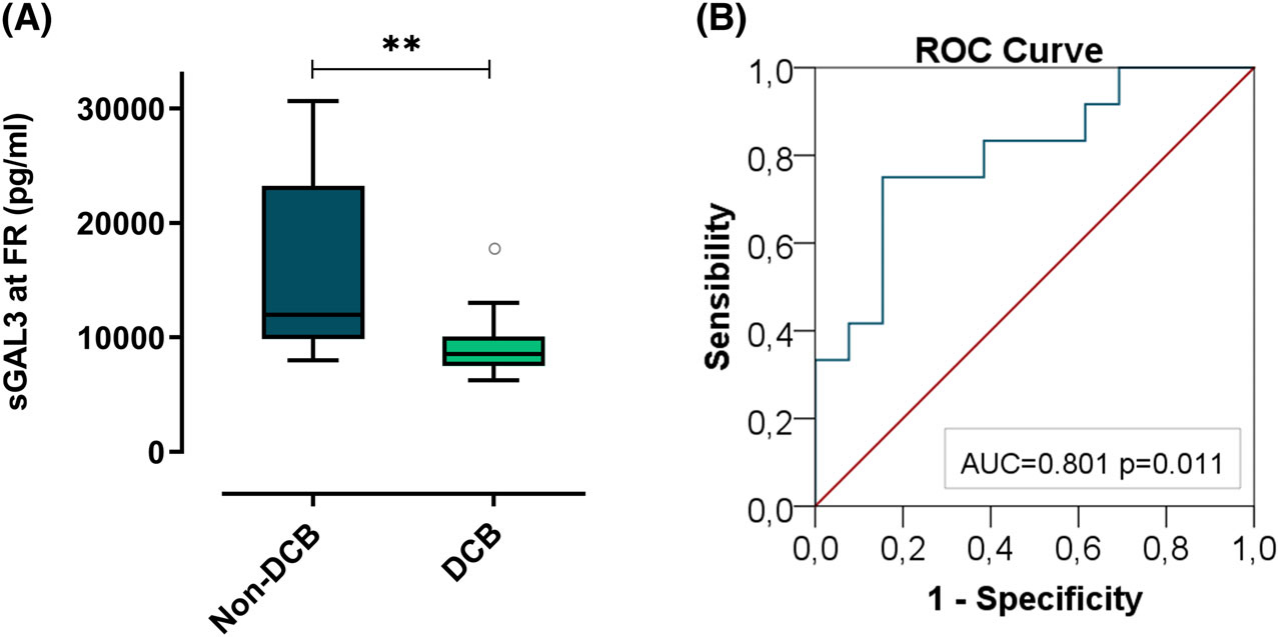
Analysis of predictive value in terms of DCB of sGal‐3 in LUAD advanced cohort. (A) sGAL‐3 concentrations at FR evaluation in patients with DCB response (*n* = 13) and patients without DCB response (*n* = 12). Data are the median values and bars represent minimum and maximum values. *P*‐values were obtained using the Mann–Whitney test. (B) Receiving operating characteristics (ROC) curve of sGAL‐3 for discriminating between patients with DCB and patients without DCB representing the area under the ROC curve (AUC). Statistical analysis was carried out with the ROC analysis. Significance values were **P ≤ 0.01. ° Outliers.

In LUAD patients, in terms of DCB, at FR, sGAL‐3 concentrations were significantly higher in patients without clinical benefit with a median value of 11 972.50 pg·mL^−1^ (IQR, 8040.25–23 224.5975) compared to 8815.97 pg·mL^−1^ (IQR, 7540.93–10 126.5750) in patients with clinical benefit (*P* = 0.010) (Fig. 10A). To determine sGAL‐3 levels predictive of patients with DCB, we performed a ROC curve analysis, which determined a cut‐off concentration of 10 438.115 pg·mL^−1^ associated with a sensitivity of 75%, a specificity of 84.6%, a PPV of 81.8% and NPV of 78.6% to predict durable clinical response to pembrolizumab at FR with an AUC of 0.801 (*P* = 0.011) (Fig. 10B). Using this cut‐off, we determined that patients with high sGALS3 concentrations (*n* = 11) had an DCB rate of 18.2%, whereas patients who had low sGAL‐3 concentrations (*n* = 14) had a DCB rate of 78.6% (*P* = 0.003). However, at PRE, median sGAL‐3 concentrations tended to be higher in patients with clinical benefit with a median value of 11 208.02 pg·mL^−1^ (IQR, 8014.89–14 623.86) compared to 9185.27 pg·mL^−1^ (IQR, 7485.67–11 330.53) in patients with clinical benefit (*P* = 0.157). The ORR analysis elucidates no statistical difference in sGAL‐3 concentrations measured at PRE and at FR in patients who were responders compared to non‐responders to pembrolizumab (Fig. S11). No significance results were found for LUSC advanced cohort (data not shown).

**Fig. 11:**
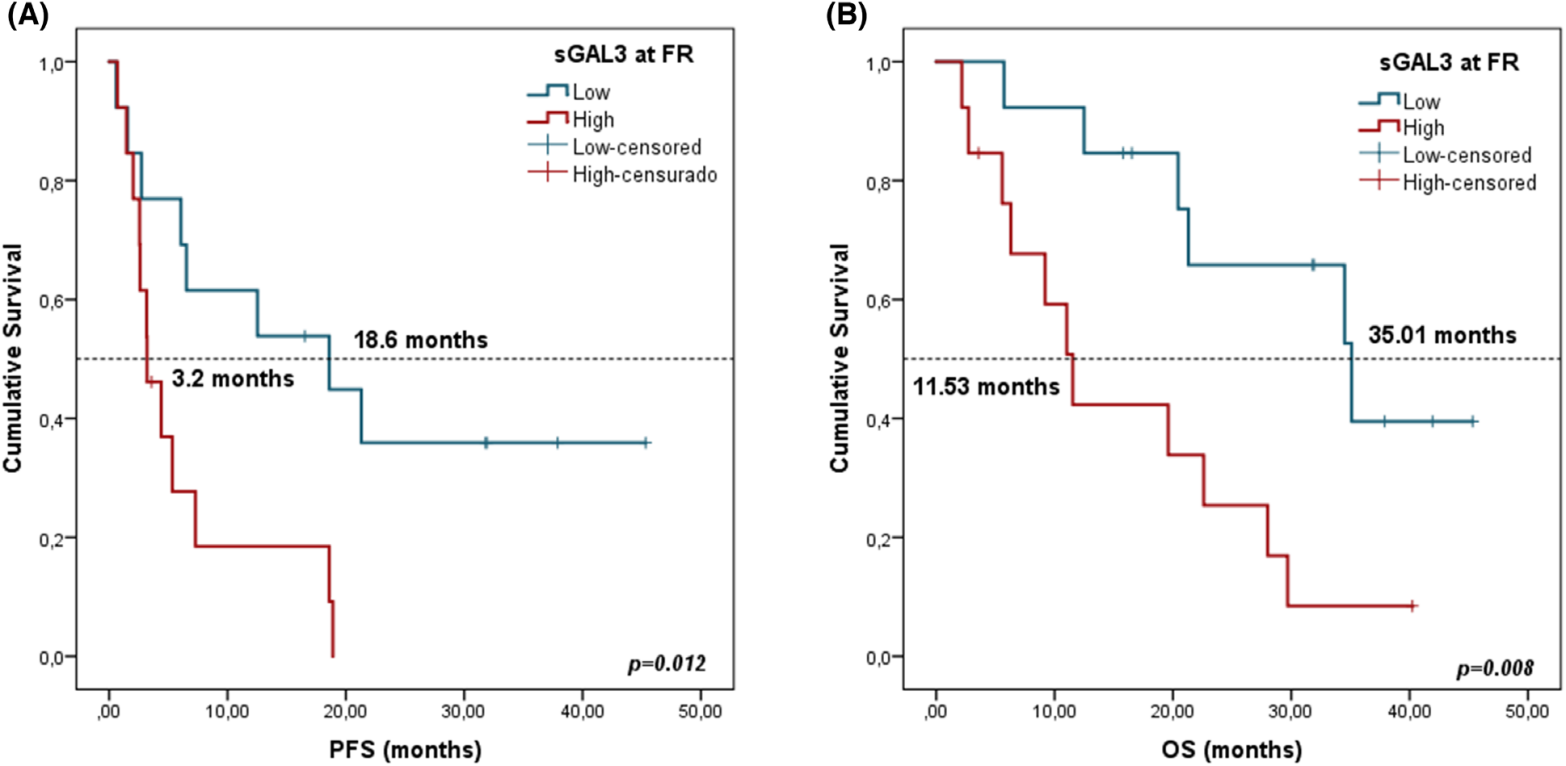
Kaplan–Meier survival curves according to sGAL‐3 concentrations at FR in LUAD advanced cohort. (A) PFS stratified in high (*n* = 13) versus low sGAL‐3 levels (*n* = 13). (B) OS stratified in high (*n* = 13) versus low sGAL‐3 levels (*n* = 13). The groups were divided as low and high according to median value. Red lines represent patients with high levels of expression, whereas blue lines represent patients with low levels of expression. *P*‐values were obtained using the log‐rank test.

Patients with high sGAL‐3 concentrations (≥ median) at FR were associated in cox regression analysis with worse PFS and OS in LUAD patients (HR: 3.215, 95% CI: 1.226–8.431, log‐rank *P* = 0.018 and HR: 3.639, 95% CI: 1.317–10.056, log‐rank *P* = 0.013, respectively). Kaplan–Meier analysis also showed a significant association of sGAL‐3 at FR with patient prognosis. Patients with high sGAL‐3 levels (> median) had shorter PFS (3.20 vs 18.6 months, *P* = 0.012) and OS (11.53 vs 35.1 months, *P* = 0.008) (Fig. 11). In contrast, median sGAL‐3 concentrations at PRE tent to be higher in patients with worse PFS but there was no statistical difference in OS (Fig. S12). Other significant associations between survival and clinicopathological variables were found in Fig. S13. No significance results were found for LUSC advanced cohort (data not shown).

Multivariate analysis including all clinicopathological variables (gender, age, TNM staging, smoking status, and sGAL‐3) on PFS and OS confirmed that sGAL‐3 could be a prognosis independent biomarker with a HR at 3.215 (95% CI 1.226–8.431; *P* = 0.015) and 3.639 (95% CI 1.317–10.056; *P* = 0.013), respectively.

## 4 Discussion

Despite the recent advance in the treatment of NSCLC, the prognosis remains very poor due to the delay in the detection of the disease. In the last decade, ICBs have considerably improved the treatment of advanced NSCLC producing powerful antitumor effects, however, the immune therapy prediction remains poor or limited. In this context, TME, a complex ecosystem which comprises interactions between cancer cells including CSCs, immune cells, stromal cells such as fibroblast and extracellular matrix elements, plays an important role in promoting immune evasion and suppression [30].

In the last years, preclinical studies have been focused on understanding the mechanisms involved in immune evasion and immunosuppression in TME. Cancer cells achieve immunosuppression through several mechanisms: for instance, recruit different cellular types such as cancer‐associated fibroblast, tumor‐associated macrophages or regulatory T cells (T_REGS_); they are able to activate inhibitory pathways in immune cells, impair antigen presentation, and tumor cells can also secrete immunosuppressive and pro‐apoptotic cytokines and chemokines [31, 32, 33]. The evaluation of immune molecules' expression on tumor cells could provide the knowledge to comprehend better tumor immune evasion mechanisms. For this purpose, some studies have been focused on using tumorspheres, a 3D model system with outstanding applications for *in vitro* studies [34, 35]. Recently, Bertolini et al. reports that spheroid from cell lines are enriched in metastasis initiating cells with immunosuppressive potential [36]. In this work we proposed tumorspheres as a model to study the role of an immunoregulatory protein, glycoprotein GAL‐3 in lung cancer. What is more, we go one step further and in order to mimic more accurately the TME, we used a co‐culture of tumorspheres and fibroblast, one of TME components, revealing the importance of GAL‐3 as a molecule expressed and secreted in TME modulating immunosuppression through T_REGS_. Our results confirm that lung tumorspheres express significantly more GALS3 than adherent cells, additionally more significant levels of sGAL‐3 compering with monolayer cells. Ling‐Yeng Chung et al. [37] studied the expression of GAL‐3 from NSCLC commercial cell lines (A549 and H1299) and revealed that spheroids express relatively high levels of this molecule over serial passages compared to monolayers cells acting as a cofactor by interacting with β‐catenin to augment the transcriptional activities of stemness‐related genes. Notably, we have analyzed the expression of GAL‐3 obtaining the same results not only on a large number of lung tumorspheres from cell lines moreover in primary patient‐derived cell cultures from our hospital, which are a suitable and translational platform as described by some other authors [38, 39, 40]. GAL‐3 exerts different biological effects depending on its cellular localization through specific interaction with intra‐ and extracellular proteins affecting numerous biological processes such as neoplastic transformation and metastasis [41, 42, 43]. In concordance, our results revealed that GAL‐3 in our NSCLC cells could be found in the cytoplasm, within the nucleus, on the cell surface and in the extracellular compartment depending on the cell line. GAL‐3‐binding protein (LGALS3BP) is a hyperglucosylated protein that acts as a ligand for GAL‐3 that can induce the survival of cancel cells during the metastatic process [44]. Because of its relationship with GAL‐3, we decided to study its expression in cell cultures and its correlation with *LGALS3*. We have demonstrated that LUAD tumorspheres expressed higher levels of *LGALS3BP* than adherent cells and exist a positive correlation with expression of *LGALS3* in LUAD cell cultures. A previous study has reported that in the microenvironment of human neuroblastoma, GAL‐3BP interacts with GAL‐3 in bone marrow mesenchymal stem cells and induces transcriptional upregulation of IL‐6, via the Gal‐3BP/Gal‐3/Ras/MEK/ERK signaling pathway [45, 46]. In lung cancer, no previous studies have been reported about their correlation. Our results suggest that these two genes may cooperatively participate in the pathological process of cancer. Future studies should be performed in order to elucidate the mechanisms involved.

Extracellular vesicles are a subset of small membrane‐bound structures secreted by different cells. EVs are an important part of TME acting as effective signaling molecules between cancer cells and the surrounding cells [47]. We had previously performed an exhaustive characterization of NSCLC EVs revealing that EVs cargo can reflect the molecular signatures and their capacity to be used as a tool for diagnosis and prognosis [48]. In view of potential role of secreted Gal‐3 as an immunomodulator molecule, we analyzed EVs‐associated Gal‐3 in our cohort of cell cultures. We found that *LGALS3* presented significantly higher expression in LUAD secreted EVs derived from tumorspheres than LUAD secreted‐ EVs derived from adherent cells. Moreover, the expression of GAL‐3 in LUAD cell‐derived EVs was correlated with the expression and secretion of GAL‐3 in LUAD cell cultures. Previously, GAL‐3 has been found in EVs from bladder cancer and colon cancer but no reports were found in EVs from lung cancer [49, 50]. Our results reveal that not only GAL‐3 from tumor cells but also a vesicular form of Gal3 could act as an external factor such as within EVs to help cells in the microenvironment communicate with each other. Further proteomics and plasma EVs studies should be performed to deep further into this research pathway.

Focusing on immune TME, some studies revealed that extracellular sGAL‐3 secreted by tumor cells restricts TCR movement, induces T‐cell apoptosis and potentiate TCR downregulation [51, 52, 53, 54]. However, the specific effect of sGAL‐3 on T_REGS_ in TME has been poorly studied. We have used the CM from the co‐culture between lung tumorspheres from PC435 and a fibroblast cell line to examine the effect on T_REGS_ and the role that sGAL‐3 may be playing on it. CM from co‐culture (PC435 and fibroblast cell line) increased the T_REGS_ population and the blocking of sGAL‐3 through an antibody anti‐GAL‐3 recues this phenotype. Overall, our study revealed that some components of TME in lung cancer such as tumor cells with stem‐like properties and fibroblast could be favors an immunosuppressive microenvironment possibly recruiting T_REGS_ through sGAL‐3.

Carrying on this path, we aimed to further explore the relationship between GAL‐3 and different lymphocyte populations, including T_REGS_, and determined if there is a correlation between them to further support our prior findings. First, in a cohort of early‐stage LUAD patients from HGUV we found that those patients with high FOXP3+ infiltration in tumor had high expression of *LGALS3* in tumor. Moreover, we found also a positive correlation between *FOXP3* and *LGALS3* at gene expression level. Secondly, CIBERSORTx tool with the TCGA database was used to validate the relationship between GAL‐3 and different cellular subtypes, including T_REGS_. We identified four clusters, where the one characterized by high levels of T_REGS_ also had the highest percentage of patients with high levels of GAL‐3 expression. With these experiments we are observing that depending on the high or low expression of GAL‐3, patients have more or fewer T_REGS_. As GAL‐3 regulates immune cell function to promote tumor‐driven immunosuppression [55] based on our results, we can hypothesize that the lung tumor cells may attract the population of T_REGS_ as a mechanism of tumor immune evasion by GAL‐3.

The prognosis of NSCLC remains poor and heterogeneous and new biomarkers are needed. Our previous study described that the proportion of T helper and cytotoxic cells versus T_REGS_ in different locations of the TME have opposite prognostic impacts in resected NSCLC [20]. Furthermore, we have also revealed an immune‐checkpoint score (PD1 and CTLA4) with relevant prognostic for a better characterization of early‐stage NSCLC [56]. In accordance with our prior analyses, we would like to verify the possible prognosis role of GAL‐3 on NSCLC patients, focusing on early‐stage due to the tumor resection for these patients offers the best hope of cure, however, recurrences rates post‐surgery remaining extremely increased [57]. First, for this purpose, RNAseq data from a tumor tissue from a TCGA cohort of 331 early NSCLC patients was analyzed. Our results have confirmed that the expression of GAL‐3 on LUAD patients from TCGA database is an independent prognostic biomarker for RFS and OS. Despite this, some limitations such as partial clinical outcome information which might lead to some uncertainties in the results. Nevertheless, TCGA database is public, provide massive information and allows carry out *in silico* analysis such performed previously in our laboratory [22, 48].

Nowadays, studies have been focused on looking for new minimal invasive methodologies such as soluble immune mediators analysis on plasma samples. Many circulating proteins have been investigated as prognostic biomarkers in the early lung cancer management; one of the most investigated proteins have been CEA and CYFRA 21‐1 [58]. However, their use in the routine clinical practice has been limited by the lack of both independent validation and reproducibility. Therefore, there is a necessity of new reliable biomarkers for early‐stage NSCLC, we propose sGAL‐3 as a new potential prognostic and predictive biomarker in lung cancer. Tumors cells are able to release sGAL‐3 to the media confirmed in the *in vitro* experiments. Generally, soluble ligands and receptors can be produced by mRNA expression or by the cleavage of membrane‐bound proteins. Specifically, GAL‐3 can be cleaved by matrix metalloproteinases and found free on plasma [59]. Blood levels of Gal‐3 were found to be significantly higher in cancer patients than in controls [60]. In consequence, our results revealed that the secretion of sGAL‐3 on resected LUAD patients' plasma (in a validation set) is an independent prognostic biomarker for RFS and OS. In accordance with our results, previous studies in early NSCLC reported that GAL‐3 expression on tumor cells has been reported to be associated with progression, poor prognosis and recurrence after radical resection on tissue samples [61]. Using non‐invasive methodologies, Yoko Kataoka et al. [62]. were analyzed the value of sGAL‐3 on 42 early NSCLC sera, but no prognostic role has been found. Luminex® MAP technology instead of an enzyme‐linked immunosorbent assay conventional, allow higher throughput, smaller sample volume, and higher sensitivity [63]. Moreover, this technology facilitates the evaluation of simultaneous multiple mediators. As far as we know, this is the first study elucidating the prognostic value of sGAL‐3 on early LUAD patients underwent surgery. One of the robustness of our study is that we employed a validation cohort from HGUV with a relatively long follow‐up (median of 48 months, IQR, 2.8–172 months).

Despite the big efforts to look for new prognostic and predictive biomarker to immunotherapy in advanced NSCLC, data remain very poor and heterogeneous [64]. The expression level of PD‐L1 on tumor immune cells has emerged as the first reliable predictive biomarker for sensitivity to ICB in advanced NSCLC patients treated with immunotherapy [65]. However, PD‐L1 expression in tissue as a predictive biomarker has limitations: range of different antibodies used in clinical trials, different positive thresholds, heterogeneity in PD‐L1 staining in the tumor, insufficient tumor tissue, among others [66]. Plasmatic biomarkers have many advantages of being repeatable and easily accessible. There are some studies about new plasmatic biomarkers as putative prognostic and predictive biomarkers associated with immune checkpoint inhibitors efficacy in NSCLC. For instance, Okuma et al. [67] revealed that baseline plasma sPD‐L1 levels could represent a novel predictive biomarker of nivolumab therapy against NSCLC. Moreover, other plasmatic biomarkers such as sGranB were associated with the response to nivolumab and also together with sPD‐L1 were associated with the PFS and OS [68]. However, studies with plasmatic biomarkers about predict prognosis and tumor response to pembrolizumab remain currently sparse. In our study the prognosis and predictive value of sGAL‐3 in a cohort of advanced LUAD patients treated with pembrolizumab was evaluated. Our results demonstrate that sGAL‐3 levels were significantly higher in patients without clinical benefit and worse PFS and OS. These clinical results are supported by a strong biological basis in which GAL‐3 have been shown to attenuate the effect of immune cells contributing to tumor cell evasion [43]. Our results are consistent with a recent study that proposed a GAL‐3 signature for the selection of candidates for immunotherapy analyzing 34 NSCLC patients [69]. In this study, those patients with high GAL‐3 tumor expression before treatment showed an early and dramatic progression after three cycles of treatment, and patients with negative or low/intermediate expression of GAL‐3 showed an early and durable objective responsiveness [69]. Conversely to Capalbo's study, we analyzed baseline as well as FR samples, confirming the predictive and prognosis value of sGAL‐3 in LUAD patients using a non‐invasive methodology. Our results contribute to use a fast and high‐sensitivity methodology that could be implemented for evaluating the secretion of sGAL‐3 in plasma samples, predicting tumor response in patients treated with immunotherapy. In accordance with our results, Jung Sum Kim et al. also revealed that high blood Gal‐3 levels at PRE (serum or plasma depending on the availability) may predict worse OS in patients with advanced NSCLC treated with ICBs. In our study, in addition to employing PRE samples, we also evaluated first‐response assessment samples demonstrating on them not only the prognostic but also the predictive impact of efficacy of pembrolizumab in LUAD patients. Moreover, contrary to these authors that used heterogeneous samples (different types of ICB, line of treatments, and source) we used homogeneous samples (plasma samples from patients treated in first‐line with pembrolizumab) [70].

Our study suggests that plasma sGAL‐3 levels will help to select suitable patients for pembrolizumab treatment in advanced NSCLC, probably by excluding those with high plasma levels of sGAL‐3. In contrast, the addition of a Gal‐3 inhibitor in patients with high Gal‐3 levels may be a suitable treatment to improve outcomes [71]. To date, several GAL‐3 inhibitors are under clinical investigation both alone and in combination with check‐point inhibitors in different cancer settings. GAL‐3 has not been reported as marker for treatment efficacy during immunotherapy in NSCLC or other cancers so far. However, a GAL‐3 inhibitor (GR‐MD‐02), in combination with pembrolizumab or an anti‐CTLA4 inhibitor, is being currently evaluated for the treatment of patients with metastatic NSCLC, melanoma and squamous cell head and neck cancer patients (NCT02575404) highlighting that GAL‐3 could be part of a panel of biomarkers that predicts the outcome for immunotherapy in NSCLC [72]. Furthermore, more recently, a new clinical trial has been opened to test the safety and efficacy of other Gal‐3 inhibitor (GB12211) in combination with atezolizumab in patients with advanced NSCLC (NTC05240131) remarking the relevance of including Gal‐3 as predictive biomarker for ICBs.

Although our study supports that sGAL‐3 could be used a prognostic and predictive biomarker for advanced LUAD patients, some limitations have should be considered. Our study includes a small number of patients, and the results need to be confirmed in a large cohort of patients with a larger follow‐up. If these results will be confirmed, a better selection of responders' candidates for immunotherapy using sGAL‐3 could be feasible, preventing ineffective treatments. As far as we know, this is the first report to address the independent prognostic role and predictive tumor response of sGAL‐3 found on advanced LUAD patients' plasma treated with pembrolizumab in the first line with a non‐invasive methodology.

## 5 Conclusions

In summary, we present an *in vitro* and translational robust study of GAL‐3 in NSCLC. Our *in vitro* study demonstrate that NSCLC tumor cells express and secret GAL‐3 acting as a regulator of immune microenvironment through T_REG_. Focusing on the translational research studies, sGAL‐3 might be applied as a novel independent biomarker to predict clinical outcomes for surgery in early LUAD patients. Furthermore, sGAL‐3 is useful, not only to assess the prognosis as an independent biomarker in early stages, but also to predict the clinical outcomes for pembrolizumab in advanced LUAD patients. Prospective validation of this biomarker in a larger study should be performed to confirm these findings.

## Conflict of interest

The authors declare no conflict of interest.

## Authors contributions

Conceptualization, EJ‐L, SC‐F, CC; methodology, ST‐M, EE, AM‐M, ED‐S, AB; software, ST‐M, AM‐M, AH‐P; validation and formal analysis, ST‐M, GB, AM‐M, AH‐P, RG, AB; investigation ST‐M, AM‐M, ED‐S, GB, LR, SC‐F, EJ‐L; resources, RG, AB, LR; data curation, ST‐M, AM‐M, SC‐F, GB, AH‐P, RG; writing‐original draft preparation, ST‐M, SC‐F; writing‐review and editing, ST‐M, SC‐F, EJ‐L, AB, RG; visualization and supervision, EJ‐L, SC‐F, CC; project administration and funding acquisition, EJ‐L, SC‐F, CC. All authors have read and agreed to the published version of the manuscript.

### Peer review

The peer review history for this article is available at https://www.webofscience.com/api/gateway/wos/peer‐review/10.1002/1878‐0261.13505.

## Supporting information


**Fig. S1.** Dataset from CIBERSORTx.
**Fig. S2.** Heatmap of different cellular subtypes representing absolute cell fraction of different cellular subtypes.
**Fig. S3.** Representative images of the primary patient‐derived cancer cells and cell lines grown under adherent conditions and suspension conditions.
**Fig. S4.** Transcription levels of *LGALS3* in tumorspheres versus adherent‐culture in different LUSC primary cultures and cell lines.
**Fig. S5.** Transcription levels of *LGALS3BP* in tumorspheres versus adherent‐culture in different LUAD primary cultures and cell lines.
**Fig. S6.** Original and complete immunoblots for B‐actin and Galectin‐3.
**Fig. S7.** Expression of *LGALS3* as protein level in LUSC cells.
**Fig. S8.** Representative immunofluorescence images of Gal‐3 in tumorspheres and adherent‐cultured cells from ADC patients.
**Fig. S9.** Kaplan–Meier survival curves according to clinicopathological variables from TCGA *in silico* set.
**Fig. S10.** Kaplan–Meier survival curves according to clinicopathological variables in validation set.
**Fig. S11.** Analysis of predictive value in terms of overall response rate (ORR) of sGal‐3 in LUAD advanced cohort.
**Fig. S12.** Kaplan–Meier survival curves according to sGAL‐3 concentrations at pretreatment (PRE).
**Fig. S13.** Kaplan–Meier survival curves according to clinicopathological variables in LUAD advanced cohort.
**Table S1.** TaqMan® Gene Expression Assay used in gene expression analyses.
**Table S2.** List of antibodies used for immunoblot (IB), immunofluorescence (IF), and flow cytometry (FC) analysis.
**Table S3.** Clinicopathological characteristics of the LUSC early patients included in the study.
**Table S4.** Patient's characteristics of advanced‐stage LUSC cohort.

## Data Availability

Data that support these findings are available from the corresponding authors upon reasonable request.
