# Supplementary material for: Soluble galectin‐3 as a microenvironment‐relevant immunoregulator with prognostic and predictive value in lung adenocarcinoma
Source: Mol Oncol. 2023 Nov 1;18(1):190–215. doi: 10.1002/1878-0261.13505 (PMC10766205; doi:10.1002/1878-0261.13505)
Supplement: Supplementary file 1 — Fig. S1. Dataset from CIBERSORTx. Fig. S2. Heatmap of different cellular subtypes representing absolute cell fraction of different cellular subtypes. Fig. S3. Representative images of the primary patient‐derived cancer cells and cell lines grown under adherent conditions and suspension conditions. Fig. S4. Transcription levels of LGALS3 in tumorspheres versus adherent‐culture in different LUSC primary cultures and cell lines. Fig. S5. Transcription levels of LGALS3BP in tumorspheres versus adherent‐culture in different LUAD primary cultures and cell lines. Fig. S6. Original and complete immunoblots for B‐actin and Galectin‐3. Fig. S7. Expression of LGALS3 as protein level in LUSC cells. Fig. S8. Representative immunofluorescence images of Gal‐3 in tumorspheres and adherent‐cultured cells from ADC patients. Fig. S9. Kaplan–Meier survival curves according to clinicopathological variables from TCGA in silico set. Fig. S10. Kaplan–Meier survival curves according to clinicopathological variables in validation set. Fig. S11. Analysis of predictive value in terms of overall response rate (ORR) of sGal‐3 in LUAD advanced cohort. Fig. S12. Kaplan–Meier survival curves according to sGAL‐3 concentrations at pretreatment (PRE). Fig. S13. Kaplan–Meier survival curves according to clinicopathological variables in LUAD advanced cohort. Table S1. TaqMan® Gene Expression Assay used in gene expression analyses. Table S2. List of antibodies used for immunoblot (IB), immunofluorescence (IF), and flow cytometry (FC) analysis. Table S3. Clinicopathological characteristics of the LUSC early patients included in the study. Table S4. Patient's characteristics of advanced‐stage LUSC cohort. [file MOL2-18-190-s001.pdf]

Table S1. TaqMan® Gene Expression Assay used in gene expression analyses.

Gene Symbol	Gene name	Assay ID
ACTB	Actin, Beta	Hs99999903_m1
GUSB	Glucuronidase, beta	Hs01558067_m1
CDKN1B	Cyclin-dependent kinase inhibitor 1B	Hs00153277_m1
LGALS3	Galectin-3	Hs00173587_m1

Table S2. List of antibodies used for immunoblot (IB), flow cytometry (FC) and immunofluorescence (IF) analysis.

Antibody	Dilution	Catalog nº	Supplier	Technique
Anti-Galectin 3 antibody (Clone A3A12)	1:2000	ab2785	Abcam	IB
B-Actin Anti-Mouse mAb (Clone AC-15)	1:10.000	A5441	Sigma-Aldrich	IB
Anti-Rabbit IgG (whole molecule)-Peroxidase	1:5000	Sc-2313	Santa Cruz Biotec.	IB
7AAD Viability Staining	1:10	00-6993-50	Thermofisher	FC
PE anti-Gal3 (clone M3/38)	1:200	125408	Biolegend	FC
APC Anti-Human CD25 (clone M-A251)	1:50	555434	BD Bioscience	FC
BV421 Anti-Human CD4 (clone SK3)	1:50	565997	BD Horizon	FC
BV510 Mouse Anti-Human CD3 (Clone HIT3a)	1:50	564713	BD Bioscience	FC
PE anti-Human FoxP3 (Clone 259D/C7)	1:50	560046	BD Bioscience	FC
Fixable Viability Stain 780	1:1000	565388	BD Horizon	FC
Alexa Fluor 488 Anti-Mouse IgG (H+L)	1:2000	A11001	Thermofisher	IF
Anti-Galectin 3 antibody [A3A12]	1:200	ab2785	Abcam	IF

CIBERSORTx Output Display

Input Sample	B cells naive	B cells memory	Plasma cells	T cells CD8	T cells CD4 naive	T cells CD4 memory resting	T cells CD4 memory activated	T cells follicular helper	T cells regulatory (Tregs)	T cells gamma delta	NK cells resting	NK cells activated	Monocytes	Macrophages M0	Macrophages M1	Macrophages M2	Dendritic cells resting	Dendritic cells activated	Mast cells resting	Mast cells activated	Eosinophils	Neutrophils	P-value	Correlation	RMSE
TCGA-05-4389-01A-01R-1206-07	0.085	0	0	0.505	0	0.655	0.079	0.22	0.231	0	0.123	0.027	0.046	0.306	0.24	0.896	0.203	0.083	0.227	0	0	0	0.000	0.714	0.755
TCGA-38-A44F-01A-11R-A24H-07	0.075	0.333	0.038	0.19	0	0.399	0	0.29	0.135	0	0	0.112	0.089	0.291	0.246	0.572	0.066	0.011	0.25	0	0	0.021	0.000	0.760	0.740
TCGA-44-2661-01A-01R-1107-07	0.209	0.061	0.152	0.21	0	0.701	0	0.349	0.111	0.057	0	0.054	0.157	0.069	0.272	1.101	0.2	0.095	0.364	0	0.009	0.026	0.000	0.827	0.711
TCGA-44-5643-01A-01R-1628-07	0.137	0.006	0.062	0.12	0	0.115	0.099	0.163	0.106	0	0.114	0	0.001	0.228	0.147	0.342	0.016	0.059	0.052	0	0	0	0.000	0.707	0.764
TCGA-44-6778-01A-11R-1858-07	0.188	0.074	0.067	0.169	0	0.746	0.063	0.234	0	0	0.028	0.043	0.02	0.599	0.253	0.945	0	0	0.083	0.01	0	0.034	0.000	0.744	0.730
TCGA-44-7659-01A-11R-2066-07	0.038	0.166	0.084	0.036	0	0.217	0	0.103	0.036	0	0.042	0.004	0.027	0.166	0.056	0.277	0.026	0.069	0.118	0	0	0	0.000	0.569	0.843
TCGA-44-8119-01A-11R-2241-07	0.254	0	0.37	0.074	0	0.383	0.027	0.176	0.215	0	0.145	0.031	0.055	1.11	0.2	0.83	0	0	0	0.012	0	0	0.000	0.740	0.726
TCGA-44-A4SS-01A-11R-A24X-07	0.084	0	0.01	0.165	0	0.517	0.061	0.246	0.085	0	0.219	0	0.051	0.234	0.284	0.675	0.007	0.018	0.101	0	0	0	0.000	0.616	0.805
TCGA-49-4490-01A-21R-1858-07	0.072	0	0.031	0.053	0	0.207	0	0.028	0.088	0	0.019	0	0.003	0.265	0.055	0.177	0	0.032	0.054	0	0	0	0.000	0.670	0.774
TCGA-49-4494-01A-01R-1206-07	0.18	0	0.403	0.376	0	0.071	0.292	0.453	0.571	0.132	0	0.158	0.088	0.336	0.55	0.728	0.429	0.109	0.313	0	0	0.05	0.040	0.165	1.011
TCGA-49-6742-01A-11R-1858-07	0.119	0	0.084	0.038	0	0.125	0	0.055	0.04	0	0.019	0.04	0.02	0.138	0.012	0.289	0	0.083	0.06	0	0	0.017	0.000	0.404	0.914
TCGA-50-5936-01A-11R-1628-07	0.034	0.027	0.045	0.009	0	0.259	0.002	0.067	0.058	0	0.068	0	0.004	0.64	0.038	0.287	0	0.043	0.037	0	0	0.012	0.000	0.757	0.691
TCGA-50-5941-01A-11R-1755-07	0.146	0.014	0.086	0.245	0	0.29	0.041	0.331	0.091	0	0.115	0	0.027	0.347	0.321	0.54	0.024	0.003	0.128	0	0	0.023	0.000	0.829	0.685
TCGA-50-5944-01A-11R-1755-07	0.037	0	0.03	0.075	0	0.175	0	0.068	0.034	0	0.022	0.017	0.05	0.244	0.047	0.419	0.012	0.026	0.306	0	0	0.009	0.000	0.601	0.815
TCGA-50-6594-01A-11R-1755-07	0.011	0	0.014	0	0	0.32	0.007	0.051	0.08	0	0.114	0	0.067	0.223	0	0.27	0.031	0.052	0.057	0	0	0	0.000	0.469	0.883
TCGA-50-6673-01A-11R-1949-07	0.173	0	0.243	0.024	0	0.214	0.015	0.148	0.059	0	0	0.013	0.05	0.315	0.052	0.515	0.004	0.094	0.03	0.021	0	0.012	0.000	0.518	0.862
TCGA-53-7813-01A-11R-2170-07	0.037	0	0.297	0.118	0	0.092	0.09	0.095	0.033	0	0.022	0	0.026	0.048	0.041	0.079	0	0.038	0	0.001	0.005	0	0.000	0.579	0.847
TCGA-55-6543-01A-11R-1755-07	0.09	0	0.116	0.019	0	0.253	0	0.056	0.023	0	0.048	0	0.034	0.528	0.074	0.56	0	0.08	0.06	0.026	0	0.064	0.000	0.635	0.787
TCGA-55-6980-01A-11R-1949-07	0.048	0	0.022	0.065	0	0.458	0	0.098	0.075	0	0.029	0.004	0.13	0.171	0.079	0.321	0.121	0.124	0.085	0	0	0	0.000	0.632	0.805
TCGA-55-6981-01A-11R-1949-07	0.114	0	0.045	0	0	0.183	0	0.115	0.034	0	0	0.019	0.034	0.245	0	0.229	0.035	0.087	0.074	0	0	0.002	0.000	0.707	0.762
TCGA-55-6985-01A-11R-1949-07	0.124	0	0.111	0.096	0	0.383	0.026	0.171	0.09	0	0.113	0.007	0.009	0.391	0.146	0.36	0	0.029	0.064	0.015	0	0.012	0.000	0.647	0.793
TCGA-55-7726-01A-11R-2170-07	0.009	0.002	0.124	0.032	0	0.189	0.014	0.027	0.011	0	0.007	0.005	0.036	0.169	0.033	0.257	0.12	0.067	0.041	0.03	0.006	0.024	0.000	0.624	0.811
TCGA-55-7727-01A-11R-2170-07	0.107	0.054	0.025	0.033	0	0.213	0.046	0.074	0.005	0.024	0.003	0	0	0.093	0.08	0.425	0	0.049	0.085	0	0	0.006	0.000	0.689	0.774
TCGA-55-8097-01A-11R-2241-07	0.094	0.121	0.061	0.111	0	0.366	0	0.279	0.056	0	0.01	0.036	0.047	0.094	0.075	0.577	0.112	0.097	0.362	0	0	0.003	0.000	0.665	0.796
TCGA-55-8510-01A-11R-2403-07	0.072	0.046	0.035	0.234	0	0.263	0.006	0.194	0.121	0	0.074	0.028	0.056	0.369	0.166	0.608	0.071	0.038	0.237	0	0	0.014	0.000	0.778	0.722
TCGA-62-A470-01A-11R-A24H-07	0.034	0	0.007	0.025	0	0.118	0	0.026	0.02	0	0.011	0.001	0	0.167	0.033	0.389	0	0.018	0.041	0	0	0.012	0.000	0.452	0.893
TCGA-75-7031-01A-11R-1949-07	0.013	0	0.063	0.111	0	0.179	0.017	0.086	0.032	0	0.051	0.023	0.008	0.152	0.078	0.287	0	0.059	0.044	0	0	0.006	0.000	0.683	0.772
TCGA-78-7163-01A-12R-2066-07	0.154	0	0.11	0.246	0	0.415	0	0.207	0.068	0	0.076	0.017	0.023	0.099	0.041	0.175	0.007	0.049	0.048	0	0	0.004	0.006	0.240	0.993
TCGA-78-7166-01A-12R-2066-07	0.015	0.143	0.163	0.134	0	0.27	0.007	0.166	0.093	0	0.14	0	0	0.433	0.144	0.147	0	0	0	0.025	0	0	0.000	0.448	0.893
TCGA-78-7633-01A-11R-2066-07	0.169	0	0.082	0.011	0	0.416	0	0.103	0	0	0.074	0.026	0.155	0.198	0.027	0.52	0	0.208	0.097	0	0	0	0.002	0.305	0.957
TCGA-86-8055-01A-11R-2241-07	0.075	0	0.202	0.003	0	0.478	0.024	0.07	0.053	0	0.118	0	0.033	0.549	0.139	0.561	0.037	0.097	0.081	0.017	0	0.041	0.000	0.776	0.716
TCGA-86-8668-01A-11R-2403-07	0.06	0.119	0.035	0.055	0	0.2	0.003	0.132	0.114	0	0.039	0.02	0.044	0.313	0.118	0.328	0	0	0.22	0	0	0	0.000	0.624	0.809
TCGA-86-8674-01A-21R-2403-07	0.065	0	0.133	0.052	0	0.093	0	0.191	0.037	0	0.059	0.008	0	0.406	0.014	0.121	0	0.087	0.004	0	0	0	0.000	0.447	0.894
TCGA-93-7347-01A-11R-2187-07	0.114	0.095	0.104	0.516	0	0.613	0.027	0.262	0.236	0	0.056	0	0.013	0.918	0.157	0.788	0.041	0.204	0.309	0	0	0	0.000	0.849	0.671
TCGA-93-7348-01A-21R-2039-07	0.125	0.032	0.262	0.05	0	0.294	0	0.175	0.033	0	0.023	0.002	0	0.505	0.1	0.202	0	0.035	0.092	0	0	0	0.000	0.678	0.780
TCGA-95-7567-01A-11R-2066-07	0.033	0.031	0.331	0.221	0	0.472	0.036	0.202	0.197	0	0.099	0.017	0.02	0.212	0.262	0.721	0.023	0.085	0.044	0	0	0	0.000	0.718	0.760
TCGA-97-7937-01A-11R-2170-07	0.074	0	0.193	0.024	0	0.064	0	0.088	0.053	0	0.046	0	0	0.392	0.062	0.353	0	0.007	0.165	0.004	0	0.009	0.000	0.608	0.812
TCGA-97-7938-01A-11R-2170-07	0.206	0	0.015	0.001	0	0.308	0.018	0.148	0	0	0.042	0	0.017	0.244	0.084	0.449	0.009	0.086	0.133	0.013	0	0.024	0.000	0.421	0.906
TCGA-J2-A4AE-01A-21R-A24H-07	0.272	0.029	0.225	0	0	0.388	0	0.315	0	0	0.017	0	0.03	0.174	0.136	0.512	0.02	0.109	0.098	0	0	0.002	0.000	0.482	0.880
TCGA-L4-A4E5-01A-11R-A24X-07	0	0.028	0.194	0.077	0	0.219	0.009	0.181	0.14	0	0.041	0.023	0	0.297	0.085	0.292	0.014	0.008	0.046	0	0	0	0.000	0.684	0.778
TCGA-L9-A443-01A-12R-A24H-07	0.106	0	0.051	0.144	0	0.388	0	0.173	0	0	0.026	0	0	0.339	0.193	0.411	0.036	0.002	0.108	0	0	0	0.000	0.466	0.884
TCGA-05-4250-01A-01R-1107-07	0.145	0	0.112	0.372	0	0.467	0.125	0.293	0.045	0	0.076	0.078	0.014	0.402	0.26	0.848	0	0.036	0.095	0	0	0.03	0.000	0.626	0.803
TCGA-05-4398-01A-01R-1206-07	0.172	0.031	0.338	0.263	0	0.816	0.181	0.32	0.04	0	0.166	0	0.231	0.533	0.334	1.388	0.137	0.08	0.107	0	0	0.022	0.000	0.763	0.736
TCGA-05-4433-01A-22R-1858-07	0.066	0	0	0.104	0	0.324	0	0.062	0.032	0	0.072	0	0.024	0.522	0.119	0.355	0.025	0	0.085	0	0	0.019	0.000	0.638	0.784
TCGA-38-4626-01A-01R-1206-07	0.009	0	0.093	0.05	0	0.878	0.109	0.009	0	0	0.295	0	0.524	1.188	0.063	2.51	0	0.268	0.516	0	0	0.173	0.000	0.554	0.835
TCGA-44-3919-01A-02R-1107-07	0.166	0.058	0.133	0.205	0	0.566	0	0.345	0.204	0	0.083	0.004	0.026	0.437	0.404	0.819	0.165	0.052	0.339	0	0	0.018	0.000	0.840	0.689
TCGA-44-6148-01A-11R-1755-07	0.093	0.113	0.308	0.051	0	0.164	0	0.154	0.023	0	0.007	0.012	0.102	0.167	0.026	0.469	0.006	0.042	0.441	0	0	0.012	0.000	0.595	0.835
TCGA-44-6775-01C-02R-A277-07	0.132	0	0.14	0	0.017	0.227	0.025	0.098	0	0	0.004	0	0	0.109	0.034	0.231	0.006	0.042	0.089	0	0.063	0.013	0.000	0.525	0.866
TCGA-44-A4SU-01A-11R-A24X-07	0.158	0.135	0.346	0.072	0	0.175	0	0.323	0.125	0	0.053	0	0.018	0.383	0.139	0.158	0.018	0.063	0.068	0.008	0	0	0.000	0.718	0.772
TCGA-49-4501-01A-01R-1206-07	0.27	0.196	0.202	0.23	0	0.957	0	0.11	0.141	0	0.005	0.019	0.121	0.275	0.177	0.828	0.073	0.217	0.343	0	0	0.005	0.000	0.749	0.753
TCGA-49-6761-01A-31R-1949-07	0.074	0.033	0.039	0.153	0	0.188	0.03	0.173	0.097	0	0.115	0.052	0.017	0.264	0.1	0.333	0	0.062	0.013	0	0	0.005	0.000	0.729	0.741
TCGA-50-5044-01A-21R-1858-07	0	0.022	0.07	0.129	0	0.18	0	0.053	0.101	0	0	0.073	0.008	0.133	0.073	0.337	0.002	0.067	0.069	0	0	0.003	0.000	0.701	0.763
TCGA-50-5051-01A-21R-1858-07	0.111	0.053	0.129	0.076	0	0.027	0	0.149	0.052	0	0.026	0.014	0.015	0.129	0	0.19	0.032	0.112	0.081	0.001	0	0.002	0.000	0.516	0.869
TCGA-50-5946-01A-11R-1755-07	0.121	0	0.207	0.082	0	0.153	0	0.194	0.041	0	0.063	0.009	0.037	0.178	0.124	0.377	0	0.046	0.105	0	0	0	0.000	0.413	0.911
TCGA-55-6969-01A-11R-1949-07	0.027	0.079	0.656	0.211	0	0.701	0.027	0.304	0.16	0	0.166	0	0	0.955	0.273	0.48	0	0	0.003	0.023	0	0	0.000	0.808	0.704
TCGA-55-6970-01A-11R-1949-07	0.128	0.055	0.252	0.155	0	0.607	0.065	0.178	0.011	0	0.052	0.017	0.082	0.376	0.129	0.759	0	0.401	0.142	0	0	0.029	0.000	0.695	0.775
TCGA-55-7576-01A-11R-2066-07	0.166	0	0.075	0.19	0	0.48	0.059	0.213	0.166	0	0.025	0	0.12	0.401	0.151	0.873	0.096	0.117	0.271	0	0	0.024	0.000	0.714	0.761
TCGA-55-7815-01A-11R-2170-07	0.077	0	0.039	0.019	0	0.46	0.055	0.017	0.008	0.014	0.01	0	0.007	0.101	0.067	0.401	0.041	0.042	0.056	0	0	0.014	0.000	0.702	0.765
TCGA-55-8620-01A-11R-2403-07	0.092	0.013	0.171	0.092	0	0.181	0.02	0.13	0.121	0	0.083	0	0	0.348	0.143	0.266	0	0	0.039	0	0	0	0.000	0.768	0.727
TCGA-55-A491-01A-11R-A24H-07	0.192	0	0.155	0.12	0	0.209	0	0.135	0.204	0	0.084	0.001	0.057	0.549	0.2	0.341	0	0	0.013	0.016	0	0	0.000	0.749	0.729
TCGA-62-A46P-01A-11R-A24H-07	0.057	0	0.104	0.055	0	0.164	0	0.054	0.038	0	0.03	0.018	0.021	0.197	0.012	0.302	0	0.037	0.034	0.007	0	0.008	0.000	0.446	0.895
TCGA-64-5778-01A-01R-1628-07	0.15	0.106	0.198	0.632	0	0.312	0.091	0.247	0.151	0.028	0	0.006	0	0.37	0.364	0.497	0	0.008	0.043	0	0	0	0.000	0.773	0.713
TCGA-67-3771-01A-01R-0946-07	0.1	0	0.199	0.123	0	0.251	0.031	0.078	0.065	0	0.079	0	0.045	0.461	0.185	0.381	0	0	0.049	0	0	0.002	0.000	0.817	0.691
TCGA-67-6217-01A-11R-1755-07	0.026	0.172	0.149	0.219	0	0.331	0.023	0.117	0.054	0	0.03	0	0.052	0.091	0.116	0.453	0.141	0.078	0.129	0	0	0.003	0.000	0.718	0.773
TCGA-73-4659-01A-01R-1206-07	0.095	0.035	0.178	0.043	0	0.651	0.021	0.028	0.011	0	0.183	0	0.084	0.583	0.05	1.839	0.02	0.103	0.362	0	0	0.108	0.000	0.616	0.801
TCGA-75-5125-01A-01R-1755-07	0.036	0.007	0.062	0.142	0	0.363	0.05	0.217	0.05	0	0.097	0.008	0.042	0.206	0.252	0.738	0.027	0.085	0.116	0	0	0.032	0.000	0.772	0.718
TCGA-75-6203-01A-11R-1755-07	0.07	0	0.045	0.077	0	0.48	0.001	0.033	0.037	0	0.201	0	0.042	0.613	0.063	0.847	0.055	0.027	0.134	0	0	0.024	0.000	0.599	0.809
TCGA-75-6212-01A-11R-1755-07	0.302	0.025	0.125	0.05	0	0.267	0	0.088	0.066	0	0.043	0.04	0.036	0.172	0.019	0.517	0.142	0.1	0.352	0	0	0.007	0.000	0.432	0.903
TCGA-78-7147-01A-11R-2039-07	0.12	0	0.044	0.204	0	0.352	0	0.176	0.073	0	0.041	0.013	0.035	0.268	0.287	0.329	0.039	0.042	0.185	0	0	0	0.000	0.583	0.824
TCGA-86-7701-01A-11R-2170-07	0.081	0.072	0.156	0.1	0	0.318	0.021	0.123	0.122	0	0.053	0.005	0.011	0.102	0.061	0.238	0.009	0.039	0.054	0	0	0.009	0.000	0.754	0.748
TCGA-86-8359-01A-11R-2326-07	0.061	0	0.211	0.395	0	0.25	0.033	0.17	0.229	0	0.091	0.013	0.001	0.297	0.058	0.121	0	0	0	0.041	0	0.06	0.000	0.700	0.756
TCGA-86-8669-01A-11R-2403-07	0.011	0.092	0.147	0.074	0	0.297	0.002	0.121	0.076	0	0.052	0	0.129	0.255	0.051	0.316	0.061	0.075	0.007	0.006	0	0	0.000	0.645	0.802
TCGA-91-6830-01A-11R-1949-07	0.094	0	0.042	0.112	0	0.263	0	0.064	0.006	0	0.067	0.027	0.014	0.317	0.118	0.379	0	0.17	0.156	0	0.003	0.039	0.000	0.555	0.839
TCGA-91-7771-01A-11R-2170-07	0.123	0	0.067	0.216	0	0.656	0.046	0.091	0.015	0	0.028	0.008	0.032	0.277	0.138	0.649	0	0.098	0.073	0	0	0.007	0.000	0.755	0.729
TCGA-97-7941-01A-11R-2187-07	0.164	0.054	0.046	0.141	0	0.425	0	0.204	0.145	0	0.158	0	0.057	0.26	0.141	0.774	0.062	0.05	0.304	0	0	0	0.000	0.675	0.785
TCGA-97-8547-01A-11R-2403-07	0	0.007	0.001	0.011	0	0.155	0	0.048	0.109	0	0.014	0.008	0.077	0.668	0.021	0.374	0.04	0.147	0.104	0	0	0	0.000	0.789	0.660
TCGA-97-8552-01A-11R-2403-07	0.08	0.145	0.028	0.088	0	0.291	0	0.16	0.042	0	0.027	0.08	0.12	0.182	0.055	0.63	0.065	0.11	0.253	0	0	0.015	0.000	0.686	0.782
TCGA-99-8025-01A-11R-2241-07	0.183	0	0.185	0.035	0	0.15	0	0.123	0.156	0	0.031	0.014	0	0.682	0.105	0.521	0	0.055	0.061	0	0	0.011	0.000	0.725	0.733
TCGA-L9-A50W-01A-12R-A39D-07	0.064	0	0.185	0.069	0	0.399	0	0.244	0.099	0	0	0.145	0.055	0.122	0.081	0.407	0.17	0.054	0.365	0	0	0.018	0.000	0.540	0.857
TCGA-L9-A5IP-01A-21R-A39D-07	0.001	0.015	0.327	0.025	0	0.3	0.017	0.29	0.173	0	0.134	0.081	0.018	0.343	0.094	0.335	0.006	0.012	0.094	0	0.002	0.083	0.002	0.310	0.954
TCGA-L9-A743-01A-43R-A39D-07	0.124	0.238	0.125	0.167	0	0.496	0.159	0.337	0.128	0	0.163	0	0.052	0.386	0.185	0.711	0.04	0.276	0.129	0	0	0	0.000	0.774	0.735
TCGA-L9-A7SV-01A-11R-A39D-07	0.124	0	0.065	0.145	0	0.322	0	0.226	0.061	0	0.01	0.021	0.034	0.1	0.144	0.413	0.009	0.014	0.038	0	0	0	0.000	0.328	0.950
TCGA-L9-A8F4-01A-11R-A39D-07	0.112	0.098	0.368	0.33	0	0.212	0.147	0.307	0.256	0.018	0.039	0	0	0.668	0.217	0.568	0	0	0.016	0	0	0	0.000	0.833	0.692
TCGA-05-4415-01A-22R-1858-07	0.044	0	0.266	0.126	0	0.253	0.079	0.074	0.039	0	0.14	0.04	0.015	0.151	0.114	0.217	0	0.073	0.069	0	0	0.035	0.000	0.453	0.893
TCGA-05-4434-01A-01R-1206-07	0.321	0.074	0.132	0.234	0	0.912	0.196	0.247	0.059	0	0.326	0	0.064	1.123	0.731	1.594	0	0.021	0.357	0	0	0.017	0.000	0.805	0.690
TCGA-35-4123-01A-01R-1107-07	0.079	0	0.061	0.797	0	0.57	0.123	0.402	0.071	0	0.016	0.069	0.068	0.635	0.105	0.681	0.038	0.041	0.047	0.081	0.02	0	0.000	0.757	0.718
TCGA-35-5375-01A-01R-1628-07	0.03	0.042	0.073	0.11	0	0.102	0.003	0.147	0.026	0.063	0	0	0	0.097	0.104	0.274	0.005	0.033	0.06	0	0	0.015	0.000	0.701	0.768
TCGA-44-2668-01A-01R-0946-07	0.108	0	0.002	0.391	0	0.794	0.294	0.076	0.068	0	0	0.283	0.253	1.714	0.604	0.789	0	0.007	0	0.344	0.011	0.09	0.000	0.801	0.666
TCGA-44-3917-01A-01R-A278-07	0.017	0	0.247	0	0	0.29	0.049	0.149	0	0.079	0	0.053	0.009	0	0.146	0.453	0.035	0	0.071	0	0	0.027	0.000	0.612	0.821
TCGA-44-5644-01A-21R-2039-07	0.079	0	0.238	0.083	0	0.111	0	0.097	0.011	0	0.009	0.031	0	0.184	0.02	0.117	0	0.072	0.027	0	0	0.017	0.002	0.304	0.955
TCGA-44-6145-01A-11R-1755-07	0.127	0.049	0.27	0.286	0	0.294	0.087	0.143	0.068	0	0.045	0.071	0.002	0.221	0.152	0.473	0	0.098	0.111	0	0	0.06	0.000	0.748	0.754
TCGA-44-7669-01A-21R-2066-07	0.133	0.022	0.067	0.034	0	0.626	0.049	0.144	0.066	0	0.179	0	0.04	0.367	0.173	0.931	0	0.15	0.197	0	0	0.028	0.000	0.702	0.764
TCGA-44-7671-01A-11R-2066-07	0.014	0.033	0.231	0	0	0.252	0	0.104	0	0	0.104	0	0	0.375	0.022	0.617	0	0.183	0.178	0	0	0.004	0.000	0.524	0.857
TCGA-49-4506-01A-01R-1206-07	0.093	0.051	0.055	0.685	0	0.158	0.192	0.311	0.072	0	0.107	0.036	0.009	0.328	0.235	0.561	0.056	0	0	0	0.007	0.032	0.000	0.633	0.794
TCGA-49-4507-01A-01R-1206-07	0.055	0	0.287	0.804	0	0.09	0.248	0.442	0.22	0	0.321	0.117	0.017	0.389	0.258	0.502	0	0	0.076	0	0	0	0.000	0.645	0.786
TCGA-50-5930-01A-11R-1755-07	0.046	0.027	0.148	0.1	0	0.131	0.012	0.092	0.024	0	0.043	0	0.004	0.312	0.053	0.222	0	0	0.019	0	0	0	0.000	0.696	0.764
TCGA-50-5939-01A-11R-1628-07	0.014	0.164	0.248	0	0	0.28	0	0.121	0.087	0	0	0.071	0	0.482	0.093	0.448	0	0	0.043	0	0	0.042	0.000	0.671	0.780
TCGA-50-6593-01A-11R-1755-07	0.014	0.07	0.009	0.045	0	0.129	0	0.085	0.07	0	0.029	0	0.078	0.62	0.027	0.484	0.049	0.07	0.147	0	0	0.024	0.000	0.821	0.649
TCGA-50-7109-01A-11R-2039-07	0.098	0	0.191	0.081	0	0.277	0.072	0.168	0.067	0	0.097	0	0.028	0.158	0.135	0.292	0	0.035	0.028	0	0	0.005	0.000	0.671	0.790
TCGA-53-7624-01A-11R-2066-07	0.173	0	0.059	0.065	0	0.079	0.014	0.189	0.117	0	0.049	0.028	0	0.614	0.371	0.363	0	0.078	0.015	0	0	0.007	0.000	0.549	0.836
TCGA-55-6979-01A-11R-1949-07	0.052	0.034	0.144	0.102	0	0.429	0.033	0.164	0.179	0	0.111	0	0.023	0.461	0.33	0.741	0.007	0	0.086	0	0	0.007	0.000	0.850	0.661
TCGA-55-7570-01A-11R-2039-07	0.126	0	0.261	0.153	0	0.08	0	0.149	0.037	0	0.008	0.031	0.006	0.21	0.063	0.171	0	0	0.093	0	0	0.002	0.000	0.505	0.874

Input Sample	B cells naive	B cells memory	Plasma cells	T cells CD8	T cells CD4 naive	T cells CD4 memory resting	T cells CD4 memory activated	T cells follicular helper	T cells regulatory (Tregs)	T cells gamma delta	NK cells resting	NK cells activated	Monocytes	Macrophages M0	Macrophages M1	Macrophages M2	Dendritic cells resting	Dendritic cells activated	Mast cells resting	Mast cells activated	Eosinophils	Neutrophils	P-value	Correlation	RMSE
TCGA-55-7728-01A-11R-2187-07	0.051	0.376	0	0.275	0	0.571	0	0.196	0.198	0	0.103	0.077	0.06	2.379	0.142	0.956	0.058	0	0.171	0	0	0	0.000	0.872	0.584
TCGA-55-A48Z-01A-12R-A24X-07	0.001	0.029	0.216	0.189	0	0.207	0	0.127	0.237	0	0.067	0.025	0	0.892	0.166	0.351	0.01	0	0.025	0.023	0	0	0.000	0.798	0.687
TCGA-64-1678-01A-01R-0946-07	0.066	0	0.209	0.042	0	0.085	0.065	0.063	0	0	0.032	0	0	0.037	0.01	0.14	0	0.047	0.004	0	0	0.001	0.002	0.318	0.949
TCGA-67-6215-01A-11R-1755-07	0	0.117	0.023	0.044	0	0.338	0.014	0.017	0.059	0	0.044	0.013	0.039	0.443	0.064	0.737	0.139	0.041	0.343	0	0	0.027	0.000	0.386	0.924
TCGA-69-8254-01A-11R-2287-07	0.255	0.092	0.503	0.221	0	0.638	0.019	0.382	0.007	0.08	0.088	0	0.042	0.279	0.21	0.961	0.055	0.239	0.142	0.018	0	0.023	0.000	0.509	0.870
TCGA-75-5146-01A-01R-1628-07	0.12	0.092	0.218	0.058	0	0.396	0	0.121	0	0	0.03	0	0.019	0.039	0.065	0.383	0.024	0.029	0.058	0	0	0.006	0.000	0.743	0.762
TCGA-75-5147-01A-01R-1628-07	0.254	0	0.28	0	0	0.758	0.041	0.098	0.171	0	0.163	0	0.169	0.386	0.217	0.811	0.07	0.084	0.16	0.054	0	0.057	0.000	0.789	0.732
TCGA-75-6211-01A-11R-1755-07	0.101	0	0.207	0.077	0	0.179	0.022	0.092	0.058	0	0.035	0	0.005	0.093	0.03	0.256	0.008	0.004	0.02	0	0	0.008	0.000	0.584	0.839
TCGA-78-7143-01A-11R-2039-07	0.113	0.04	0.01	0.022	0	0.25	0	0.175	0.131	0	0.027	0.032	0.095	0.271	0.034	0.521	0.073	0.198	0.146	0	0	0.003	0.000	0.744	0.744
TCGA-78-7150-01A-21R-2039-07	0.086	0	0.185	0.065	0	0.132	0.009	0.096	0.045	0	0.039	0.012	0	0.236	0.06	0.16	0	0.058	0	0.005	0	0.006	0.000	0.531	0.857
TCGA-78-7220-01A-11R-2039-07	0.089	0	0.114	0.08	0	0.182	0.025	0.174	0.012	0	0.051	0.007	0.011	0.148	0.048	0.279	0.006	0.011	0.061	0	0	0.013	0.000	0.440	0.899
TCGA-78-7536-01A-11R-2066-07	0.016	0	0.213	0.211	0	0.589	0.088	0.239	0.035	0	0.101	0	0.065	0.181	0.267	0.799	0.012	0	0.01	0	0	0.009	0.000	0.583	0.826
TCGA-86-7711-01A-11R-2066-07	0	0.077	0.149	0.112	0	0.715	0	0.083	0.079	0	0.115	0.034	0.101	1.439	0.188	0.785	0	0.068	0	0.408	0	0.183	0.000	0.393	0.927
TCGA-86-8672-01A-21R-2403-07	0	0.044	0.142	0.249	0	0.18	0.055	0.237	0.117	0	0.136	0	0.024	0.235	0.111	0.376	0	0	0	0.004	0	0.004	0.000	0.683	0.772
TCGA-86-A4D0-01A-11R-A24H-07	0.088	0	0.087	0.021	0	0.24	0.021	0.184	0.079	0	0.066	0.078	0.022	0.064	0.07	0.37	0.02	0.04	0.05	0	0	0.01	0.000	0.546	0.848
TCGA-93-8067-01A-11R-2287-07	0.057	0	0.127	0.033	0	0.296	0	0.314	0.126	0	0.041	0.093	0.001	0.388	0.179	0.77	0.043	0.027	0.002	0.01	0	0.014	0.000	0.517	0.858
TCGA-95-7944-01A-11R-2187-07	0.062	0.003	0.036	0.601	0	0.217	0.242	0.521	0.16	0	0.53	0.119	0.011	0.538	0.355	0.682	0	0.102	0.043	0	0	0.001	0.000	0.713	0.738
TCGA-95-7947-01A-11R-2187-07	0.169	0.011	0.114	0.183	0	0.52	0.009	0.227	0.023	0	0.122	0	0.005	0.33	0.2	0.616	0	0.038	0.092	0	0	0.019	0.000	0.767	0.726
TCGA-95-7948-01A-11R-2187-07	0	0.072	0.247	0.186	0	0.207	0	0.119	0.013	0	0	0.055	0.056	0.083	0.02	0.611	0.018	0.088	0.183	0	0	0.005	0.000	0.340	0.941
TCGA-97-8172-01A-11R-2287-07	0.285	0.725	0.149	0.295	0	0.614	0	0.331	0.104	0	0.017	0.031	0.097	0.423	0.12	0.942	0	0.089	0.696	0	0	0	0.000	0.820	0.716
TCGA-97-8174-01A-11R-2287-07	0.177	0	0.295	0.099	0	0.896	0.011	0.177	0.008	0	0.163	0	0.173	0.032	0.288	0.969	0.003	0.075	0.313	0	0	0	0.000	0.686	0.784
TCGA-97-8179-01A-11R-2287-07	0.134	0	0.108	0.082	0	0.122	0	0.134	0.084	0	0.03	0.042	0.056	0.352	0.084	0.461	0.12	0.037	0.177	0	0.018	0.005	0.000	0.434	0.901
TCGA-05-4395-01A-01R-1206-07	0.01	0	0.264	0.229	0	0.659	0.011	0.241	0.063	0	0.238	0.13	0.105	0.121	0.132	1.012	0.039	0.2	0.168	0	0	0.027	0.000	0.382	0.924
TCGA-05-4397-01A-01R-1206-07	0.211	0	0.211	0.124	0	0.218	0.139	0.179	0.117	0	0.269	0	0.052	0.869	0.451	0.878	0	0.342	0.133	0	0	0.016	0.000	0.494	0.869
TCGA-05-4410-01A-21R-1858-07	0.023	0.096	0.2	0.132	0	0.29	0.042	0.113	0.083	0	0.074	0.016	0.027	0.167	0.165	0.281	0	0.002	0.082	0	0	0.004	0.000	0.724	0.764
TCGA-05-4418-01A-01R-1206-07	0.142	0.031	0.445	0.289	0	0.617	0.053	0.2	0.191	0	0.145	0	0.046	0.452	0.168	0.761	0	0.115	0.034	0	0	0.031	0.000	0.664	0.793
TCGA-05-4425-01A-01R-1755-07	0.021	0.053	0.031	0	0	0.5	0.047	0.114	0.051	0.052	0.067	0	0.027	0.551	0.188	0.78	0.113	0	0.179	0	0	0.013	0.000	0.784	0.701
TCGA-05-5428-01A-01R-1628-07	0.038	0	0.014	0.05	0	0.124	0	0.068	0.01	0.004	0.01	0	0	0.239	0.042	0.481	0.087	0	0.147	0.046	0	0.034	0.000	0.660	0.772
TCGA-05-5715-01A-01R-1628-07	0.046	0.01	0.017	0.016	0	0.142	0	0.024	0.04	0	0.031	0	0	0.7	0.028	0.435	0	0.013	0.092	0	0	0.005	0.000	0.823	0.610
TCGA-44-6776-01A-11R-1858-07	0.116	0	0.127	0.049	0	0.182	0	0.035	0	0	0.003	0.038	0.028	0.039	0.031	0.151	0.044	0.068	0.105	0	0	0	0.000	0.451	0.897
TCGA-44-8117-01A-11R-2241-07	0.098	0	0.121	0	0	0.488	0.004	0.22	0.027	0	0.129	0	0	0.664	0.17	0.731	0	0.03	0.101	0.032	0	0.021	0.000	0.696	0.754
TCGA-49-4505-01A-01R-1206-07	0.065	0.064	0.268	0.322	0	0.627	0	0.307	0.227	0	0.064	0.095	0.162	0.188	0.277	1.02	0.132	0.274	0.431	0	0	0.053	0.000	0.712	0.772
TCGA-49-4514-01A-21R-1858-07	0.116	0.044	0.106	0.176	0	0.146	0.001	0.166	0.096	0	0.021	0.053	0.007	0.149	0.068	0.239	0	0.098	0.035	0	0	0	0.000	0.628	0.807
TCGA-49-6744-01A-11R-1858-07	0.113	0	0.212	0.183	0	0.588	0.003	0.208	0.105	0	0.047	0	0.137	0.361	0.129	0.545	0.142	0.2	0.175	0	0	0.018	0.000	0.836	0.705
TCGA-50-5055-01A-01R-1628-07	0.488	0.217	0.075	0.313	0	0.5	0.002	0.217	0.172	0	0	0.021	0.073	0.54	0.123	0.37	0.031	0.071	0.109	0	0	0	0.000	0.800	0.708
TCGA-50-5068-01A-01R-1628-07	0.079	0.088	0.14	0.18	0	0.319	0.02	0.186	0	0.107	0	0.017	0.024	0.251	0.217	0.41	0.215	0	0.163	0	0	0	0.000	0.762	0.742
TCGA-55-1596-01A-01R-0946-07	0.105	0	0.25	0.024	0	0.13	0.007	0.104	0.107	0	0.05	0.006	0.003	0.33	0.092	0.196	0	0.083	0.079	0	0	0	0.000	0.654	0.800
TCGA-55-6712-01A-11R-1858-07	0.15	0	0.095	0.237	0	0.262	0.057	0.064	0.043	0.02	0.087	0	0.004	0.322	0.171	0.414	0	0.031	0.11	0	0	0	0.000	0.743	0.740
TCGA-55-6987-01A-11R-1949-07	0.122	0.33	0.302	0.618	0	0.505	0.166	0.504	0.228	0	0.329	0.03	0.118	0.737	0.369	0.672	0	0.019	0.214	0	0	0.021	0.000	0.787	0.722
TCGA-55-7227-01A-11R-2039-07	0.047	0.012	0.026	0.128	0	0.313	0	0.075	0.149	0	0.025	0.03	0.048	0.252	0.12	0.456	0.114	0.108	0.147	0	0	0.013	0.000	0.813	0.702
TCGA-55-7907-01A-11R-2170-07	0.111	0	0.082	0.102	0	0.253	0.008	0.156	0.092	0.022	0	0.02	0	0.236	0.182	0.357	0	0	0.068	0	0	0.005	0.000	0.736	0.740
TCGA-55-8094-01A-11R-2241-07	0.131	0	0.148	0.053	0	0.06	0.015	0.2	0	0	0.018	0.005	0	0.17	0	0.237	0	0.26	0.047	0	0	0	0.034	0.172	1.012
TCGA-55-8621-01A-11R-2403-07	0.014	0.051	0.233	0.182	0	0.537	0	0.082	0.051	0	0.219	0	0.103	0.647	0.207	0.678	0.019	0	0.298	0	0	0.011	0.000	0.673	0.780
TCGA-55-A48X-01A-11R-A24H-07	0.253	0.482	0.062	0.147	0	0.478	0	0.139	0.177	0	0.116	0	0.024	0.329	0.088	0.396	0	0.027	0.178	0	0	0	0.000	0.800	0.715
TCGA-67-6216-01A-11R-1755-07	0.014	0.24	0.082	0.138	0	0.387	0	0.065	0.103	0	0.042	0.007	0.189	0.128	0.058	0.438	0.136	0.091	0.241	0	0	0	0.000	0.330	0.945
TCGA-69-8255-01A-11R-2287-07	0.194	0	0.325	0.828	0	0.386	0.317	0.497	0.146	0	0.332	0.007	0.057	0.326	0.33	0.687	0	0	0.054	0	0	0.003	0.000	0.611	0.810
TCGA-73-4670-01A-01R-1206-07	0.131	0.007	0.186	0.089	0	0.204	0	0.165	0.052	0	0.05	0.02	0	0.548	0.058	0.628	0	0.288	0.006	0.018	0	0.133	0.000	0.481	0.876
TCGA-73-7498-01A-12R-2187-07	0.078	0.041	0.336	0.153	0	0.672	0	0.23	0.083	0	0.107	0	0.171	0.146	0.139	0.575	0.018	0.096	0.344	0	0	0	0.000	0.621	0.822
TCGA-73-7499-01A-11R-2187-07	0.071	0.029	0.209	0.726	0	0.406	0.193	0.241	0.181	0	0.038	0	0.032	0.393	0.446	0.681	0.058	0.024	0.081	0	0	0	0.000	0.773	0.715
TCGA-78-7154-01A-11R-2039-07	0.142	0	0.059	0.011	0	0.391	0	0.06	0.002	0	0.067	0.004	0.025	0.07	0.132	0.302	0	0.029	0.085	0	0	0.001	0.000	0.541	0.850
TCGA-78-7167-01A-11R-2066-07	0.075	0	0.223	0.156	0	0.232	0	0.2	0.08	0	0.034	0.079	0.045	0.216	0.086	0.333	0.002	0.028	0.153	0	0	0	0.000	0.351	0.937
TCGA-78-7540-01A-11R-2066-07	0.017	0.138	0.003	0.109	0	0.242	0	0.083	0.096	0	0.065	0.076	0.003	0.938	0.051	0.501	0.008	0.013	0.115	0	0	0	0.000	0.431	0.908
TCGA-80-5611-01A-01R-1628-07	0.04	0.006	0	0.018	0	0.189	0	0.138	0.178	0	0.097	0	0.224	0.822	0.215	0.524	0	0	0.048	0	0.001	0	0.000	0.783	0.673
TCGA-86-6562-01A-11R-1755-07	0.021	0.044	0.072	0	0	0.343	0.019	0.057	0.07	0	0.118	0	0	0.346	0.047	0.513	0.004	0.037	0.037	0.048	0	0.02	0.000	0.711	0.749
TCGA-86-7953-01A-11R-2187-07	0.184	0	0.053	0.145	0	0.894	0.073	0.247	0.122	0	0.162	0	0.245	0.417	0.084	0.84	0	0.128	0	0.087	0	0.045	0.000	0.668	0.782
TCGA-86-8074-01A-11R-2241-07	0.075	0	0.143	0.094	0	0.394	0	0.179	0.134	0	0	0.103	0.076	0.526	0.198	0.632	0.086	0.096	0.06	0	0	0.019	0.000	0.650	0.787
TCGA-86-8075-01A-11R-2241-07	0.246	0	0.373	0.03	0	0.349	0.034	0.188	0.051	0	0.071	0	0.086	0.573	0.062	0.86	0.041	0.347	0.124	0.016	0	0.05	0.000	0.668	0.787
TCGA-86-8076-01A-31R-2241-07	0.098	0.165	0.332	0.434	0	0.405	0.03	0.344	0.255	0	0.121	0	0	0.752	0.201	0.557	0	0.026	0.14	0.003	0	0.007	0.000	0.806	0.708
TCGA-86-8279-01A-11R-2287-07	0	0.077	0.066	0.055	0	0.211	0	0.126	0.088	0	0.049	0.02	0.058	0.859	0.057	0.456	0.099	0.104	0.054	0.002	0	0	0.000	0.691	0.742
TCGA-86-8358-01A-11R-2326-07	0.029	0	0.151	0.022	0	0.084	0	0.175	0.012	0	0.113	0	0	0.265	0.071	0.321	0	0	0.027	0	0	0	0.000	0.746	0.734
TCGA-86-8671-01A-11R-2403-07	0.221	0.279	0	0.392	0	0.581	0	0.426	0.353	0	0.162	0.005	0	0.788	0.487	0.646	0	0	0.2	0	0	0.006	0.000	0.868	0.650
TCGA-97-7553-01A-21R-2039-07	0.192	0.063	0.021	0.278	0	0.704	0.004	0.121	0	0	0.186	0	0.119	1.052	0.163	0.95	0.046	0.055	0.297	0	0	0.115	0.000	0.647	0.784
TCGA-97-8171-01A-11R-2287-07	0.066	0.021	0.151	0.013	0	0.095	0	0.09	0.017	0	0.007	0.011	0	0.662	0.019	0.439	0	0.028	0.076	0	0	0	0.000	0.538	0.844
TCGA-99-7458-01A-11R-2039-07	0.183	0.028	0.006	0.264	0	0.913	0.019	0.307	0.166	0	0.051	0.011	0	0.474	0.189	0.846	0.139	0.206	0.207	0	0	0	0.000	0.700	0.763
TCGA-L4-A4E6-01A-11R-A24H-07	0.058	0.123	0	0.159	0	0.441	0	0.257	0.067	0	0.222	0	0.023	0.822	0.109	1.678	0.019	0.043	0.188	0	0	0.018	0.000	0.483	0.875
TCGA-05-4249-01A-01R-1107-07	0.059	0.109	0.079	0.078	0	0.309	0	0.181	0.128	0	0.009	0.035	0.186	0.113	0.085	0.61	0.517	0.085	0.513	0	0	0.036	0.000	0.736	0.761
TCGA-05-4396-01A-21R-1858-07	0.073	0	0.12	0.008	0	0.217	0.006	0.079	0.017	0	0.071	0	0.013	0.129	0.041	0.178	0	0.018	0.002	0.021	0	0.01	0.000	0.489	0.877
TCGA-05-4430-01A-02R-1206-07	0.07	0.081	0.123	0.141	0	0.401	0.022	0.175	0.132	0	0	0.079	0.042	0.779	0.176	1.042	0.106	0.117	0.317	0	0	0.046	0.000	0.836	0.671
TCGA-35-4122-01A-01R-1107-07	0.091	0	0.305	0.315	0	0.378	0.14	0.321	0.058	0	0.082	0.005	0.112	0.432	0.234	1.269	0.09	0.074	0.045	0	0.02	0.054	0.000	0.727	0.750
TCGA-38-4627-01A-01R-1206-07	0.158	0	0.14	0.062	0	0.708	0.003	0.013	0	0	0.074	0.005	0.242	0.355	0.072	1.217	0.012	0.103	0.216	0	0	0.017	0.000	0.822	0.687
TCGA-38-4629-01A-02R-1206-07	0.066	0	0.166	0.342	0	0.586	0.119	0.158	0.098	0	0.197	0	0.144	0.572	0.433	1.428	0	0	0.137	0	0.048	0.049	0.000	0.811	0.687
TCGA-38-4632-01A-01R-1755-07	0.108	0	0.042	0.099	0	0.24	0.099	0.181	0.032	0	0.03	0	0.074	0.137	0.074	0.527	0.183	0.114	0.151	0	0	0.029	0.000	0.628	0.813
TCGA-38-6178-01A-11R-1755-07	0.142	0	0.161	0	0	0.257	0.001	0.034	0.019	0	0.045	0	0.013	0.078	0.048	0.246	0	0.097	0.13	0	0	0.002	0.000	0.625	0.823
TCGA-44-2659-01A-01R-0946-07	0.144	0	0.287	0.446	0	0.653	0	0.159	0.249	0	0	0.027	0.034	0.16	0.164	0.649	0.008	0.054	0.194	0	0	0.015	0.000	0.685	0.778
TCGA-44-2666-01B-02R-A277-07	0.044	0	0.156	0	0	0.261	0	0.045	0	0	0	0.054	0.014	0	0	0.084	0	0.096	0.224	0	0	0.005	0.000	0.344	0.940
TCGA-44-3918-01A-01R-1107-07	0.089	0.007	0.362	0.394	0	0.795	0.13	0.368	0.106	0	0.171	0.075	0.007	0.589	0.423	1.005	0	0	0.231	0	0	0.048	0.000	0.766	0.730
TCGA-44-5645-01B-04R-A277-07	0.065	0.002	0.028	0	0.023	0.532	0.026	0.034	0	0	0.036	0	0.043	0.026	0.051	0.183	0.087	0.06	0.127	0	0.051	0	0.000	0.556	0.844
TCGA-44-7660-01A-11R-2066-07	0.174	0	0.123	0.109	0	0.227	0	0.262	0.051	0	0.07	0.026	0	0.218	0.205	0.54	0.014	0	0.057	0	0	0.001	0.000	0.500	0.869
TCGA-44-7667-01A-31R-2066-07	0.036	0	0.093	0.017	0	0.244	0.018	0.083	0.011	0	0.057	0.079	0.034	0.665	0.161	0.387	0	0	0	0.015	0	0	0.000	0.774	0.678
TCGA-49-4486-01A-01R-1206-07	0.103	0	0.356	0	0	0.346	0	0.227	0.032	0.031	0.051	0.002	0.013	0	0.053	0.418	0	0.066	0.014	0	0	0.017	0.000	0.383	0.924
TCGA-49-4512-01A-21R-1858-07	0.033	0.077	0	0.037	0	0.039	0	0.09	0.121	0	0.031	0	0.094	0.388	0.037	0.419	0.021	0.063	0.107	0	0	0	0.000	0.785	0.692
TCGA-49-6745-01A-11R-1858-07	0.004	0.027	0.068	0.185	0	0.391	0.053	0.164	0.08	0	0.071	0	0.157	0.383	0.121	0.544	0.037	0.153	0.164	0	0	0.006	0.000	0.758	0.735
TCGA-50-5049-01A-01R-1628-07	0.068	0.182	0.312	0.399	0	0.637	0.085	0.286	0.136	0	0.062	0	0.047	0.701	0.328	0.83	0.051	0	0.041	0	0	0	0.000	0.814	0.698
TCGA-50-6591-01A-11R-1755-07	0.122	0	0.032	0.015	0	0.112	0	0.017	0.032	0	0	0	0	0.378	0.007	0.06	0	0	0	0.015	0	0	0.000	0.394	0.935
TCGA-50-6592-01A-11R-1755-07	0.181	0	0.137	0.184	0	0.56	0.085	0.141	0.034	0	0.263	0.063	0.034	0.633	0.34	0.518	0	0	0.093	0	0	0.021	0.000	0.764	0.717
TCGA-53-7626-01A-12R-2066-07	0.17	0.229	0.214	0.431	0	1.055	0	0.369	0.075	0	0.191	0	0.233	0.592	0.306	0.921	0.059	0.046	0.126	0.166	0	0	0.000	0.657	0.792
TCGA-55-6968-01A-11R-1949-07	0.05	0.002	0.18	0.078	0	0.219	0	0.141	0.116	0.013	0.048	0.023	0	0.715	0.188	0.434	0	0	0.025	0	0	0	0.000	0.830	0.650
TCGA-55-7914-01A-11R-2170-07	0.058	0	0.217	0.103	0	0.398	0	0.16	0.102	0	0.027	0.029	0.017	0.169	0.147	0.29	0	0.02	0.183	0	0	0	0.000	0.628	0.813
TCGA-55-8087-01A-11R-2241-07	0	0.085	0	0.041	0	0.255	0	0.063	0.05	0	0	0.052	0.046	0.288	0.028	0.534	0.302	0.027	0.322	0	0	0.02	0.000	0.570	0.832
TCGA-55-A492-01A-11R-A24H-07	0.011	0.012	0.296	0.073	0	0.274	0	0.068	0.041	0	0.051	0.016	0.036	0.13	0.046	0.359	0	0.058	0.093	0	0	0.001	0.000	0.464	0.892
TCGA-55-A494-01A-11R-A24X-07	0.044	0	0.149	0.089	0	0	0	0.344	0.049	0	0	0.04	0.003	0.148	0	0.263	0.012	0.108	0.022	0	0	0.002	0.018	0.187	1.007
TCGA-64-1681-01A-11R-2066-07	0.033	0.144	0.169	0.059	0	0.445	0	0.142	0.05	0	0.04	0	0.072	0.41	0.29	0.599	0.214	0.139	0.25	0	0	0.005	0.000	0.704	0.767
TCGA-69-7760-01A-11R-2170-07	0.087	0	0.054	0	0	0.048	0.001	0.104	0.015	0	0.035	0	0.014	0.186	0	0.237	0	0.321	0.027	0	0	0.005	0.000	0.455	0.890
TCGA-69-7973-01A-11R-2187-07	0.225	0	0.195	0.124	0	0.466	0.108	0.128	0.061	0	0.069	0.005	0.051	0.171	0.09	0.724	0	0.077	0.236	0	0	0.025	0.000	0.510	0.870
TCGA-69-7974-01A-11R-2187-07	0.092	0.114	0.066	0.119	0	0.688	0.009	0.219	0	0	0.177	0.033	0.105	0.475	0.205	1.388	0.119	0.136	0.167	0	0	0	0.000	0.720	0.747
TCGA-69-7979-01A-11R-2187-07	0.127	0	0.25	0.048	0	0.186	0	0.168	0	0	0.114	0	0.001	0.394	0.15	0.511	0	0.093	0.1	0	0	0.007	0.000	0.499	0.870
TCGA-73-4677-01A-01R-1206-07	0.134	0.031	0.335	0.224	0	0.661	0.015	0.065	0.007	0	0.069	0.021	0.058	0.184	0.106	0.76	0.04	0.098	0.129	0	0	0.032	0.000	0.551	0.850
TCGA-75-5122-01A-01R-1755-07	0.042	0	0.084	0.717	0	0.44	0.053	0.236	0.052	0.014	0	0.025	0.149	0.051	0.267	1.214	0.164	0	0.001	0.073	0.013	0.019	0.000	0.771	0.713
TCGA-78-7149-01A-11R-2039-07	0.059	0	0.289	0.099	0	0.095	0	0.126	0.071	0	0.038	0.029	0.024	0.102	0.061	0.185	0	0.029	0.135	0	0	0	0.000	0.459	0.896
TCGA-86-6851-01A-11R-1949-07	0.101	0.146	0.722	0.394	0	0.624	0.037	0.531	0.256	0	0.37	0.075	0.009	0.634	0.476	0.872	0	0	0.184	0	0	0	0.000	0.730	0.757
TCGA-91-6831-01A-11R-1858-07	0.014	0.009	0.147	0.041	0	0.213	0.016	0.036	0.059	0	0.058	0.001	0.01	0.343	0.116	0.26	0	0	0.042	0	0	0.023	0.000	0.804	0.694
TCGA-91-6836-01A-21R-1858-07	0.038	0.002	0.2	0.097	0	0.243	0.083	0.111	0	0	0.064	0.018	0.038	0.299	0.061	0.254	0	0.078	0.012	0.01	0.003	0.026	0.000	0.524	0.860
TCGA-91-6848-01A-11R-1949-07	0.023	0.009	0.072	0.062	0	0.422	0.056	0.081	0	0	0.13	0	0.084	0.362	0.194	0.415	0	0.003	0.016	0	0	0.004	0.000	0.745	0.726
TCGA-97-7546-01A-11R-2039-07	0.155	0.04	0.059	0.123	0	0.627	0	0.054	0.029	0	0.062	0	0.1	0.237	0.14	0.408	0.026	0.092	0.267	0	0	0.02	0.000	0.681	0.783

Input Sample	B cells naive	B cells memory	Plasma cells	T cells CD8	T cells CD4 naive	T cells CD4 memory resting	T cells CD4 memory activated	T cells follicular helper	T cells regulatory (Tregs)	T cells gamma delta	NK cells resting	NK cells activated	Monocytes	Macrophages M0	Macrophages M1	Macrophages M2	Dendritic cells resting	Dendritic cells activated	Mast cells resting	Mast cells activated	Eosinophils	Neutrophils	P-value	Correlation	RMSE
TCGA-99-8032-01A-11R-2241-07	0.037	0.134	0.457	0.121	0	0.299	0.017	0.163	0.135	0	0.07	0	0.02	0.356	0.046	0.356	0	0.032	0.161	0	0	0.005	0.000	0.695	0.793
TCGA-J2-8192-01A-11R-2241-07	0.031	0.124	0.019	0.022	0	0.851	0	0.031	0	0	0.167	0	0.058	0.667	0.082	0.951	0.121	0.131	0.231	0	0	0.048	0.000	0.827	0.676
TCGA-44-8120-01A-11R-2241-07	0.156	0.056	0.068	0.136	0	0.471	0.02	0.141	0.052	0	0.063	0	0.007	0.308	0.044	0.858	0.095	0.137	0.398	0	0	0.052	0.000	0.537	0.853
TCGA-44-A47B-01A-11R-A24H-07	0.036	0.019	0	0.192	0	0.301	0	0.135	0.214	0	0	0.097	0.009	0.388	0.137	0.52	0.017	0.035	0.217	0	0	0.014	0.000	0.667	0.778
TCGA-50-5045-01A-01R-1628-07	0.384	0.024	0.237	0.301	0	0.545	0	0.449	0.221	0	0	0.028	0.103	0.322	0.275	1.13	0.157	0.145	0.234	0	0	0.015	0.000	0.835	0.702
TCGA-55-7724-01A-11R-2170-07	0.049	0.037	0.072	0	0	0.242	0.01	0.054	0	0.067	0.016	0	0	0.271	0.092	0.48	0.039	0.04	0.088	0	0	0.062	0.000	0.758	0.723
TCGA-55-8206-01A-11R-2241-07	0.029	0.158	0.159	0.061	0	0.417	0.026	0.202	0.089	0	0.006	0.048	1.009	0.279	0	0.774	0.345	0.173	0.617	0	0	0	0.000	0.787	0.726
TCGA-55-8207-01A-11R-2241-07	0.152	0	0.033	0.1	0	0.564	0	0.082	0.028	0	0.065	0	0.152	0.598	0.048	1.008	0.085	0.101	0.331	0	0	0.022	0.000	0.658	0.781
TCGA-55-8505-01A-11R-2403-07	0.035	0.014	0.218	0.031	0	0.124	0.001	0.032	0.071	0	0.028	0.005	0	0.49	0.036	0.21	0	0	0.083	0	0	0.018	0.000	0.668	0.770
TCGA-55-8506-01A-11R-2403-07	0.026	0.007	0.11	0.019	0	0.131	0	0.187	0.088	0	0.124	0	0.005	0.625	0.13	0.341	0.015	0.013	0.076	0.062	0	0.012	0.000	0.753	0.710
TCGA-55-8507-01A-11R-2403-07	0.008	0.04	0.033	0.088	0	0.139	0	0.092	0.099	0	0.063	0.047	0.046	0.545	0.035	0.28	0	0.065	0.123	0.03	0	0.038	0.000	0.662	0.771
TCGA-55-8508-01A-11R-2403-07	0.003	0.061	0.295	0.059	0	0.1	0.016	0.079	0.091	0	0.067	0	0	0.426	0.032	0.111	0	0	0	0.033	0	0.018	0.000	0.736	0.746
TCGA-62-8394-01A-11R-2326-07	0.051	0.015	0.039	0.077	0	0.174	0	0.195	0.184	0	0	0.091	0.077	0.634	0.142	0.383	0	0.1	0.092	0	0	0	0.000	0.686	0.757
TCGA-62-8395-01A-11R-2326-07	0.098	0.045	0.101	0.054	0	0.241	0.002	0.158	0.097	0	0.083	0.004	0.06	0.301	0.06	0.44	0.032	0.037	0.118	0	0	0	0.000	0.587	0.829
TCGA-62-8397-01A-11R-2326-07	0.018	0	0.072	0.034	0	0.103	0	0.015	0.024	0	0.005	0.018	0.072	0.11	0	0.153	0.062	0.016	0.12	0	0	0	0.000	0.533	0.858
TCGA-62-8398-01A-11R-2326-07	0.074	0	0.084	0	0	0.545	0.014	0.182	0.138	0	0.153	0.014	0.039	0.535	0.158	0.572	0	0.03	0.048	0	0	0	0.000	0.645	0.788
TCGA-62-8402-01A-11R-2326-07	0.108	0	0	0.115	0	0.524	0.123	0.133	0.169	0	0.105	0.031	0	0.736	0.416	0.328	0	0	0.078	0	0	0.025	0.000	0.783	0.686
TCGA-62-A46R-01A-11R-A24H-07	0.093	0.032	0.009	0.129	0	0.334	0	0.165	0.08	0	0.03	0.032	0.211	0.234	0.024	0.466	0.012	0.268	0.3	0	0	0	0.000	0.620	0.814
TCGA-62-A46U-01A-11R-A24H-07	0	0.025	0	0.181	0	0.863	0.103	0.299	0.154	0	0.022	0.08	0	1.079	0.576	0.74	0.029	0	0.154	0	0	0.042	0.000	0.849	0.614
TCGA-62-A471-01A-12R-A24H-07	0.092	0	0.183	0.095	0	0.074	0.005	0.228	0.092	0	0.183	0.018	0.001	0.231	0.028	0.135	0	0.071	0.063	0	0	0.02	0.000	0.428	0.903
TCGA-64-5781-01A-01R-1628-07	0	0.015	0.173	0.479	0	0.156	0.097	0.277	0.112	0	0	0.203	0.015	0.206	0.088	0.179	0	0.081	0.022	0	0	0	0.000	0.674	0.763
TCGA-67-3772-01A-01R-0946-07	0.107	0.026	0.296	0.106	0	0.422	0	0.112	0.044	0	0	0.014	0.101	0.228	0.055	0.695	0.144	0.123	0.192	0	0	0.011	0.000	0.748	0.759
TCGA-75-5126-01A-01R-1755-07	0.085	0	0.017	0.181	0	0.214	0.104	0.06	0.02	0	0.13	0	0.075	0.44	0.22	0.76	0.001	0.021	0.09	0	0.031	0.097	0.000	0.707	0.746
TCGA-78-7146-01A-11R-2039-07	0.025	0	0.02	0.001	0	0.421	0.049	0.057	0.02	0	0.088	0	0.014	0.33	0.106	0.358	0.009	0.064	0	0.054	0	0.008	0.000	0.570	0.828
TCGA-78-7156-01A-11R-2039-07	0.068	0	0.187	0.003	0	0.2	0	0.069	0.014	0.007	0.024	0.015	0.012	0.041	0.099	0.171	0	0.019	0.151	0	0	0.001	0.000	0.395	0.919
TCGA-78-8648-01A-11R-2403-07	0.046	0.08	0.256	0.205	0	0.3	0	0.181	0.081	0	0.159	0	0.005	1.062	0.075	0.589	0	0	0.125	0	0	0	0.000	0.869	0.627
TCGA-78-8655-01A-11R-2403-07	0.096	0	0	0.136	0	0.241	0	0.068	0.127	0	0.007	0.058	0.024	0.549	0.107	0.411	0	0	0.122	0	0	0.016	0.000	0.807	0.675
TCGA-78-8662-01A-11R-2403-07	0.087	0	0.143	0.055	0	0.13	0	0.084	0.029	0	0.009	0.011	0.009	0.091	0.061	0.163	0	0.026	0	0.027	0	0	0.000	0.396	0.918
TCGA-86-A4P7-01A-11R-A24X-07	0.167	0.092	0.226	0.216	0	0.369	0	0.281	0.095	0	0.115	0.005	0.032	0.474	0.065	0.63	0	0.02	0.204	0	0	0.003	0.000	0.608	0.820
TCGA-91-8496-01A-11R-2403-07	0.023	0.004	0	0.089	0	0.263	0	0.016	0.037	0	0.062	0.014	0.194	0.299	0.038	0.554	0.11	0.006	0.265	0	0.028	0.02	0.000	0.678	0.771
TCGA-91-8499-01A-11R-2403-07	0.065	0	0.083	0.103	0	0.305	0.004	0.201	0	0.004	0.047	0.005	0.012	0.292	0.163	0.535	0.001	0	0.034	0	0.009	0	0.000	0.588	0.819
TCGA-91-A4BC-01A-11R-A24H-07	0.097	0	0.307	0.685	0	0.132	0.201	0.313	0.081	0	0.131	0.049	0.035	0.152	0.152	0.385	0	0	0.108	0	0	0	0.000	0.740	0.740
TCGA-95-A4VK-01A-11R-A262-07	0.297	0.078	0.162	0.183	0	0.17	0	0.275	0.12	0	0.058	0.001	0.01	0.344	0.144	0.254	0	0.079	0.079	0	0	0	0.000	0.601	0.822
TCGA-97-8175-01A-11R-2287-07	0	0.138	0	0.063	0	0.271	0	0.202	0.12	0	0.075	0.013	0.237	0.549	0.069	0.791	0.271	0.175	0.173	0	0	0.052	0.000	0.676	0.772
TCGA-97-A4M2-01A-12R-A24X-07	0.137	0.054	0.182	0.334	0	0.203	0	0.304	0.166	0	0.159	0.024	0.144	0.653	0.091	0.729	0.065	0.105	0.32	0	0	0.011	0.000	0.681	0.781
TCGA-97-A4M7-01A-11R-A24X-07	0.146	0.117	0.072	0.19	0	0.34	0	0.329	0.244	0	0	0.068	0.185	0.156	0.117	0.654	0.135	0.217	0.431	0	0	0.021	0.000	0.748	0.757
TCGA-99-8033-01A-11R-2241-07	0.036	0	0.256	0.398	0	0	0.214	0.458	0.041	0	0.316	0.097	0.044	0.456	0.208	0.345	0	0.234	0.034	0.011	0	0.059	0.000	0.523	0.856
TCGA-J2-A4AG-01A-11R-A24H-07	0.191	0.284	0.002	0.156	0	0.36	0.011	0.184	0.065	0	0.066	0.007	0.034	0.168	0.068	0.649	0.05	0.202	0.056	0.005	0	0.005	0.000	0.633	0.804
TCGA-MP-A4SY-01A-21R-A24X-07	0.002	0.023	0.042	0	0	0.209	0	0.066	0.143	0	0.059	0.001	0	0.867	0.051	0.502	0	0.018	0.113	0	0	0.017	0.000	0.744	0.692
TCGA-MP-A4T4-01A-11R-A262-07	0.032	0.032	0.249	0.34	0	0.451	0.05	0.238	0.216	0	0.243	0	0.033	0.434	0.275	0.589	0	0.029	0.136	0	0	0	0.000	0.787	0.716
TCGA-MP-A4T6-01A-32R-A262-07	0.093	0.067	0.066	0.18	0	0.2	0	0.346	0.203	0	0.08	0.018	0	0.814	0.16	0.409	0	0.041	0.138	0	0	0.006	0.000	0.708	0.746
TCGA-MP-A4T7-01A-11R-A24X-07	0.064	0.048	0.051	0.141	0	0.304	0	0.27	0.141	0	0.074	0.049	0.043	0.355	0.127	0.56	0	0.207	0.083	0	0	0.022	0.000	0.531	0.852
TCGA-MP-A4T8-01A-11R-A24X-07	0.087	0	0.088	0	0.01	0.021	0	0.107	0.133	0.006	0.03	0.037	0	0.677	0	0.289	0	0.255	0.08	0	0	0.007	0.000	0.653	0.769
TCGA-MP-A4TC-01A-11R-A24X-07	0.018	0.036	0.075	0.134	0	0.323	0.064	0.14	0.143	0	0.199	0	0	0.625	0.272	0.576	0	0	0.054	0.026	0	0.002	0.000	0.756	0.713
TCGA-MP-A4TD-01A-32R-A262-07	0.143	0.27	0.145	0.083	0	0.325	0	0.238	0.079	0	0.011	0	0	0.503	0.026	0.41	0	0.062	0.101	0	0	0	0.000	0.762	0.732
TCGA-MP-A4TH-01A-31R-A262-07	0.178	0.54	0.302	0.258	0	0.322	0	0.341	0.15	0	0.025	0.023	0.006	0.255	0.102	0.567	0.036	0.081	0.109	0	0	0	0.000	0.774	0.741
TCGA-MP-A4TI-01A-21R-A24X-07	0.207	0	0	0.337	0	0.307	0.092	0.449	0.261	0	0.098	0.046	0.04	0.695	0.396	1.072	0	0.131	0.239	0	0	0.019	0.000	0.844	0.668
TCGA-MP-A4TJ-01A-51R-A262-07	0.111	0.152	0.114	0.099	0	0.487	0.126	0.264	0.053	0	0.2	0	0.001	0.38	0.205	0.415	0	0.035	0.066	0	0	0.018	0.000	0.833	0.692
TCGA-MP-A4TK-01A-11R-A24X-07	0.062	0.155	0.243	0.031	0	0.396	0	0.142	0.122	0	0.174	0	0	0.816	0.119	0.652	0	0.024	0.066	0	0	0.013	0.000	0.849	0.659
TCGA-05-5429-01A-01R-1628-07	0.044	0	0.092	0	0	0.361	0	0.081	0.036	0	0.014	0	0.014	0.085	0.032	0.288	0	0.017	0.015	0.049	0	0.009	0.000	0.420	0.907
TCGA-38-4625-01A-01R-1206-07	0.049	0.121	0.215	0.123	0	0.528	0.011	0.239	0	0.037	0.056	0.016	0	0.898	0.369	1.096	0	0.051	0.217	0	0.006	0.048	0.000	0.661	0.775
TCGA-44-6147-01B-06R-A277-07	0.071	0.183	0.06	0	0.033	0.409	0.02	0.064	0	0.03	0	0.021	0	0.002	0.047	0.193	0	0.041	0.053	0	0.006	0.013	0.000	0.510	0.864
TCGA-44-A47A-01A-21R-A24H-07	0	0.08	0	0.083	0	0.276	0.024	0.178	0.145	0	0.031	0.001	0.052	0.548	0.04	0.512	0.233	0.23	0.235	0	0	0.001	0.000	0.810	0.693
TCGA-49-4487-01A-21R-1858-07	0.287	0.066	0.057	0.266	0	0.155	0.108	0.185	0.094	0	0.071	0	0.001	0.461	0.133	0.246	0	0.03	0.005	0.011	0	0	0.000	0.832	0.681
TCGA-49-4488-01A-01R-1755-07	0.04	0.031	0.092	0.15	0	0.233	0.001	0.111	0.072	0	0.04	0	0.016	0.296	0.112	0.285	0.085	0.023	0.021	0.013	0	0.002	0.000	0.698	0.764
TCGA-49-4510-01A-01R-1206-07	0.11	0	0.453	0.189	0	0.35	0	0.237	0.035	0	0.116	0	0.045	0	0.039	0.779	0.001	0.108	0.014	0.011	0	0.017	0.000	0.450	0.896
TCGA-53-A4EZ-01A-12R-A24X-07	0.146	0.074	0.058	0.083	0	0.169	0.005	0.213	0.066	0	0.049	0.015	0.049	0.162	0.078	0.351	0.076	0.034	0.064	0	0	0.001	0.000	0.469	0.885
TCGA-55-1594-01A-01R-0946-07	0.106	0	0.181	0.071	0	0.419	0	0.15	0.038	0	0.076	0.034	0	0.315	0.167	0.586	0.004	0	0.057	0	0	0.039	0.000	0.607	0.815
TCGA-55-7574-01A-11R-2039-07	0.263	0.307	0.07	0.207	0	0.37	0.064	0.119	0	0	0.06	0	0	0.294	0.068	0.413	0	0.076	0.131	0	0	0	0.000	0.774	0.732
TCGA-55-7994-01A-11R-2187-07	0.271	0.022	0	0.422	0	0.228	0.121	0.363	0.105	0	0.273	0.026	0.008	0.804	0.411	0.937	0.078	0.076	0.094	0	0	0.002	0.000	0.727	0.736
TCGA-55-7995-01A-11R-2187-07	0.273	0.094	0	0.492	0	0.388	0.016	0.451	0.266	0	0.279	0	0.141	1.356	0.539	0.65	0	0	0.046	0	0	0	0.000	0.817	0.666
TCGA-55-8085-01A-11R-2241-07	0.083	0.136	0.192	0.342	0	0.313	0.05	0.336	0.215	0	0.162	0.006	0.125	0.524	0.271	0.624	0	0.073	0	0	0	0.011	0.000	0.752	0.734
TCGA-55-8091-01A-11R-2241-07	0.01	0.022	0.141	0.051	0	0.684	0	0.033	0.172	0	0.165	0.06	0.059	0.394	0.219	0.813	0.091	0.167	0.332	0	0	0.05	0.000	0.671	0.784
TCGA-55-8096-01A-11R-2241-07	0.169	0	0.512	0.101	0	0.294	0.029	0.024	0.091	0	0.137	0	0.063	1.228	0.101	0.798	0	0	0.173	0	0	0.003	0.000	0.772	0.700
TCGA-55-8204-01A-11R-2241-07	0.032	0.006	0.408	0.045	0	0.312	0.02	0.07	0	0	0.097	0	0.028	0.151	0.208	0.339	0.058	0.036	0.144	0	0	0.064	0.000	0.651	0.810
TCGA-55-8301-01A-11R-2287-07	0.024	0.262	0.156	0.246	0	0.636	0.046	0.236	0.142	0	0.225	0.027	0.071	0.723	0.601	0.557	0.013	0.061	0.179	0	0	0	0.000	0.815	0.683
TCGA-55-8302-01A-11R-2326-07	0.018	0.068	0.131	0.146	0	0.124	0.043	0.204	0.059	0	0.068	0.009	0.069	0.291	0.165	0.381	0	0.06	0.07	0	0	0.004	0.000	0.690	0.774
TCGA-55-A4DF-01A-11R-A24H-07	0.116	0.035	0.083	0.085	0	0.396	0.021	0.28	0.097	0	0.174	0	0	0.741	0.263	0.497	0	0.023	0.025	0	0	0	0.000	0.800	0.683
TCGA-62-A46S-01A-11R-A24H-07	0.039	0.003	0.222	0	0	0.483	0	0.161	0.079	0	0.095	0.054	0.102	0.106	0.137	0.496	0.005	0.038	0.075	0	0	0.011	0.000	0.542	0.853
TCGA-62-A46V-01A-11R-A24H-07	0.021	0	0.212	0.005	0.011	0.211	0	0.056	0	0	0.002	0.029	0.036	0.261	0.003	0.419	0.093	0.166	0.208	0	0	0.003	0.000	0.646	0.800
TCGA-64-1677-01A-01R-0946-07	0.074	0.05	0.077	0.074	0	0.191	0.048	0.113	0.035	0	0.028	0.018	0	0.159	0.117	0.163	0	0.026	0.023	0	0	0.003	0.000	0.773	0.726
TCGA-64-5774-01A-01R-1628-07	0.023	0.003	0.22	0.065	0	0.262	0	0.16	0.151	0	0.003	0.049	0	0.32	0.024	0.287	0	0.039	0	0.032	0	0.027	0.000	0.398	0.918
TCGA-67-3770-01A-01R-0946-07	0.002	0	0.136	0.047	0	0.342	0.017	0.029	0	0	0.027	0	0.015	0.153	0.082	0.44	0.018	0.096	0.125	0	0.001	0.022	0.000	0.716	0.762
TCGA-69-A59K-01A-11R-A262-07	0.047	0.02	0.378	0.116	0	0.135	0.064	0.171	0.051	0	0.039	0	0.015	0.206	0.088	0.393	0	0.079	0.071	0	0	0.01	0.000	0.568	0.848
TCGA-73-4662-01A-01R-1206-07	0.179	0.39	0.306	0.137	0	0.787	0.05	0.426	0.105	0.042	0	0.001	0.318	0.6	0.073	0.758	0.155	0.239	0.176	0	0	0	0.000	0.702	0.779
TCGA-73-4668-01A-01R-1206-07	0	0.069	0.332	0.208	0	0.258	0	0.194	0.074	0	0.033	0	0	0.888	0.006	0.436	0	0.182	0.322	0	0	0.016	0.000	0.642	0.792
TCGA-78-7158-01A-11R-2039-07	0.025	0.054	0	0	0	0.321	0	0.062	0.052	0	0.012	0.029	0.078	0.24	0.05	0.3	0.083	0.037	0.263	0	0	0	0.000	0.505	0.867
TCGA-78-7542-01A-21R-2066-07	0.098	0	0.041	0	0	0.197	0	0.377	0.15	0	0.029	0.145	0	0.449	0.353	0.444	0	0.281	0.071	0	0	0.011	0.000	0.613	0.799
TCGA-78-8640-01A-11R-2403-07	0.09	0	0.065	0.08	0	0.226	0	0.126	0.088	0	0.078	0.016	0.008	0.398	0.107	0.24	0	0	0.044	0	0	0.004	0.000	0.724	0.740
TCGA-78-8660-01A-11R-2403-07	0.049	0.067	0.034	0.223	0	0.372	0.022	0.257	0.08	0	0.156	0.041	0.015	0.558	0.146	0.494	0	0.009	0.104	0	0	0	0.000	0.764	0.715
TCGA-86-7713-01A-11R-2066-07	0.171	0	0.526	0.016	0	0.264	0.039	0.118	0.062	0	0.124	0	0.067	0.277	0.135	0.436	0	0.164	0.004	0	0	0.021	0.000	0.477	0.886
TCGA-86-8054-01A-11R-2241-07	0.172	0	0.101	0.013	0	0.343	0.031	0.06	0.012	0	0.096	0	0.039	0.221	0.058	0.447	0.016	0.04	0.132	0	0	0.024	0.000	0.460	0.890
TCGA-86-A4JF-01A-11R-A24X-07	0.111	0	0.258	0.098	0	0.236	0	0.27	0.08	0	0.041	0.123	0.04	0.392	0.191	0.529	0	0.157	0.24	0	0	0.024	0.000	0.449	0.894
TCGA-91-8497-01A-11R-2403-07	0.098	0.302	0.355	0.084	0	0.251	0	0.206	0.121	0	0.021	0.024	0.019	0.27	0.033	0.444	0.046	0.063	0.251	0	0	0	0.000	0.752	0.769
TCGA-91-A4BD-01A-11R-A24H-07	0.031	0.009	0.038	0.054	0	0.192	0	0.081	0.045	0	0.003	0.062	0.294	0.075	0.023	0.562	0	0.089	0.209	0	0	0	0.000	0.714	0.758
TCGA-95-7039-01A-11R-1949-07	0.037	0	0.013	0.093	0	0.204	0.01	0.121	0.059	0	0.031	0	0	0.253	0.096	0.338	0	0.027	0.055	0	0	0	0.000	0.573	0.827
TCGA-95-7043-01A-11R-1949-07	0.084	0	0.112	0.046	0	0.096	0	0.059	0.044	0	0.011	0.003	0.009	0.052	0.029	0.181	0	0.015	0.016	0	0	0	0.002	0.317	0.950
TCGA-J2-8194-01A-11R-2241-07	0.057	0	0.15	0.011	0	0.423	0.046	0.037	0	0	0.037	0.003	0.125	0.129	0.128	0.764	0.056	0.033	0.127	0	0	0.135	0.000	0.490	0.874
TCGA-J2-A4AD-01A-11R-A24H-07	0.132	0	0.214	0.064	0	0.139	0	0.205	0	0	0.049	0	0	0.316	0	0.338	0	0.135	0.142	0	0	0.01	0.000	0.372	0.928
TCGA-MN-A4N1-01A-11R-A24X-07	0.056	0	0.014	0.035	0	0.056	0	0.192	0.112	0	0.036	0.001	0.066	0.664	0.01	0.24	0	0.08	0.031	0	0	0	0.000	0.635	0.777
TCGA-MN-A4N5-01A-11R-A24X-07	0.094	0.066	0.051	0.347	0	0.273	0.061	0.427	0.143	0	0.187	0	0.029	0.292	0.278	0.519	0	0	0.05	0	0	0	0.000	0.455	0.890
TCGA-MP-A4SV-01A-11R-A24X-07	0.092	0.055	0.151	0.228	0	0.501	0.029	0.231	0.142	0	0.213	0	0.033	0.329	0.199	0.455	0	0.09	0.05	0.019	0	0	0.000	0.708	0.760
TCGA-MP-A4SW-01A-21R-A24X-07	0.068	0.213	0.091	0.144	0	0.219	0	0.219	0.22	0	0.079	0.001	0.075	0.546	0.052	0.551	0	0.074	0.174	0	0	0	0.000	0.747	0.741
TCGA-MP-A4T9-01A-11R-A24X-07	0.15	0	0	0.137	0	0.309	0	0.196	0.056	0	0.159	0	0.077	0.624	0.128	0.676	0	0.102	0.306	0	0	0.014	0.000	0.680	0.770
TCGA-MP-A4TA-01A-21R-A24X-07	0.1	0	0.27	0.151	0	0.372	0	0.284	0.155	0	0.122	0.034	0	0.415	0.096	0.492	0	0.088	0	0	0	0.003	0.000	0.503	0.868
TCGA-MP-A4TF-01A-11R-A262-07	0.004	0.006	0.18	0.025	0	0.073	0.032	0.142	0.02	0	0.074	0	0	0.283	0.087	0.134	0	0.059	0.001	0.01	0	0.023	0.000	0.516	0.860
TCGA-MP-A5C7-01A-11R-A262-07	0.022	0	0.118	0.035	0	0.26	0	0.196	0.022	0	0.085	0.041	0.051	0.096	0.044	0.279	0.038	0.09	0.061	0	0	0	0.002	0.312	0.953
TCGA-05-4382-01A-01R-1206-07	0.089	0.055	0.463	0.147	0	0.499	0.065	0.265	0.19	0	0.075	0.006	0	1.366	0.329	1.591	0	0	0.102	0	0	0.125	0.000	0.781	0.692
TCGA-05-4405-01A-21R-1858-07	0.025	0.076	0.175	0.045	0	0.153	0	0.06	0.052	0	0.049	0	0	0.455	0.026	0.391	0	0.011	0.118	0	0	0.001	0.000	0.789	0.700
TCGA-05-4420-01A-01R-1206-07	0.106	0	0.258	0.031	0	0.476	0	0.101	0.019	0	0.176	0	0.023	0.228	0.025	0.582	0	0.056	0	0.065	0	0.233	0.000	0.449	0.894
TCGA-05-4422-01A-01R-1206-07	0.165	0.059	0.511	0.601	0	0.414	0.011	0.276	0.218	0	0.168	0	0.062	0.239	0.206	0.626	0.026	0.021	0	0.021	0	0.004	0.000	0.710	0.767
TCGA-05-4424-01A-22R-1858-07	0.158	0	0.06	0.107	0	0.344	0.008	0.065	0.037	0	0.043	0.006	0.037	0.378	0.13	0.657	0	0.086	0.217	0	0	0.026	0.000	0.694	0.765
TCGA-05-4427-01A-21R-1858-07	0.056	0	0.1	0.058	0	0.287	0.042	0.098	0	0	0.056	0	0.067	0.267	0.114	0.506	0.035	0.041	0.061	0	0	0.003	0.000	0.688	0.770
TCGA-05-4432-01A-01R-1206-07	0.024	0.091	0.425	0.136	0	0.486	0.01	0.199	0.172	0	0.145	0.016	0	0.939	0.195	0.701	0	0.008	0.021	0.028	0	0.053	0.000	0.807	0.697

Input Sample	B cells naive	B cells memory	Plasma cells	T cells CD8	T cells CD4 naive	T cells CD4 memory resting	T cells CD4 memory activated	T cells follicular helper	T cells regulatory (Tregs)	T cells gamma delta	NK cells resting	NK cells activated	Monocytes	Macrophages M0	Macrophages M1	Macrophages M2	Dendritic cells resting	Dendritic cells activated	Mast cells resting	Mast cells activated	Eosinophils	Neutrophils	P-value	Correlation	RMSE
TCGA-38-4630-01A-01R-1206-07	0.192	0	0.016	0.175	0	0.669	0	0.133	0.093	0	0.075	0	0.023	0.56	0.276	0.669	0.013	0.033	0	0	0	0	0.000	0.622	0.797
TCGA-44-A47G-01A-21R-A24H-07	0.086	0.245	0.186	0.284	0	0.406	0.072	0.159	0.101	0	0.093	0	0.075	0.387	0.204	0.674	0.044	0.079	0.171	0	0	0.006	0.000	0.780	0.734
TCGA-50-5072-01A-21R-1858-07	0.153	0	0.147	0.051	0	0.241	0.014	0.102	0.051	0	0.099	0	0.028	0.368	0.09	0.414	0	0.074	0.093	0	0	0.02	0.000	0.589	0.827
TCGA-50-5931-01A-11R-1755-07	0.136	0	0.145	0.009	0	0.039	0	0.254	0	0	0.049	0	0	0.304	0.036	0.16	0	0.102	0.001	0.009	0.002	0	0.002	0.323	0.951
TCGA-50-6590-01A-12R-1858-07	0.06	0.061	0.139	0.182	0	0.437	0.067	0.064	0.163	0	0.055	0.078	0	0.647	0.268	0.597	0	0	0.037	0	0	0.002	0.000	0.801	0.687
TCGA-50-6595-01A-12R-1858-07	0.064	0	0.05	0.08	0	0.347	0.007	0.107	0.049	0	0.205	0	0.014	0.361	0.302	0.563	0	0.017	0.024	0	0	0.011	0.000	0.703	0.744
TCGA-55-6971-01A-11R-1949-07	0.009	0.141	0.179	0.181	0	0.384	0.001	0.118	0.111	0	0.013	0.089	0.04	0.384	0.115	0.35	0	0.022	0.126	0	0	0.026	0.000	0.789	0.722
TCGA-55-6975-01A-11R-1949-07	0.084	0	0.283	0.04	0	0.154	0.021	0.074	0.021	0	0.002	0.003	0.015	0.102	0.013	0.198	0	0.107	0.039	0	0	0.015	0.000	0.622	0.832
TCGA-55-6984-01A-11R-1949-07	0.123	0.081	0.039	0.057	0	0.238	0	0.077	0.082	0	0.042	0	0.007	0.131	0.007	0.114	0	0.061	0	0.03	0	0.034	0.000	0.547	0.846
TCGA-55-6986-01A-11R-1949-07	0.069	0.047	0.043	0.048	0	0.245	0	0.07	0.05	0	0.047	0	0.021	0.104	0.044	0.464	0.058	0.029	0.088	0	0	0.01	0.000	0.711	0.764
TCGA-55-7281-01A-11R-2039-07	0	0.092	0.026	0.139	0	0.347	0	0.106	0.098	0	0	0.072	0.147	0.262	0.113	0.671	0.257	0.163	0.095	0	0	0.009	0.000	0.685	0.773
TCGA-55-7573-01A-11R-2039-07	0.199	0.094	0.013	0.071	0	0.332	0.005	0.188	0.043	0	0.058	0	0.035	0.093	0.092	0.266	0.133	0.09	0.153	0	0	0.001	0.000	0.505	0.870
TCGA-55-7725-01A-11R-2170-07	0.106	0.019	0	0	0	0.352	0.022	0.148	0	0.042	0.021	0	0	0.211	0.114	0.299	0.01	0.051	0.143	0	0	0.011	0.000	0.623	0.807
TCGA-55-7903-01A-11R-2170-07	0.077	0	0.007	0.185	0	0.336	0.04	0.116	0.043	0	0.17	0.008	0.066	0.268	0.169	0.354	0.04	0.056	0.119	0	0	0	0.000	0.691	0.767
TCGA-55-7910-01A-11R-2170-07	0.093	0	0.283	0	0	0.146	0.016	0.073	0.037	0	0.043	0	0	0.146	0.027	0.097	0	0.044	0.02	0.002	0	0.019	0.000	0.481	0.888
TCGA-55-8089-01A-11R-2241-07	0.14	0.071	0.05	0.499	0	0.451	0.171	0.24	0.149	0	0.106	0.328	0.081	0.601	0.566	0.992	0	0.07	0.359	0	0	0.005	0.000	0.741	0.731
TCGA-55-8299-01A-11R-2287-07	0.163	0.004	0.113	0.449	0	0.283	0.134	0.308	0.139	0	0.352	0.052	0.071	0.307	0.17	0.626	0	0.566	0.251	0	0	0.019	0.000	0.790	0.713
TCGA-55-8512-01A-11R-2403-07	0.118	0	0.041	0.111	0	0.244	0	0.174	0	0	0.16	0	0.113	0.333	0.018	0.398	0	0.066	0.047	0.023	0	0.083	0.002	0.279	0.972
TCGA-55-8615-01A-11R-2403-07	0.052	0	0.014	0.032	0	0.074	0	0.207	0.087	0	0.028	0.028	0.028	0.622	0.012	0.285	0	0.036	0.024	0	0	0.002	0.000	0.544	0.839
TCGA-62-8399-01A-21R-2326-07	0.106	0	0.263	0.009	0	0.662	0.011	0.113	0	0	0.142	0	0.12	0.117	0.062	0.473	0	0.166	0.141	0.026	0	0.053	0.004	0.267	0.969
TCGA-64-1680-01A-02R-0946-07	0.072	0	0.079	0.044	0	0.258	0	0.077	0	0.003	0.009	0	0.011	0.055	0.059	0.38	0.011	0.01	0.176	0	0	0.006	0.000	0.392	0.920
TCGA-67-3773-01A-01R-0946-07	0.072	0	0.064	0.087	0	0.384	0.003	0.091	0.031	0	0.042	0.002	0.007	0.128	0.113	0.451	0.11	0.009	0.198	0	0	0.003	0.000	0.534	0.855
TCGA-67-3774-01A-01R-0946-07	0.018	0.096	0.044	0.086	0	0.471	0	0.162	0.026	0	0.061	0.001	0.035	0.178	0.136	0.514	0	0.077	0.116	0	0	0.024	0.000	0.643	0.797
TCGA-69-7763-01A-11R-2170-07	0.064	0.014	0.196	0.071	0	0.353	0.006	0.065	0.031	0	0.087	0	0.018	0.258	0.068	0.386	0	0.053	0.083	0	0	0.016	0.000	0.470	0.885
TCGA-69-7764-01A-11R-2170-07	0.178	0	0.262	0.084	0	0.391	0	0.096	0	0	0.015	0	0.014	0.122	0.082	0.178	0	0.031	0	0.037	0	0	0.000	0.567	0.843
TCGA-69-7980-01A-11R-2187-07	0.146	0	0.067	0.405	0	0.468	0.015	0.315	0.169	0	0.092	0	0.104	0.212	0.242	0.88	0	0.071	0.119	0	0	0.022	0.000	0.682	0.773
TCGA-69-8253-01A-11R-2287-07	0.175	0.132	0.553	0.123	0	0.431	0	0.163	0	0	0.027	0.074	0.053	0.292	0.011	0.476	0.047	0.233	0.147	0	0	0.041	0.000	0.469	0.891
TCGA-71-6725-01A-11R-1858-07	0.054	0.023	0.027	0.021	0	0.151	0	0.104	0.033	0	0	0.027	0.033	0.017	0.006	0.985	0.04	0.124	0.01	0.02	0.01	0.033	0.000	0.433	0.904
TCGA-73-4658-01A-01R-1755-07	0.061	0.051	0.067	0.116	0	0.399	0.039	0.18	0.041	0	0	0.031	0.158	0.299	0.105	0.797	0.177	0.091	0.082	0.002	0	0.02	0.000	0.716	0.755
TCGA-75-6205-01A-11R-1755-07	0.043	0.045	0.046	0.124	0	0.557	0.073	0.074	0.025	0	0.12	0	0.071	0.233	0.199	0.736	0.03	0.009	0.141	0	0.009	0	0.000	0.834	0.685
TCGA-78-7161-01A-11R-2039-07	0.114	0	0.276	0.104	0	0.284	0	0.074	0.083	0	0.082	0.041	0	0.23	0.035	0.279	0	0.192	0.078	0	0	0.044	0.002	0.297	0.958
TCGA-78-7162-01A-21R-2066-07	0.144	0.036	0.182	0.114	0	0.667	0	0.235	0.068	0	0.151	0.073	0.041	0.111	0.151	0.662	0.051	0.167	0.254	0	0	0	0.000	0.613	0.819
TCGA-80-5607-01A-31R-1949-07	0.046	0.01	0.184	0.117	0	0.273	0.056	0.139	0	0	0.018	0.015	0.025	0.07	0.141	0.386	0.051	0.039	0.169	0	0	0	0.000	0.480	0.883
TCGA-80-5608-01A-31R-1949-07	0.034	0	0.152	0.132	0	0.433	0.001	0.249	0.037	0	0.046	0.082	0	0.345	0.129	0.544	0	0.043	0.101	0	0	0.058	0.000	0.524	0.857
TCGA-86-7954-01A-11R-2187-07	0.162	0	0.053	0.25	0	0.678	0.067	0.392	0.199	0	0.144	0.004	0.139	0.37	0.859	1.188	0	0.107	0.323	0	0	0.014	0.000	0.751	0.716
TCGA-05-4384-01A-01R-1755-07	0.038	0	0.122	0.011	0	0.237	0	0.055	0	0	0.119	0	0.037	0.361	0.039	0.627	0.019	0.028	0.073	0	0	0.028	0.000	0.590	0.816
TCGA-05-4402-01A-01R-1206-07	0.071	0.118	0.329	0.11	0	0.312	0	0.149	0.163	0	0.071	0	0.162	0.586	0.17	0.874	0	0.209	0.251	0	0	0.053	0.000	0.724	0.758
TCGA-05-4403-01A-01R-1206-07	0.044	0.065	0.149	0.105	0	0.384	0	0.209	0.226	0	0.099	0	0.066	0.958	0.209	1.185	0.081	0	0.331	0	0	0.004	0.000	0.713	0.743
TCGA-05-4417-01A-22R-1858-07	0.109	0.011	0.13	0.122	0	0.453	0.078	0.174	0.076	0	0.027	0.024	0.053	0.386	0.101	0.449	0.027	0.128	0.118	0	0	0.012	0.000	0.811	0.710
TCGA-35-3615-01A-01R-0946-07	0.132	0	0.226	0.002	0	0.247	0	0.204	0.028	0	0.031	0.001	0	0.33	0.068	0.766	0	0.144	0.178	0	0	0.026	0.000	0.776	0.724
TCGA-38-4631-01A-01R-1755-07	0.079	0	0.226	0.071	0	0.185	0.003	0.074	0.03	0.004	0.045	0	0	0.247	0.127	0.194	0	0.017	0.002	0.017	0	0.084	0.000	0.448	0.894
TCGA-44-6777-01A-11R-1858-07	0.104	0.043	0.196	0.093	0	0.411	0.016	0.068	0.13	0	0.048	0.006	0.157	0.577	0.111	1.005	0.137	0.091	0.199	0	0	0.026	0.000	0.839	0.677
TCGA-44-7661-01A-11R-2066-07	0.107	0.032	0	0	0	0.695	0.18	0.252	0.124	0	0.028	0.093	0.229	0.384	0.154	1.091	0.04	0.494	0.245	0	0	0.013	0.000	0.784	0.718
TCGA-44-7662-01A-11R-2066-07	0.152	0	0.28	0.148	0	0.437	0.009	0.158	0.099	0	0.268	0	0.035	0.734	0.26	0.718	0	0	0.105	0.025	0	0.009	0.000	0.801	0.700
TCGA-44-7672-01A-11R-2066-07	0.169	0.189	0.267	0.185	0	0.607	0.017	0.195	0.272	0	0.14	0.078	0.096	0.529	0.214	0.749	0.024	0.157	0.205	0	0	0.004	0.000	0.829	0.707
TCGA-44-A479-01A-31R-A24H-07	0.143	0.019	0.391	0.224	0	0.511	0.058	0.267	0.038	0.059	0.048	0	0.091	0.212	0.272	0.591	0	0.014	0.074	0	0	0	0.000	0.781	0.733
TCGA-50-5932-01A-11R-1755-07	0.1	0	0.157	0.036	0	0.209	0	0.041	0.023	0	0.06	0.013	0.051	0.294	0.041	0.402	0	0.028	0.081	0	0	0.004	0.000	0.409	0.912
TCGA-50-5942-01A-21R-1755-07	0.105	0	0.257	0.093	0	0.366	0	0.052	0.04	0	0.018	0.014	0.062	0.023	0.048	0.245	0.039	0.009	0.3	0	0	0	0.000	0.532	0.864
TCGA-55-6642-01A-11R-1858-07	0	0.018	0.423	0.068	0	0.38	0.096	0.026	0.033	0	0.053	0.014	0	0.324	0.07	0.271	0	0	0.076	0	0	0.008	0.000	0.749	0.764
TCGA-55-6978-01A-11R-1949-07	0.062	0	0.101	0.594	0	0.47	0.093	0.409	0.174	0.043	0.005	0.154	0.017	0.423	0.566	1.192	0.011	0.058	0.196	0	0	0.016	0.000	0.753	0.721
TCGA-55-6983-01A-11R-1949-07	0.395	0.451	0.04	0.247	0	0.531	0	0.347	0.08	0	0	0.014	0	0.64	0.096	0.539	0	0.048	0.152	0	0	0	0.000	0.804	0.701
TCGA-55-7283-01A-11R-2039-07	0.104	0	0.034	0.135	0	0.338	0.025	0.106	0.029	0	0.042	0.023	0.04	0.16	0.076	0.499	0.024	0.089	0.174	0	0	0.018	0.000	0.625	0.810
TCGA-55-7284-01B-11R-2241-07	0.084	0.008	0	0.162	0	0.564	0	0.09	0.136	0	0.204	0	0.056	0.791	0.15	0.88	0.013	0.022	0.249	0	0	0.014	0.000	0.568	0.828
TCGA-55-8205-01A-11R-2241-07	0.037	0.172	0.081	0.387	0	0.777	0.168	0.301	0.001	0	0.107	0.062	0.202	0.601	0.492	1.34	0.028	0.014	0.127	0	0	0.012	0.000	0.725	0.743
TCGA-55-8511-01A-11R-2403-07	0.089	0.173	0.08	0.214	0	0.262	0.045	0.25	0.095	0	0.117	0	0.053	0.287	0.2	0.453	0	0	0.033	0	0	0.008	0.000	0.764	0.729
TCGA-55-8514-01A-11R-2403-07	0.056	0.013	0.046	0.025	0	0.143	0	0.148	0.086	0	0.067	0	0.097	0.147	0.076	0.497	0	0.055	0.071	0	0	0.002	0.000	0.549	0.844
TCGA-55-8614-01A-11R-2403-07	0.001	0.052	0.119	0.078	0	0.259	0	0.125	0.049	0	0	0.031	0.001	0.425	0.118	0.253	0	0.032	0.182	0	0	0.017	0.000	0.506	0.865
TCGA-62-A46Y-01A-11R-A24H-07	0.098	0.04	0.06	0.205	0	0.37	0	0.215	0.077	0	0.102	0	0.1	0.322	0.142	0.823	0.151	0.065	0.141	0	0	0.018	0.000	0.337	0.945
TCGA-62-A472-01A-11R-A24H-07	0.033	0	0.002	0.104	0	0.195	0.008	0.163	0.037	0	0.013	0.04	0.019	0.1	0.089	0.258	0.047	0.011	0.138	0	0	0.009	0.000	0.673	0.781
TCGA-64-1676-01A-01R-0946-07	0	0.012	0.051	0.162	0	0.327	0.039	0.125	0.047	0	0.038	0.015	0.241	0.238	0.126	0.768	0.08	0	0.052	0	0	0	0.000	0.529	0.852
TCGA-64-5779-01A-01R-1628-07	0.046	0.088	0.324	0.094	0	0.38	0.027	0.114	0.043	0	0.034	0	0.002	0.18	0.086	0.082	0	0	0	0.005	0	0	0.000	0.615	0.818
TCGA-64-5815-01A-01R-1628-07	0.026	0.071	0	0	0	0.36	0	0.076	0.129	0	0.088	0	0.021	0.646	0.001	0.45	0.009	0.173	0.049	0	0	0.019	0.000	0.831	0.648
TCGA-67-4679-01B-01R-1755-07	0.001	0.056	0	0.08	0	0.412	0	0.051	0.042	0	0.019	0.021	0.215	0.174	0.037	0.474	0.122	0.069	0.502	0	0	0.011	0.000	0.642	0.799
TCGA-69-7761-01A-11R-2170-07	0	0.098	0.344	0.201	0	0.608	0.112	0.172	0.023	0	0.116	0	0	0.429	0.387	0.39	0.021	0.009	0.191	0	0	0.029	0.000	0.732	0.751
TCGA-69-7978-01A-11R-2187-07	0.023	0.189	0.349	0.487	0	0.704	0.129	0.187	0.135	0	0.209	0	0.044	0.367	0.405	1.124	0.204	0.02	0.166	0	0	0.041	0.000	0.801	0.717
TCGA-71-8520-01A-11R-2403-07	0.035	0	0.087	0	0	0.208	0.002	0.098	0.046	0	0.094	0	0.012	0.431	0.045	0.302	0	0.069	0.079	0	0	0.001	0.000	0.676	0.766
TCGA-73-4675-01A-01R-1206-07	0.057	0.101	0.054	0.079	0	0.175	0	0.062	0.133	0	0.007	0.055	0.078	0.982	0	0.596	0.068	0.155	0.243	0	0	0.016	0.000	0.646	0.775
TCGA-75-6206-01A-11R-1755-07	0.073	0	0.066	0.023	0	0.129	0	0.064	0.029	0	0.041	0	0	0.665	0.038	0.503	0	0.026	0.054	0	0	0.002	0.000	0.696	0.729
TCGA-75-7030-01A-11R-1949-07	0.109	0.003	0.137	0.101	0	0.402	0.021	0.042	0.006	0	0.055	0	0.016	0.169	0.073	0.233	0.017	0.049	0.081	0	0	0.002	0.000	0.696	0.778
TCGA-78-7155-01A-11R-2039-07	0.077	0	0.006	0.093	0	0.173	0.005	0.062	0.011	0.017	0.001	0.005	0.004	0.109	0.081	0.318	0	0	0	0.012	0	0	0.000	0.521	0.855
TCGA-78-7537-01A-11R-2066-07	0	0.077	0.335	0.198	0	0.332	0	0.07	0.144	0	0.008	0.084	0.013	0.225	0.021	0.471	0.021	0.077	0.174	0	0	0	0.000	0.432	0.904
TCGA-83-5908-01A-21R-2287-07	0.138	0	0.009	0.561	0	0.56	0.118	0.466	0.142	0	0.226	0.156	0.015	0.226	0.511	0.727	0.009	0.046	0.195	0	0	0.01	0.000	0.640	0.786
TCGA-86-7955-01A-11R-2187-07	0.176	0	0.112	0.083	0	0.093	0	0.168	0.113	0	0.083	0.039	0	0.175	0.052	0.158	0	0.101	0.08	0	0	0	0.000	0.338	0.942
TCGA-86-8056-01A-11R-2241-07	0.181	0	0.092	0.071	0	0.518	0.006	0.105	0.007	0	0.087	0	0.093	0.489	0.111	0.604	0.087	0.044	0.208	0	0	0.026	0.000	0.598	0.820
TCGA-86-8073-01A-11R-2241-07	0.076	0.028	0.082	0.179	0	0.202	0.004	0.136	0.028	0	0.075	0	0.051	0.168	0.09	0.976	0.004	0.024	0.23	0	0	0.024	0.000	0.616	0.807
TCGA-91-6840-01A-11R-1949-07	0.143	0	0.049	0.103	0	0.28	0	0.187	0.024	0	0.044	0.009	0.024	0.254	0.148	0.269	0	0.017	0.053	0	0	0	0.000	0.652	0.788
TCGA-97-7547-01A-11R-2039-07	0.204	0	0.103	0.065	0	0.303	0.014	0.17	0.067	0	0.018	0	0.087	0.248	0.115	0.468	0	0.039	0.123	0	0	0	0.000	0.640	0.804
TCGA-97-7554-01A-11R-2039-07	0.033	0.058	0.294	0.057	0	0.38	0.002	0.094	0.045	0	0.071	0	0	0.344	0.116	0.55	0	0.024	0.09	0	0	0.018	0.000	0.744	0.749
TCGA-97-8177-01A-11R-2287-07	0.072	0.256	0.031	0.304	0	0.22	0	0.33	0.21	0	0.021	0.083	0.205	0.548	0.073	0.981	0.198	0.173	0.325	0.076	0	0.037	0.000	0.762	0.736
TCGA-05-4244-01A-01R-1107-07	0	0.045	0.029	0.03	0	0.643	0	0.215	0.042	0	0.17	0	0.243	0.819	0.052	0.781	0.191	0.151	0.182	0	0.011	0.001	0.006	0.250	0.990
TCGA-05-4390-01A-02R-1755-07	0.054	0	0.129	0.016	0	0.173	0.028	0.036	0.036	0	0.094	0	0	0.541	0.033	0.3	0	0	0	0.044	0	0.016	0.000	0.649	0.773
TCGA-05-5423-01A-01R-1628-07	0.071	0.063	0.05	0.036	0	0.337	0.008	0.09	0.069	0.005	0	0.034	0.093	0.242	0.077	0.638	0.185	0.091	0.218	0	0.043	0.049	0.000	0.762	0.737
TCGA-44-2655-01A-01R-0946-07	0.252	0.25	0.175	0.116	0	0.758	0	0.262	0.114	0	0.009	0.032	0.054	0.265	0.077	0.788	0.266	0.079	0.171	0	0	0.017	0.000	0.633	0.811
TCGA-44-2665-01A-01R-0946-07	0	0.025	0	0	0	0.633	0	0.055	0.048	0	0.076	0	0.008	0.693	0	1.022	0.082	0.267	0.202	0	0	0.015	0.000	0.513	0.858
TCGA-44-3396-01A-01R-1206-07	0.102	0.094	0.596	0.198	0	0.941	0.09	0.264	0.023	0	0.249	0	0.044	1.354	0.362	1.04	0	0.102	0	0.327	0	0.156	0.000	0.874	0.652
TCGA-44-6779-01A-11R-1858-07	0.019	0	0.003	0.301	0	0.303	0.006	0.133	0.125	0	0.085	0.078	0.025	0.138	0.245	0.505	0	0.007	0.152	0	0	0.022	0.000	0.597	0.813
TCGA-44-7670-01A-11R-2066-07	0.112	0.072	0.141	0.167	0	0.1	0.044	0.281	0.029	0	0.008	0.086	0.032	0.772	0.17	0.386	0	0.014	0.112	0	0.012	0	0.000	0.617	0.801
TCGA-49-6743-01A-11R-1858-07	0.101	0	0.211	0.077	0	0.302	0.008	0.112	0.012	0	0.129	0	0	0.41	0.123	0.397	0	0.011	0.028	0.008	0	0.006	0.000	0.715	0.755
TCGA-49-6767-01A-11R-1858-07	0.02	0	0.007	0.227	0	0.368	0.066	0.077	0.122	0	0	0.115	0	0.59	0.181	0.346	0	0	0.061	0	0	0	0.000	0.632	0.787
TCGA-50-5933-01A-11R-1755-07	0.021	0.016	0.06	0.032	0	0.38	0.002	0.09	0.033	0	0.055	0	0.073	0.681	0.154	0.745	0	0	0.01	0.076	0	0.044	0.000	0.799	0.663
TCGA-50-5935-01A-11R-1755-07	0.019	0.037	0.05	0	0	0.556	0.01	0.056	0.114	0	0.067	0	0.036	0.372	0.054	0.303	0.054	0.163	0.068	0	0	0	0.000	0.720	0.750
TCGA-55-1592-01A-01R-0946-07	0.228	0	0.244	0.069	0	0.375	0	0.185	0	0	0.086	0	0.158	0.844	0.177	0.892	0.052	0	0.192	0	0	0.027	0.000	0.722	0.741
TCGA-55-1595-01A-01R-0946-07	0.112	0	0.26	0.16	0	0.403	0.026	0.243	0.071	0	0.131	0.018	0.028	0.204	0.162	0.693	0	0.079	0.116	0	0	0.009	0.000	0.727	0.761
TCGA-55-7911-01A-11R-2170-07	0.108	0.006	0.078	0.115	0	0.377	0.1	0.378	0.083	0	0.195	0.008	0.057	0.131	0.093	0.602	0	0.355	0.04	0	0	0.004	0.000	0.659	0.788
TCGA-55-8090-01A-11R-2241-07	0.099	0.11	0.097	0.025	0	0.517	0	0.186	0.127	0	0.045	0.027	0.14	0.434	0.128	0.859	0.088	0.027	0.127	0	0	0.022	0.000	0.623	0.806
TCGA-55-8616-01A-11R-2403-07	0.257	0.141	0.018	0.148	0	0.301	0	0.077	0.129	0	0.007	0	0.033	0.097	0.098	0.309	0	0.002	0.1	0	0	0.008	0.000	0.608	0.815
TCGA-55-8619-01A-11R-2403-07	0.244	0.13	0.113	0.27	0	0.29	0	0.236	0.134	0	0.128	0	0.071	0.509	0.111	0.698	0.02	0.017	0.147	0	0	0.069	0.000	0.664	0.787
TCGA-69-7765-01A-11R-2170-07	0.005	0.113	0.254	0	0	0.299	0.059	0.094	0.035	0	0.084	0	0.015	0.243	0.077	0.339	0.092	0.111	0.133	0	0	0.013	0.000	0.681	0.798
TCGA-73-4676-01A-01R-1755-07	0.079	0	0.006	0.081	0	0.181	0.067	0.037	0	0	0.032	0	0.095	0.311	0.028	0.591	0.003	0.057	0.186	0	0	0.019	0.000	0.629	0.795
TCGA-75-6214-01A-41R-1949-07	0	0	0.162	0.006	0	0.234	0	0.19	0.085	0	0.018	0.03	0	0.75	0.114	0.423	0	0.089	0.015	0.002	0	0.059	0.000	0.483	0.876
TCGA-75-7025-01A-12R-1949-07	0.034	0.471	0.043	0.189	0	0.355	0	0.279	0.081	0	0	0.092	0.207	0.06	0.033	0.639	0.13	0.236	0.359	0	0	0.005	0.000	0.690	0.788
TCGA-75-7027-01A-11R-1949-07	0.177	0	0.189	0.049	0	0.326	0.033	0.103	0.16	0	0.108	0	0.014	0.48	0.126	0.518	0	0.035	0.156	0	0	0.001	0.000	0.435	0.900
TCGA-78-7148-01A-11R-2039-07	0.081	0	0.137	0.163	0	0.121	0	0.164	0.151	0	0.05	0.059	0.047	0.156	0.1	0.3	0.016	0.016	0.118	0	0	0.004	0.000	0.542	0.852
TCGA-78-7152-01A-11R-2039-07	0.036	0.12	0.054	0.118	0	0.241	0	0.092	0.063	0	0.03	0	0	0.158	0.078	0.207	0.012	0.003	0.033	0	0	0.003	0.000	0.713	0.758

Input Sample	B cells naive	B cells memory	Plasma cells	T cells CD8	T cells CD4 naive	T cells CD4 memory resting	T cells CD4 memory activated	T cells follicular helper	T cells regulatory (Tregs)	T cells gamma delta	NK cells resting	NK cells activated	Monocytes	Macrophages M0	Macrophages M1	Macrophages M2	Dendritic cells resting	Dendritic cells activated	Mast cells resting	Mast cells activated	Eosinophils	Neutrophils	P-value	Correlation	RMSE
TCGA-78-7159-01A-11R-2039-07	0.105	0.003	0.24	0.078	0	0.271	0	0.107	0.117	0	0	0.05	0	0.318	0.072	0.229	0	0.066	0	0.038	0	0.055	0.000	0.587	0.830
TCGA-78-7535-01A-11R-2066-07	0	0.099	0.078	0.097	0	0.272	0	0.083	0.07	0	0.101	0.025	0	0.539	0.006	1.238	0.019	0.034	0	0.055	0	0.011	0.000	0.627	0.785
TCGA-86-7714-01A-12R-2170-07	0.016	0.012	0	0.019	0	0.225	0	0.011	0.009	0	0	0.048	0.05	0	0.016	0.291	0.228	0.122	0.16	0	0	0.003	0.000	0.533	0.854
TCGA-86-8280-01A-11R-2287-07	0.065	0.181	0.231	0.082	0	0.565	0	0.345	0.108	0	0.098	0	0.08	0.566	0.273	0.631	0.129	0.092	0.243	0	0	0	0.000	0.843	0.696
TCGA-86-8585-01A-11R-2403-07	0.038	0.081	0.025	0.132	0	0.172	0.006	0.129	0.046	0	0.04	0.01	0.023	0.527	0.078	0.236	0	0.041	0.033	0	0	0	0.000	0.827	0.657
TCGA-86-A456-01A-11R-A24H-07	0.069	0	0.128	0.075	0	0.364	0	0.199	0.01	0.011	0.021	0.01	0	0.436	0.138	0.703	0	0.101	0.018	0.129	0.008	0.002	0.000	0.529	0.851
TCGA-86-A4P8-01A-11R-A24X-07	0.048	0.041	0	0.355	0	0.791	0	0.212	0.047	0	0.2	0.009	0.09	0.732	0.165	1.115	0	0.007	0.293	0	0	0.008	0.000	0.683	0.764
TCGA-91-6829-01A-21R-1858-07	0.059	0	0.04	0.065	0	0.197	0.001	0.038	0.071	0	0.035	0.009	0.008	0.44	0.049	0.401	0	0.005	0.251	0	0	0.017	0.000	0.634	0.791
TCGA-91-6835-01A-11R-1858-07	0.181	0.44	0	0.319	0	0.531	0.085	0.241	0.139	0.1	0.094	0	0	0.784	0.297	0.984	0.008	0	0.115	0	0	0	0.000	0.869	0.653
TCGA-91-6847-01A-11R-1949-07	0.127	0	0.085	0.025	0	0.292	0	0.043	0.069	0	0.147	0.075	0.009	0.261	0.092	0.122	0.021	0	0.055	0	0	0	0.004	0.265	0.979
TCGA-91-6849-01A-11R-1949-07	0.088	0.044	0.254	0.015	0.009	0.023	0	0.101	0.026	0	0.084	0	0	0.522	0.016	0.267	0	0.054	0	0.006	0	0.01	0.000	0.715	0.739
TCGA-99-8028-01A-11R-2241-07	0.115	0.076	0.372	0.406	0	1.003	0.039	0.3	0.122	0	0.194	0	0	1.069	0.431	1.01	0	0.028	0.147	0	0	0	0.000	0.835	0.676
TCGA-05-4426-01A-01R-1206-07	0.107	0	0.01	0.055	0	0.552	0	0.03	0.253	0	0.061	0.049	0.166	0.835	0.077	0.602	0	0.001	0.067	0.042	0	0.033	0.000	0.605	0.805
TCGA-05-5420-01A-01R-1628-07	0.16	0	0.158	0.11	0	0.375	0.071	0.258	0.005	0.031	0	0.059	0.006	0.592	0.397	0.804	0.079	0	0.106	0	0	0.04	0.000	0.850	0.656
TCGA-05-5425-01A-02R-1628-07	0.198	0	0.176	0.254	0	0.247	0.148	0.204	0.043	0	0.085	0.01	0.047	0.362	0.246	0.639	0	0.183	0.129	0	0	0.052	0.000	0.700	0.770
TCGA-38-4628-01A-01R-1206-07	0.081	0	0	0.037	0	0.189	0	0.103	0.067	0	0	0.053	0.113	0.636	0.006	1.037	0.055	0.065	0.25	0	0	0.032	0.000	0.614	0.795
TCGA-38-7271-01A-11R-2039-07	0.098	0.735	0.042	0.442	0	0.614	0.078	0.144	0.176	0	0.079	0	0.009	0.48	0.124	0.47	0	0.057	0.096	0.004	0	0	0.000	0.785	0.715
TCGA-44-2656-01B-06R-A277-07	0.023	0	0.052	0.017	0	0.355	0.039	0.082	0	0.003	0	0.037	0.001	0.096	0.115	0.254	0.004	0.079	0.086	0	0.018	0.006	0.000	0.585	0.826
TCGA-44-2657-01A-01R-1107-07	0.311	0.18	0.255	0.305	0	0.414	0	0.322	0.139	0	0.002	0.04	0.007	0.182	0.208	0.523	0.026	0.019	0.272	0	0	0.001	0.000	0.812	0.719
TCGA-44-2662-01A-01R-0946-07	0.087	0	0.127	0	0	0.658	0.043	0.08	0.079	0	0.056	0	0.464	0.858	0.144	0.952	0	0	0.109	0	0.034	0.066	0.000	0.524	0.853
TCGA-44-3398-01A-01R-1107-07	0.104	0	0.113	0.099	0	0.745	0	0.179	0.029	0	0.02	0.158	0.12	0.123	0.09	1.158	0.606	0.3	0.558	0	0.096	0.063	0.000	0.330	0.946
TCGA-44-6774-01A-21R-1858-07	0.055	0	0.11	0	0	0.254	0.016	0.015	0.058	0	0.073	0	0.047	0.295	0.054	0.37	0	0.017	0.03	0.014	0	0.02	0.000	0.777	0.714
TCGA-50-5066-01A-01R-1628-07	0.044	0.019	0.292	0.335	0	0.207	0.126	0.169	0.023	0.047	0.012	0	0.011	0.315	0.195	0.555	0.033	0	0.066	0	0	0.008	0.000	0.776	0.731
TCGA-50-6597-01A-11R-1858-07	0.003	0.057	0.144	0.092	0	0.26	0	0.039	0.19	0.034	0	0.02	0.011	0.082	0.132	0.241	0.04	0.055	0.091	0	0	0	0.000	0.716	0.765
TCGA-55-5899-01A-11R-1628-07	0.047	0	0.131	0.026	0	0.295	0.014	0.145	0.06	0	0.103	0	0	0.456	0.179	0.412	0	0	0.052	0	0	0	0.000	0.705	0.750
TCGA-55-6972-01A-11R-1949-07	0.029	0	0.149	0.03	0	0.035	0	0.025	0.006	0	0.042	0.013	0.005	0.004	0.01	0.099	0	0.029	0.008	0	0	0	0.000	0.351	0.937
TCGA-55-6982-01A-11R-1949-07	0.022	0.054	0.116	0.015	0	0.172	0	0.086	0.034	0	0.03	0.007	0	0.351	0.165	0.338	0.018	0.021	0.052	0	0	0.006	0.000	0.796	0.688
TCGA-55-8092-01A-11R-2241-07	0	0.174	0.598	0.403	0	0.319	0.075	0.19	0.101	0	0.048	0.057	0.033	0.159	0.244	0.364	0	0	0.04	0	0	0	0.000	0.716	0.772
TCGA-55-8513-01A-11R-2403-07	0.06	0.199	0.047	0.026	0	0.17	0	0.188	0.014	0	0.071	0	0.037	0.467	0.049	0.771	0.044	0.121	0.138	0	0	0.019	0.000	0.685	0.763
TCGA-55-A48Y-01A-11R-A24H-07	0.123	0	0.269	0	0	0.236	0.026	0.113	0.083	0	0.058	0	0	0.444	0.07	0.295	0	0.033	0.008	0.009	0	0.038	0.000	0.570	0.835
TCGA-55-A493-01A-11R-A24H-07	0.221	0	0.006	0.152	0	0.465	0.117	0.21	0.15	0	0.209	0.02	0.045	0.448	0.166	0.533	0.037	0.052	0.033	0	0	0	0.000	0.787	0.709
TCGA-55-A4DG-01A-11R-A24H-07	0.229	0.164	0.168	0.205	0	0.307	0.012	0.211	0	0	0.009	0	0.034	0.158	0.108	0.377	0	0.038	0.187	0	0	0	0.000	0.598	0.829
TCGA-62-A46O-01A-11R-A24H-07	0.096	0	0.296	0	0	0.067	0.002	0.062	0.022	0.006	0.034	0.004	0	0.112	0.014	0.081	0	0.087	0.03	0	0	0	0.002	0.312	0.951
TCGA-64-1679-01A-21R-2066-07	0.019	0.027	0.372	0.03	0	0.221	0	0.07	0.102	0	0.001	0.01	0	1.098	0.121	0.713	0.135	0.101	0.169	0.007	0	0.061	0.000	0.794	0.672
TCGA-64-5775-01A-01R-1628-07	0.009	0	0.021	0.074	0	0.154	0	0.081	0.075	0	0	0.043	0.011	0.531	0.045	0.816	0	0	0	0.076	0	0.015	0.000	0.371	0.948
TCGA-73-4666-01A-01R-1206-07	0.037	0	0.009	0.686	0	0.316	0.465	0.341	0.136	0	0.44	0	0.088	0.436	0.575	1.155	0.003	0.007	0.241	0	0	0.017	0.000	0.675	0.770
TCGA-75-6207-01A-11R-1755-07	0.05	0	0.04	0.034	0	0.306	0.014	0.057	0.028	0	0.044	0	0.045	0.342	0.015	0.446	0.008	0.099	0.071	0	0	0.033	0.000	0.515	0.859
TCGA-78-7145-01A-11R-2039-07	0.025	0.021	0.007	0.004	0	0.218	0.014	0.06	0.029	0	0	0.01	0.07	0.411	0.034	0.28	0.122	0.085	0.191	0	0	0.005	0.000	0.598	0.813
TCGA-78-7153-01A-11R-2039-07	0.067	0.017	0.16	0.128	0	0.242	0	0.089	0.05	0	0.024	0.022	0.018	0.067	0.036	0.179	0.004	0.017	0.051	0	0	0	0.000	0.497	0.873
TCGA-78-7160-01A-11R-2039-07	0.141	0.009	0.034	0.173	0	0.389	0.036	0.134	0.07	0	0	0.02	0.029	0.265	0.131	0.637	0.001	0.139	0.072	0	0	0.012	0.000	0.733	0.742
TCGA-78-7539-01A-11R-2066-07	0.05	0.012	0.058	0.512	0	0.606	0.054	0.316	0.157	0	0	0.052	0.094	0.545	0.399	0.603	0.178	0	0.214	0	0	0	0.000	0.688	0.766
TCGA-86-8278-01A-11R-2287-07	0	0.073	0.322	0.12	0	0.791	0	0.161	0.005	0	0.201	0.01	0.033	0.42	0.196	0.685	0.093	0.164	0.398	0	0	0.038	0.000	0.381	0.924
TCGA-86-8281-01A-11R-2287-07	0.153	0.034	0.41	0.068	0	0.205	0	0.157	0.108	0	0.05	0.012	0.019	0.256	0.078	0.433	0.067	0.058	0.134	0	0	0	0.000	0.519	0.870
TCGA-86-8673-01A-11R-2403-07	0.001	0.027	0.169	0.095	0	0.197	0	0.115	0.171	0	0.102	0.05	0	0.383	0.051	0.263	0	0.005	0.001	0.003	0	0.005	0.000	0.674	0.772
TCGA-91-6828-01A-11R-1858-07	0.038	0.023	0.063	0.101	0	0.458	0.037	0.059	0	0	0.056	0	0.053	0.129	0.126	0.51	0.065	0	0.042	0	0	0.03	0.000	0.720	0.753
TCGA-95-8039-01A-11R-2241-07	0.074	0.089	0	0.018	0	0.735	0	0.155	0.179	0	0.055	0.085	0.097	0.057	0.105	0.744	0.182	0.258	0.291	0	0	0.031	0.000	0.718	0.764
TCGA-95-8494-01A-11R-2326-07	0	0.062	0	0.025	0	0.089	0.007	0.07	0.086	0	0.047	0.044	0.232	0.141	0	0.283	0.186	0.154	0.106	0	0	0.01	0.000	0.610	0.816
TCGA-95-A4VN-01A-11R-A262-07	0.099	0.019	0	0.267	0	0.461	0.087	0.296	0.066	0	0.142	0.011	0.011	0.446	0.24	0.684	0	0.079	0.115	0	0	0.035	0.000	0.797	0.699
TCGA-95-A4VP-01A-21R-A262-07	0.323	0.039	0.077	0.316	0	0.112	0.008	0.309	0.169	0	0.034	0.06	0.002	0.204	0.082	0.489	0	0.101	0.081	0	0	0.014	0.000	0.648	0.796
TCGA-97-7552-01A-11R-2039-07	0.1	0.49	0.167	0.51	0	0.555	0	0.184	0.181	0	0.164	0.018	0.049	0.512	0.274	0.442	0	0.029	0.141	0	0	0	0.000	0.793	0.713
TCGA-99-AA5R-01A-11R-A39D-07	0.071	0.433	0.168	0.349	0	0.568	0	0.303	0	0	0.1	0.055	0.018	0.596	0.203	0.566	0.014	0.009	0.166	0	0	0	0.000	0.761	0.734
TCGA-L9-A444-01A-21R-A24H-07	0.042	0.063	0.161	0.335	0	0.511	0.12	0.094	0.199	0	0.235	0	0.034	0.192	0.23	0.322	0	0	0.071	0	0	0	0.000	0.713	0.750
TCGA-MN-A4N4-01A-12R-A24X-07	0.021	0.054	0.121	0.091	0	0.115	0.009	0.165	0.131	0	0.059	0	0	0.929	0.069	0.257	0	0	0.135	0	0	0.011	0.000	0.796	0.662
TCGA-NJ-A7XG-01A-12R-A39D-07	0	0.029	0.097	0.046	0	0.382	0	0.061	0.047	0	0.007	0.083	0.019	0	0.017	0.227	0.058	0.117	0.29	0	0	0	0.002	0.316	0.952
TCGA-S2-AA1A-01A-12R-A39D-07	0.222	0.187	0.312	0.151	0	0.544	0	0.15	0.111	0	0	0.026	0.044	0.372	0.098	0.242	0.032	0.025	0.213	0	0	0	0.000	0.817	0.723
TCGA-49-AARQ-01A-11R-A41B-07	0.043	0	0	0.131	0	0.106	0.018	0.313	0.029	0	0.049	0.105	0.047	0.463	0.23	0.264	0	0	0.116	0	0	0	0.000	0.616	0.799
TCGA-50-8459-01A-11R-2326-07	0.178	0.083	0.11	0.119	0	0.238	0.033	0.076	0.151	0	0.121	0	0.209	0.554	0.062	0.725	0.123	0.05	0.211	0	0	0.012	0.000	0.793	0.714
TCGA-50-8460-01A-11R-2326-07	0.003	0.214	0.234	0.152	0	0.362	0	0.124	0.235	0	0.001	0.088	0.299	0.144	0.063	0.412	0.162	0.122	0.457	0	0	0	0.022	0.185	0.998
TCGA-55-8208-01A-11R-2241-07	0.196	0.144	0.218	0.155	0	0.518	0.047	0.232	0.207	0	0.098	0.02	0	0.759	0.384	1.005	0	0.023	0.122	0	0	0.059	0.000	0.840	0.675
TCGA-73-A9RS-01A-11R-A41B-07	0.056	0	0.084	0.059	0	0.057	0.022	0.126	0.033	0	0.029	0.001	0	0.102	0.041	0.17	0	0.01	0.09	0	0	0	0.000	0.401	0.916
TCGA-93-A4JO-01A-21R-A24X-07	0.064	0.247	0.074	0.215	0	0.325	0.002	0.3	0.065	0	0.172	0	0.091	0.447	0.187	0.664	0.015	0	0.11	0	0	0	0.000	0.820	0.697
TCGA-93-A4JQ-01A-11R-A24X-07	0.252	0.49	0.009	0.086	0	0.7	0.055	0.363	0.161	0	0.198	0	0	0.599	0.271	0.387	0	0.058	0.055	0	0	0	0.000	0.812	0.694
TCGA-95-7562-01A-11R-2241-07	0.055	0	0.101	0.011	0	0.329	0.026	0.097	0.091	0	0.075	0	0.01	0.398	0.204	0.463	0	0	0.09	0	0	0.015	0.000	0.754	0.722

Fig. S1. **Dataset from CIBERSORTx.** Dataset from CIBERSORTx of each patients representing absolute proportions of subsets of tumor infiltrating immune cells (TIICs) in each sample.

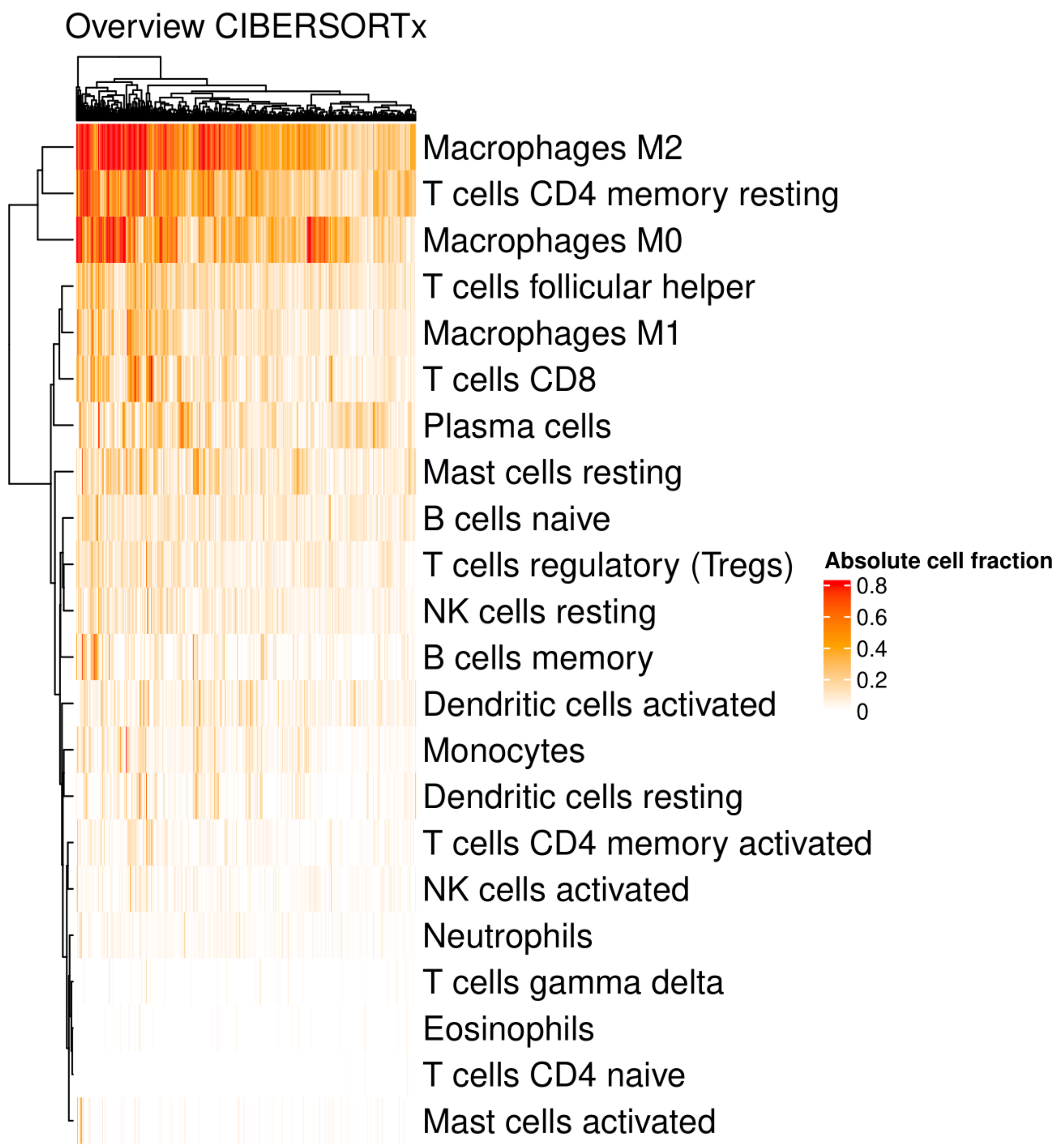

Fig. S2. **Heatmap of different cellular subtypes representing absolute cell fraction of different cellular subtypes.**

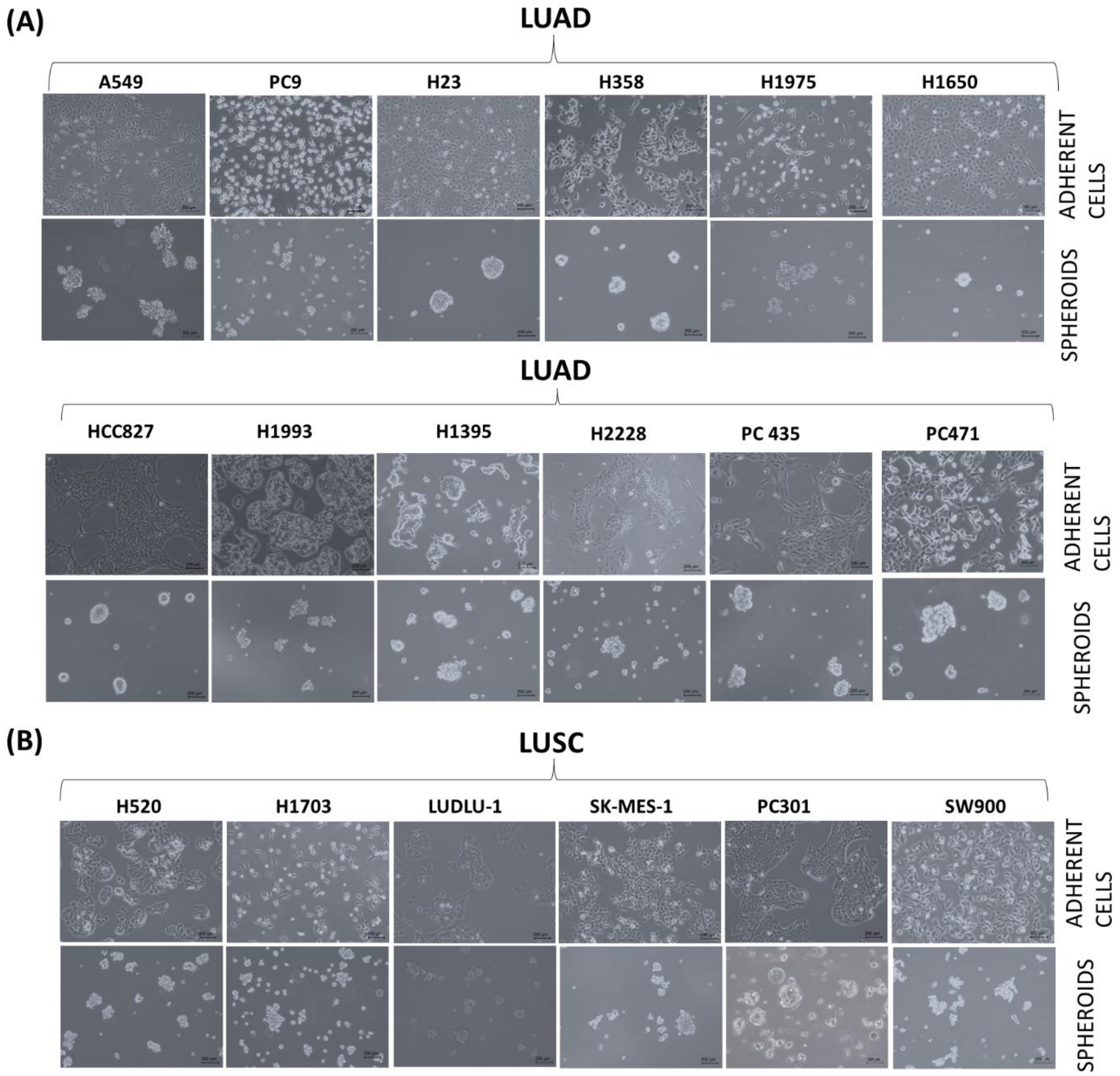

Fig. S3. **Representative images of the primary patient-derived cancer cells and cell lines grown under adherent conditions and suspension conditions.** (A) LUAD, lung adenocarcinoma cell lines. (B) LUSC, lung squamous cell carcinoma cell lines. Scale bar, 200µm.

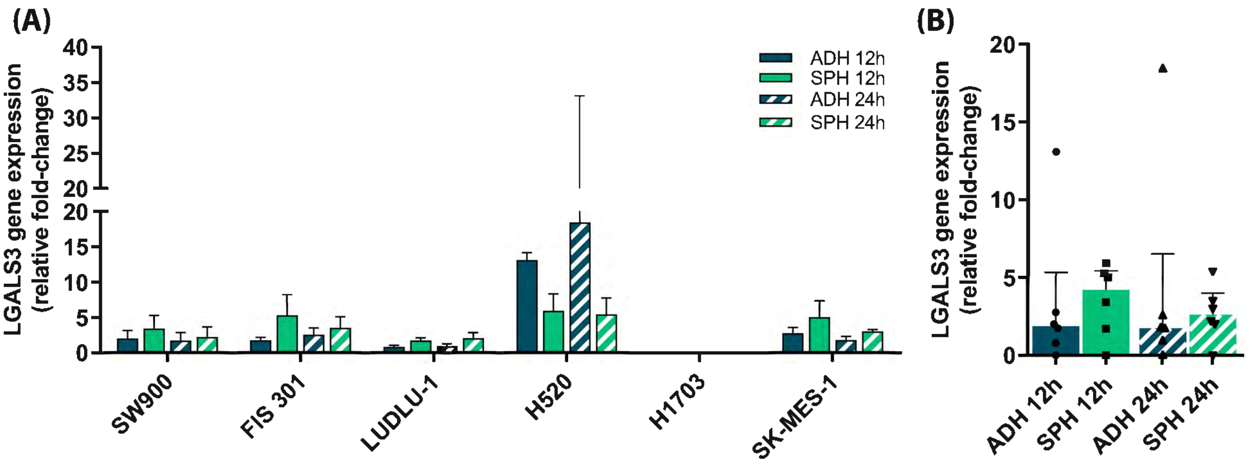

Fig. S4. **Transcription levels of *LGALS3* in tumorspheres vs. adherent-culture in different LUSC primary cultures and cell lines**. mRNA was measured by RTqPCR at 12 and 24 hours after cell seeding. (A) The results shown the relative fold-change gene expression of *LGALS3* to reference genes *ACTB*, *CDKN1B*, and *GUSB* of each cell line. Errors bars represent standard deviation (SD) of three different experiments. (B) The results shown are the median of relative fold-change gene expression of *LGALS3* to reference genes *ACTB*, CDKN1B, and *GUSB*. Errors bars represent interquartile range (IQR) of all samples (n=6). *ADH*, adherent; *SPH*, tumorspheres; *n*, sample size.

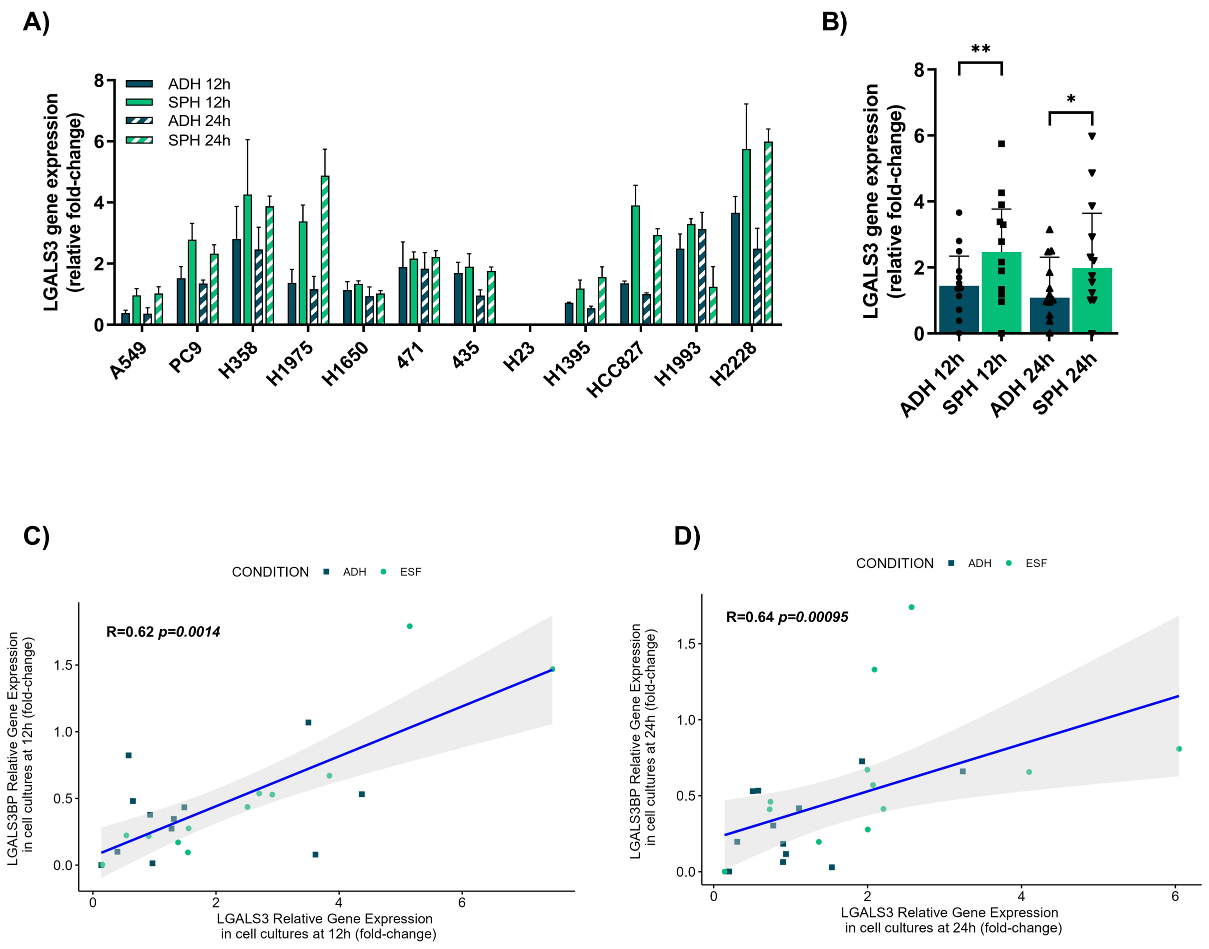

Fig. S5. **Transcription levels of *LGALS3BP* in tumorspheres vs. adherent-culture in different LUAD primary cultures and cell lines** mRNA was measured by RTqPCR at 12 and 24 hours after cell seeding. (A) The results shown the relative fold-change gene expression of *LGALS3BP* to reference genes *ACTB, CDKN1B,* and *GUSB* of each cell line. Errors bars represent standard deviation (SD) of three different experiments. (B) The results shown are the median of relative fold-change gene expression of *LGALS3* to reference genes *ACTB*, *CDKN1B*, and *GUSB*. Errors bars represent interquartile range (IQR) of all samples (n=12). (C) Correlation between *LGALS3BP* expression levels and *LGALS3* expression levels in LUAD tumor cell cultures at 12 hours after cell seeding (n=12). (D) Correlation between *LGALS3BP* expression levels and *LGALS3* expression levels in LUAD tumor cell cultures after 24h after cell seeding (n=12). R represents the Spearman correlation coefficient. Significance values were ** p ≤ 0.01. *ADH*, adherent; *SPH*, tumorspheres; *n*, sample size.

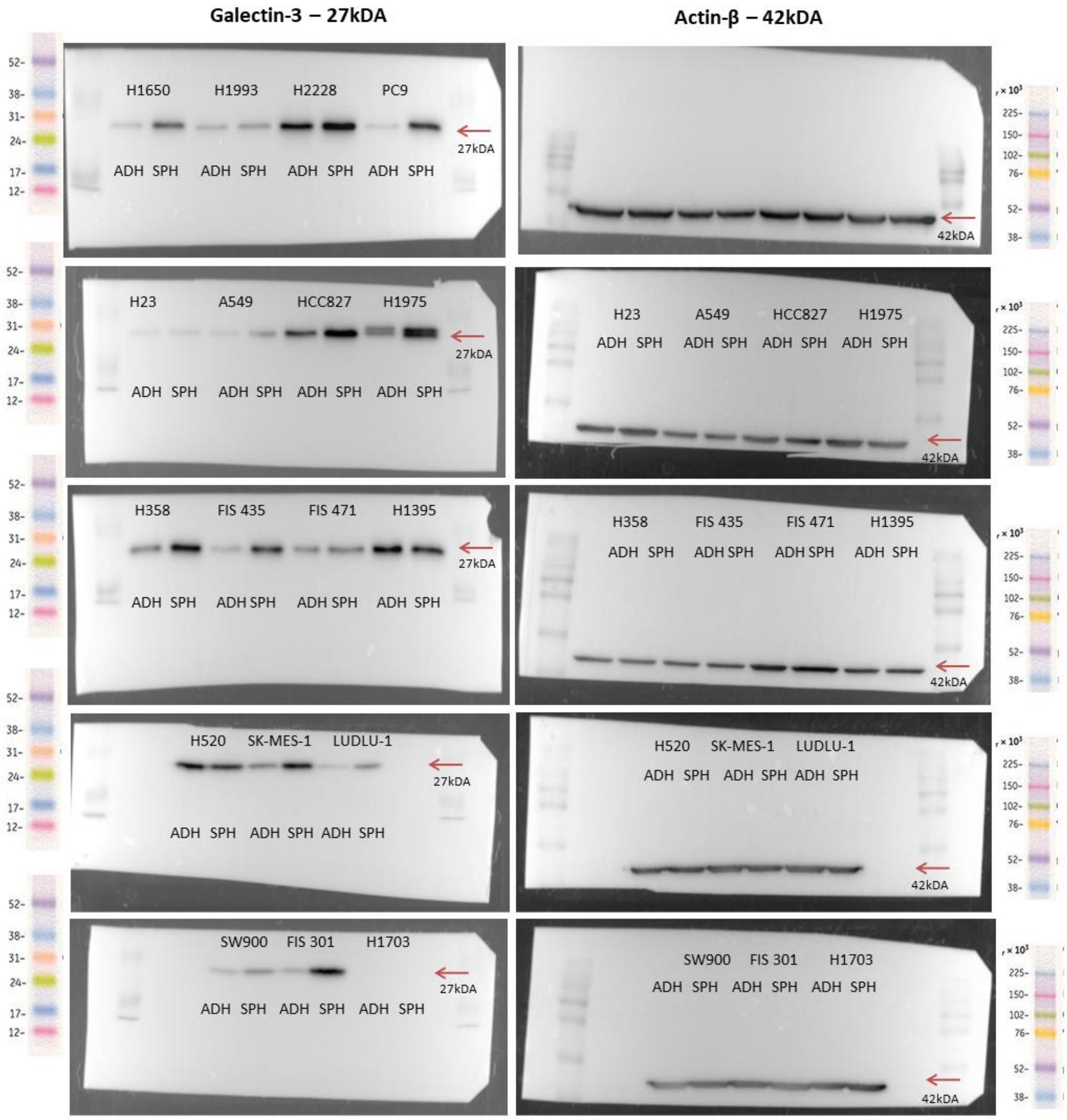

Fig. S6. **Original and complete immunoblots for B-actin and Galectin-3.**

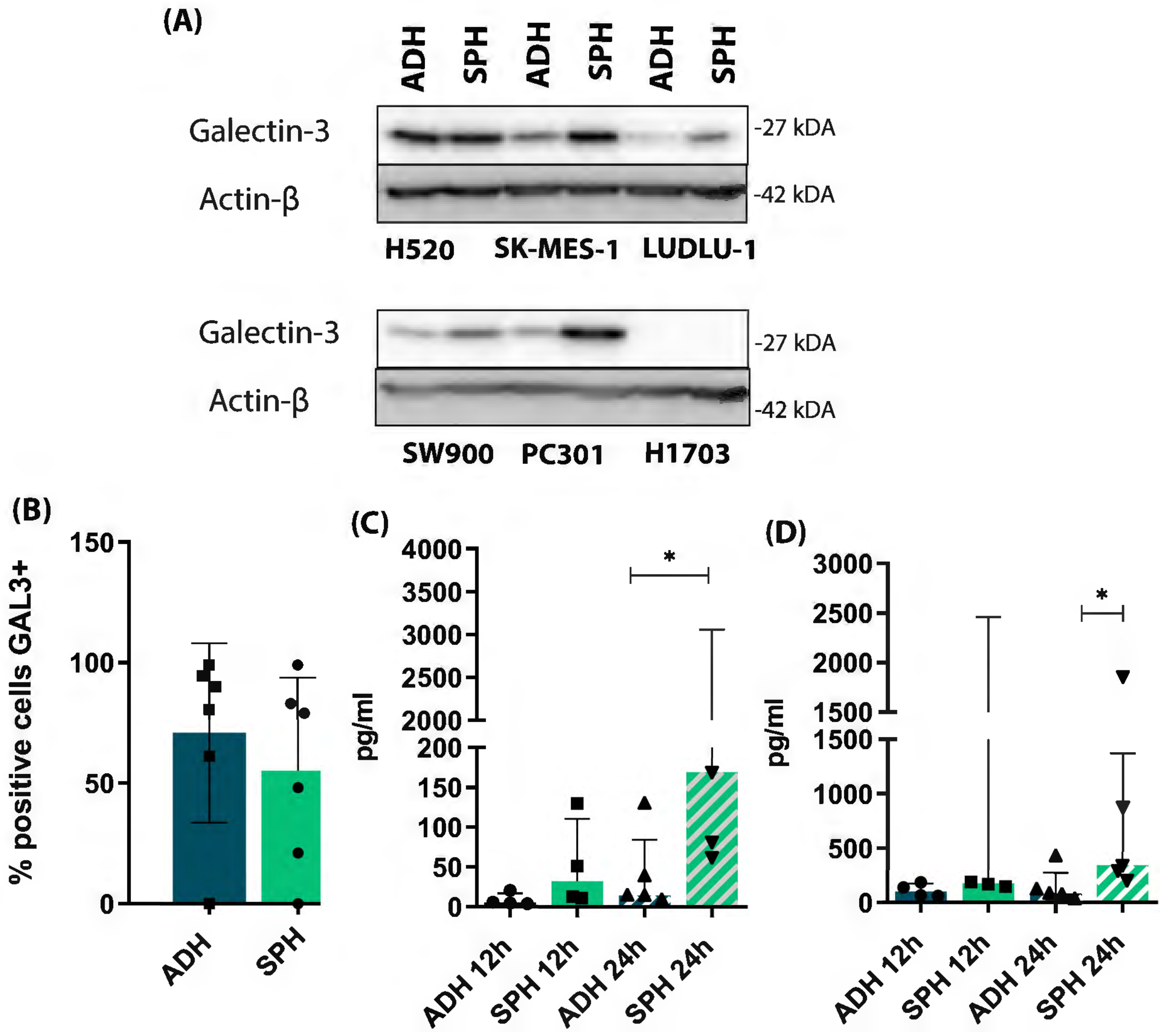

Fig. S7. **Expression of LGALS3 as protein level in LUSC cells.** (A) IBs showing the level of Galectin-3 in adherent cells and tumorspheres. Beta-actin (ACTB) was used as loading control. The electrophoreses were carried in 12% polyacrylamide gels, respectively. Ponceau-stained membranes were cropped according to the molecular weight of the corresponding proteins and the resulting strips were developed with the antibodies described under Materials and Methods. (B) Flow cytometry analysis of surface GAL3 in LUSC adherent cells and tumorspheres. The results shown are the median of all cell lines and primary culture. Errors bars represent interquartile range (IQR) of the median. (c,d) Immunoassay of sGAL-3 in LUAD adherent cells and tumorspheres analyzed by Luminex Technology at 12 and 24 hours after cell seeding. (C) Median levels of sGAL-3 of all cell lines and primary cultures at 12 and 24 hours after cell seeding 10.000 cells/ml (low density). (D) Median levels of sGAL-3 of all cell lines and primary cultures at 12 and 24 hours after cell seeding 100.000 cells/ml (high density). Errors bars represent IQR of the median of all cell lines and primary cultures (n=6). Significance values were * $p \leq 0.05$, ** $p \leq 0.01$. *ADH*, adherent; *SPH*, spheroid; *n*, sample size.

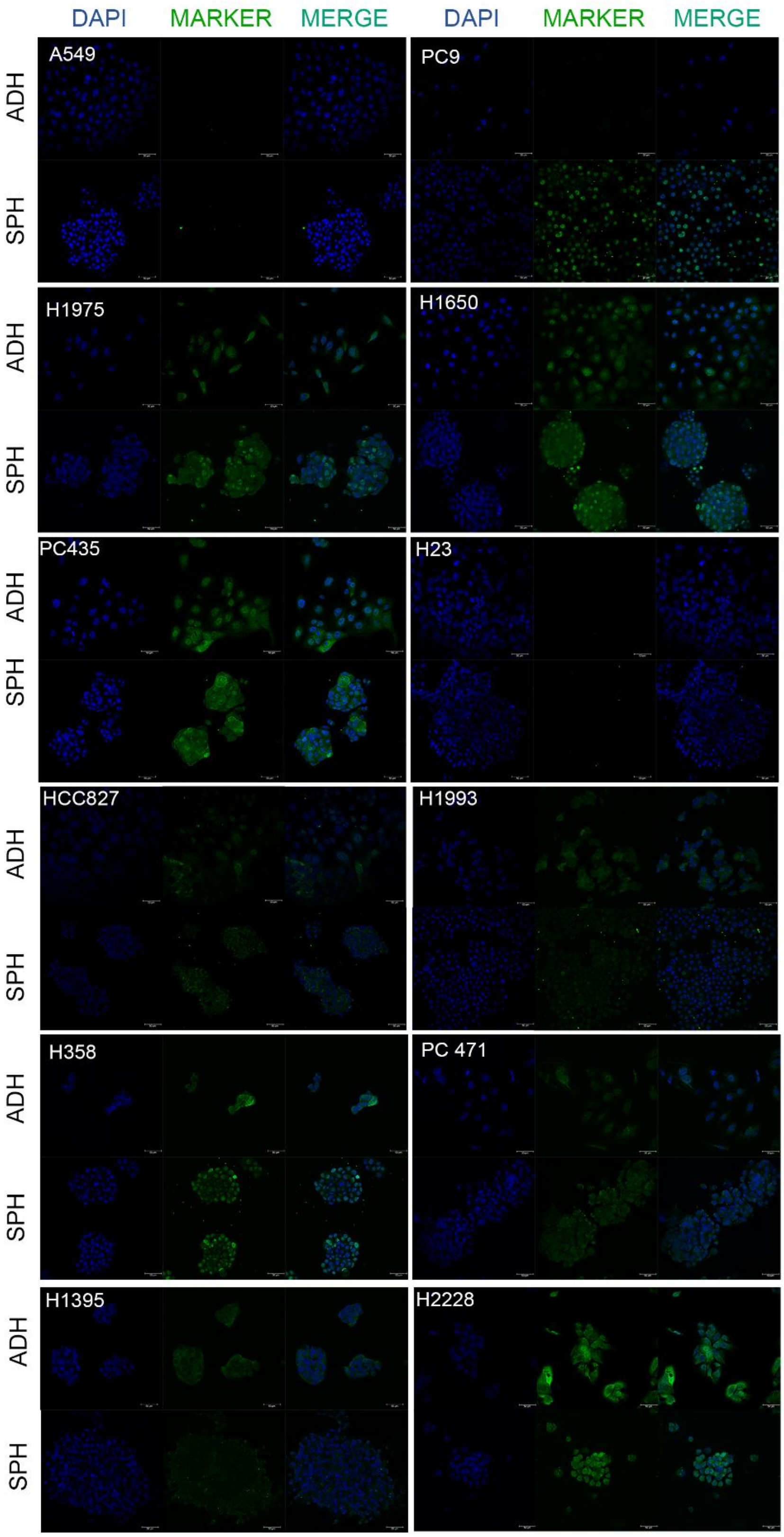

Fig. S8. **Representative immunofluorescence images of Gal-3 in tumorspheres and adherent-cultured cells from ADC patients**. Green channel in immunofluorescence shows the indicated antibody staining GAL3, blue channel shows DAPI staining, and merge shows all channels merged. Scale bar represents 50 μm.

Table S3. Clinicopathological characteristics of the LUSC early patients included in the study. *NS*, non-specified; *n*, sample size.

	In silico cohort		Plasma Validation Set	
	***n*= 313**	**%**	**n= 42**	**%**
Age at surgery (median, range)	67 [IQR 39–85]		69 [IQR 42–78]	
Gender				
Male	227	72.5	3	7.1
Female	86	27.5	39	92.9
Stage				
I	178	56.9	19	45.2
II	88	28.1	15	35.7
IIIA	47	15	8	19
Performance Status				
0	NS	NS	19	46.3
1			22	53.7
Smoking status				
Current	82	26.2	23	54.8
Former	201	64.2	17	40.5
Never	30	9.6	2	4.8
EGFR				
Mutated	NS	NS	1	2.4
WildType			27	64.3
NS			14	33.3
KRAS				
Mutated	NS	NS	2	4.8
WildType			25	64.3
NS			15	35.7
Relapse				
No	192	61.3	23	54.8
Yes	83	26.5	19	45.2
NS	38	12.1		
Exitus				
No	168	53.7	25	59.5
Yes	145	46.3	17	40.5

In silico set

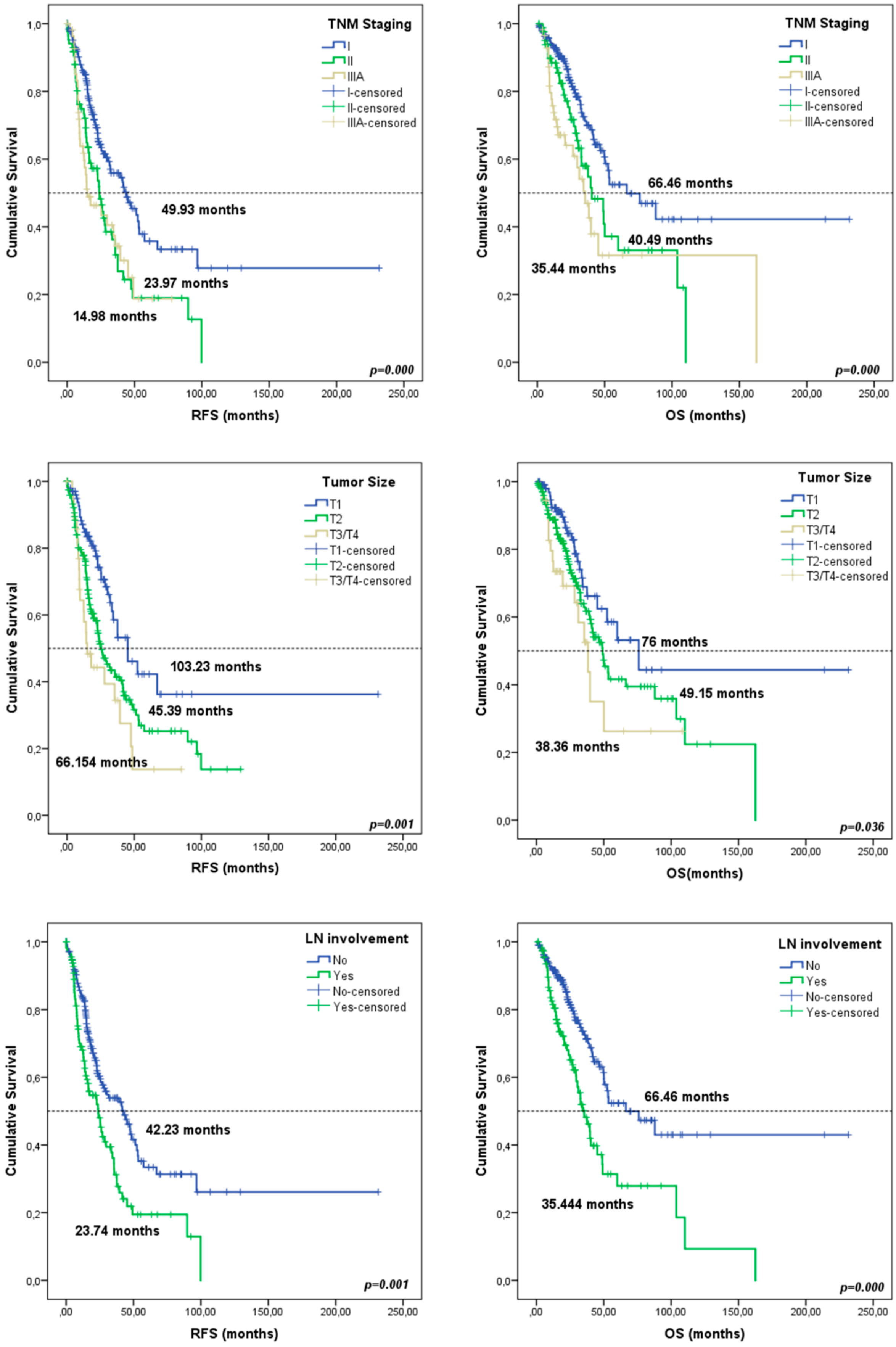

Fig. S9. **Kaplan-Meier survival curves according to clinicopathological variables from TCGA in silico set**. P-values were obtained using the log-rank test. TNM Staging, I (n=195), II (n=86), III (n=57); Tumor Size, T1 (n=103), T2 (n=199), T3/T4 (n=36); LN Involvement, No (n=216), Yes (n=115). *TNM*, tumor node metastasis; *T,* tumor size; *LN*, lymph node.

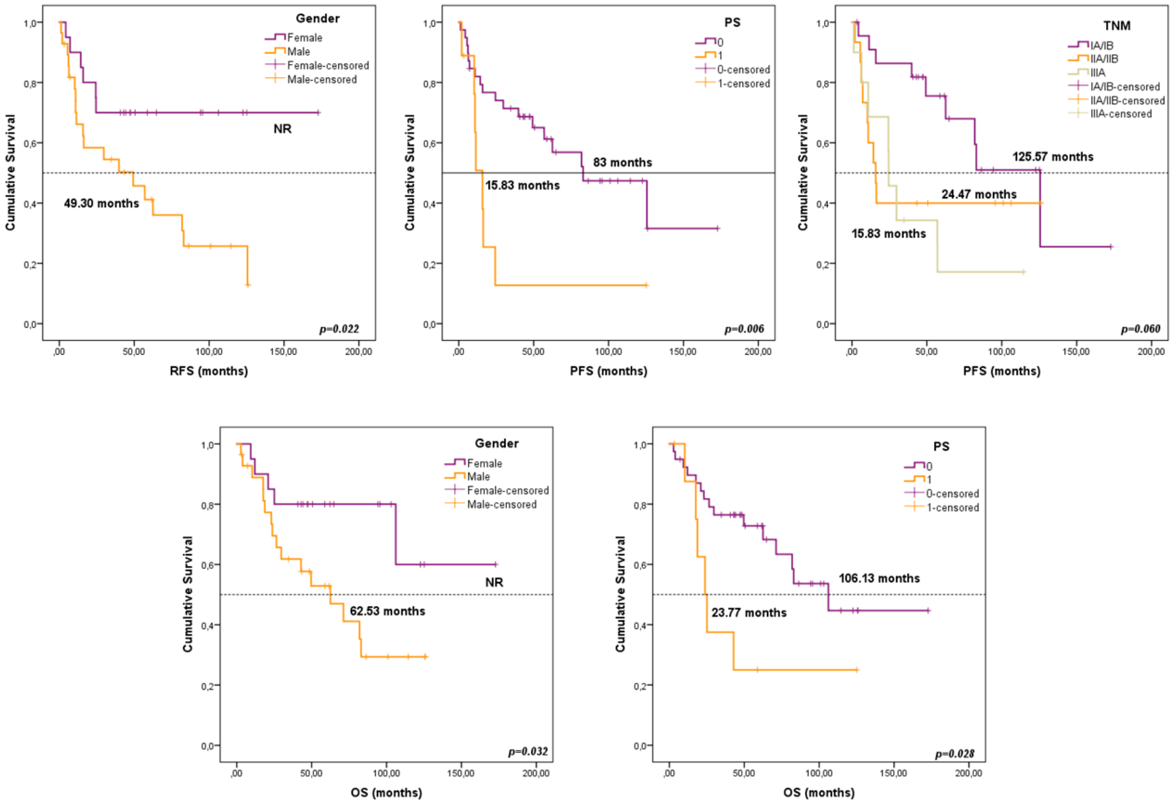

Fig. S10. **Kaplan-Meier survival curves according to clinicopathological variables in validation set**. P-values were obtained using the log-rank test. Gender, Female (n=20), Male (n=28); PS, 0 (n=39), 1 (n=9); TNM Staging, IA/IB (n=23), IIA/IIB (n=15), IIIA (n=10). *RFS*, relapse-free survival; *OS*, overall survival.

Table S4**.** Patient's characteristics of advanced-stage LUSC cohort. *n*, sample size

**Patient characteristics**	**LUAD advanced cohort**	
	***n* = 13**	**%**
Age at surgery (median, range)	[IQR -]	
Gender		
Male	10	76.9
Female	3	23.1
Stage		
III	6	47.2
IVA	2	15.4
IVB	5	38.5
Performance Status		
0-1	11	84.6
2	2	15.4
Smoking status		
Current	8	61.5
Former	4	30.8
Never	1	7.7
Progression		
Yes	12	92.3
No	1	7.7
Exitus		
Yes	11	84.6
No	2	15.4

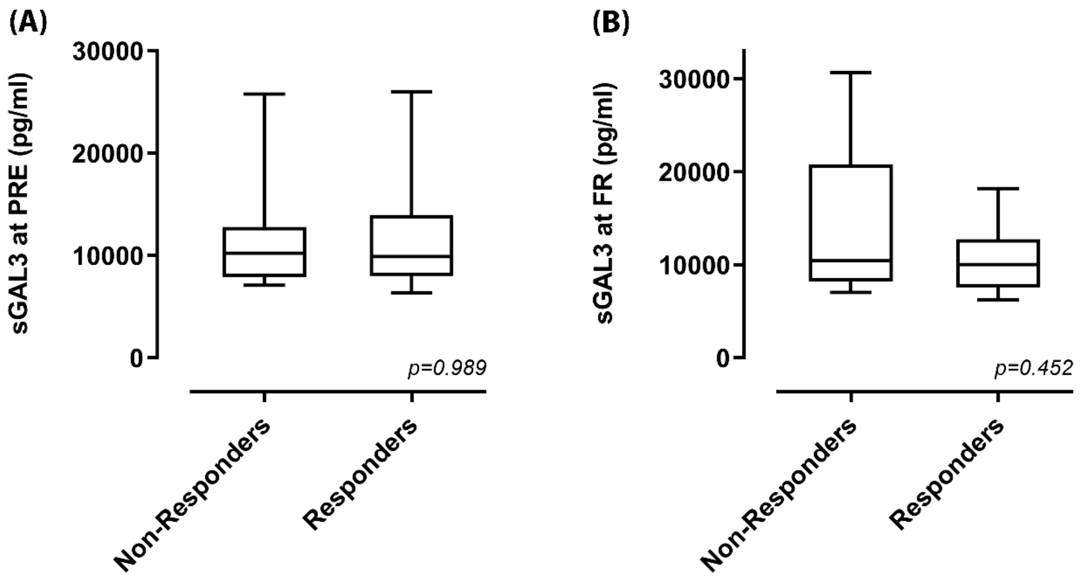

Fig. S11. **Analysis of predictive value in terms of Overall Response Rate (ORR) of sGal-3 in LUAD advanced cohort.** (A) sGAL-3 concentrations at pretreatment in patients with tumor response (n=15) and patients without tumor response (n=19). (B) sGAL-3 concentrations at first tumor evaluation in patients with tumor response (n=10) and patients without tumor response (n=16). P-values were obtained using the Mann-Whitney test. *PRE*, pretreatment; *FR*, first response assessment; *n*, sample size.

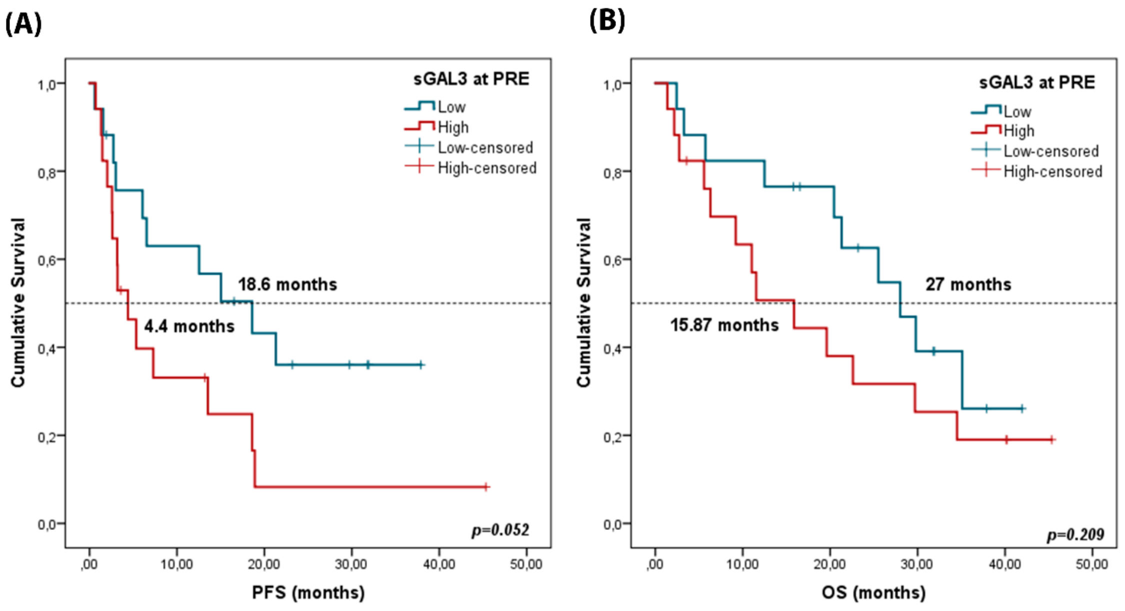

Fig. S12**. Kaplan-Meier survival curves according to sGAL-3 concentrations at pretreatment (PRE).** (A) Progression-free survival (PFS) stratified in high (n= 17) vs. low sGAL-3 levels (n=17). (B) Overall survival (OS) stratified in high (n= 17) vs. low sGAL-3 levels (n=17). P-values were obtained using the log-rank test. *PRE*, pretreatment; *PFS*, progression-free survival; *OS*, overall survival; *n*, sample size.

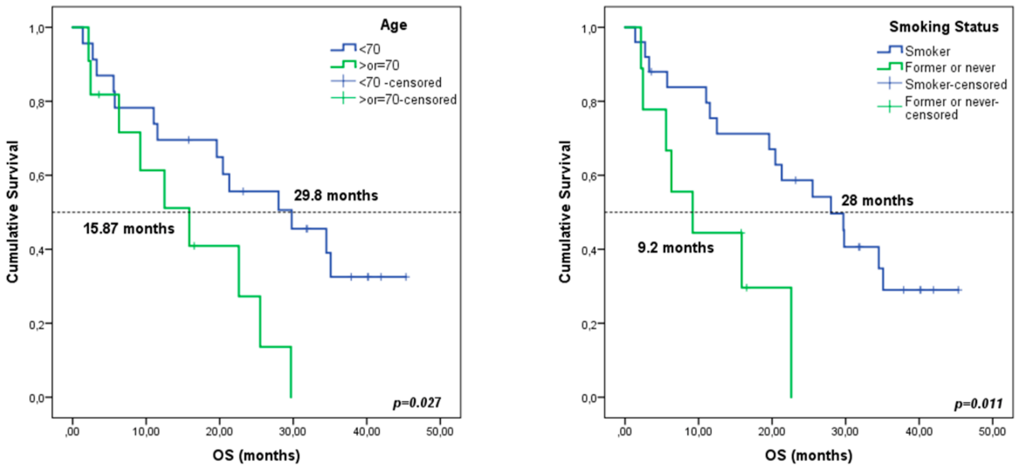

Fig. S13. **Kaplan-Meier survival curves according to clinicopathological variables in LUAD advanced cohort.** P-values were obtained using the log-rank test. Age, <70 (n=23), ≥70 (n=11); Smoking Status, Smoker (n=25), Former or never (n=9). *OS*, overall survival; *n*, sample size.
